# Molecular Trajectories Leading to the Alternative Fates of Duplicate Genes

**DOI:** 10.1371/journal.pone.0038958

**Published:** 2012-06-14

**Authors:** Michael Marotta, Helen Piontkivska, Hisashi Tanaka

**Affiliations:** 1 Department of Molecular Genetics, Cleveland Clinic Foundation, Cleveland, Ohio, United States of America; 2 Department of Biological Sciences, Kent State University, Kent, Ohio, United States of America; Michigan State University, United States of America

## Abstract

Gene duplication generates extra gene copies in which mutations can accumulate without risking the function of pre-existing genes. Such mutations modify duplicates and contribute to evolutionary novelties. However, the vast majority of duplicates appear to be short-lived and experience duplicate silencing within a few million years. Little is known about the molecular mechanisms leading to these alternative fates. Here we delineate differing molecular trajectories of a relatively recent duplication event between humans and chimpanzees by investigating molecular properties of a single duplicate: DNA sequences, gene expression and promoter activities. The inverted duplication of the *Glutathione S-transferase Theta 2* (*GSTT2)* gene had occurred at least 7 million years ago in the common ancestor of African great apes and is preserved in chimpanzees (*Pan troglodytes*), whereas a deletion polymorphism is prevalent in humans. The alternative fates are associated with expression divergence between these species, and reduced expression in humans is regulated by silencing mutations that have been propagated between duplicates by gene conversion. In contrast, selective constraint preserved duplicate divergence in chimpanzees. The difference in evolutionary processes left a unique DNA footprint in which dying duplicates are significantly more similar to each other (99.4%) than preserved ones. Such molecular trajectories could provide insights for the mechanisms underlying duplicate life and death in extant genomes.

## Introduction

Gene duplication events play a very important role in evolution, as they provide material for genetic innovations [1], [2], [3], [4], [5], [6], [7], [8], [9]. Gene duplication occurs either at the whole-genome level (polyploidization) [10], [11], [12] or at particular genomic segments [13], [14]. Many species living today, such as plants and fish, are descendants from ancient polyploidy ancestors, and thus polyploidization may have significantly contributed to subsequent species diversification. Duplicated genomic segments (segmental duplications, low copy repeats) are particularly abundant in primate genomes. The human genome contains approximately 400 large blocks of recently duplicated regions that exhibit very high sequence identity (>90%) between duplications [13], [15]. Copy-numbers of such duplicated regions are highly polymorphic within humans (copy number variations) [16], [17]. Furthermore, duplicated regions exhibit structural and copy-number divergence between primates [14], [18], [19]. Such divergence could account for more genetic differences between primate species than single-nucleotide substitutions [14], [20] and likely underlie some of the phenotypic differences between primates.

Because of the important role in evolution, several conceptual frameworks have been developed for processes associated with as well as consequences derived from the evolution of duplicate genes [6], [9]. When an allele with gene duplication arises in a population, the allele could achieve fixation either in a neutral fashion (random drift) [21], [22], or by the initial advantage for increased gene dosage [23], [24], [25]. The fixation would result in the existence of two genes with identical functions in the genome. The redundancy of function would relieve a selective constraint for a duplicate pair, thereby allowing mutations to accumulate. Such mutations in most cases are deleterious and lead to the death of duplicate copies [26]. Duplicates that survived would diverge from the original gene and undergo functional innovations: acquisition of new functions (neo-functionalization) or partition of the original function (sub-functionalization) [2], [3], [27]. Several instances have been demonstrated for each outcome [28], [29], [30], [31], [32], illustrating the important role of gene duplications in creating evolutionary novelties.

Although both the significance and consequences of duplicate gene evolution is well established, little is known about molecular mechanisms leading to differing fates. This is mainly because most studies of duplicate evolution focus on outcomes after a very long period of time. There could be critical fate-determining mechanisms for preservation and death at a relatively early stage of duplicate evolution, as most gene duplicates experience nonfunctionalization of one copy, either by pseudogenization or deletion, within several million years [5], [26]. The mechanisms for this early process could be studied for a gene duplication that arose in a common ancestor and experiences alternative fates between two closely related species. In a simple case, we could find two functional copies in one species, while other species have one functional copy, with the other copy being deleted. DNA copy number analyses and sequencing for several individuals in each species could illustrate such a dynamic state of duplicates within a population. Molecular phenotypes, such as gene expression and functional analysis, could help us to further dissect the mechanisms underlying duplicates birth and death, a fundamental process of genome evolution [33].

In this regard, primate genomes are good resources, because recent segmental gene duplications are abundant [14], [18], [20]. Several studies have measured copy number divergence between primates and identified linage-specific deletions of gene duplicates [19], [34], [35], [36], [37], [38], [39], [40], [41]. Here we delineate molecular mechanisms for gene duplicates that have followed alternative fates between humans and chimpanzees. The 29-kb tandem inverted duplication of *Glutathione S-transferase Theta 2* (*GSTT2*) gene, a gene encoding an enzyme for cellular detoxification and thus showing association with cancer [42], [43], [44], [45], is preserved in chimpanzees, whereas a large deletion polymorphism involving one copy of duplicates is very common in humans: 63% in Caucasian, 47% in Yoruban and 50% in Japanese and Chinese (from HapMap Samples) [46]. Thus, *GSTT2* duplicates provide a unique opportunity to study molecular mechanisms associated with the alternative fates of duplicate genes. We found that the mRNA level of *GSTT2* is positively correlated with the preservation of duplication; *GSTT2* is expressed at a much higher level in chimpanzees than in humans. The molecular mechanisms underlying reduced *GSTT2* expression in humans include a repressive regulatory mutation that is propagated between duplicates by gene conversion. Furthermore, DNA sequences of duplicates from several individuals showed that dying duplicates (in humans) are significantly less diverged from each other than preserved ones (in chimpanzees). These molecular footprints suggest evolutionary mechanisms behind the *GSTT2* duplicate preservation and death and offer a novel insight on duplicate evolution.

## Results

### The Origin of Tandem Inverted *GSTT2* Duplication

**Figure 1:**
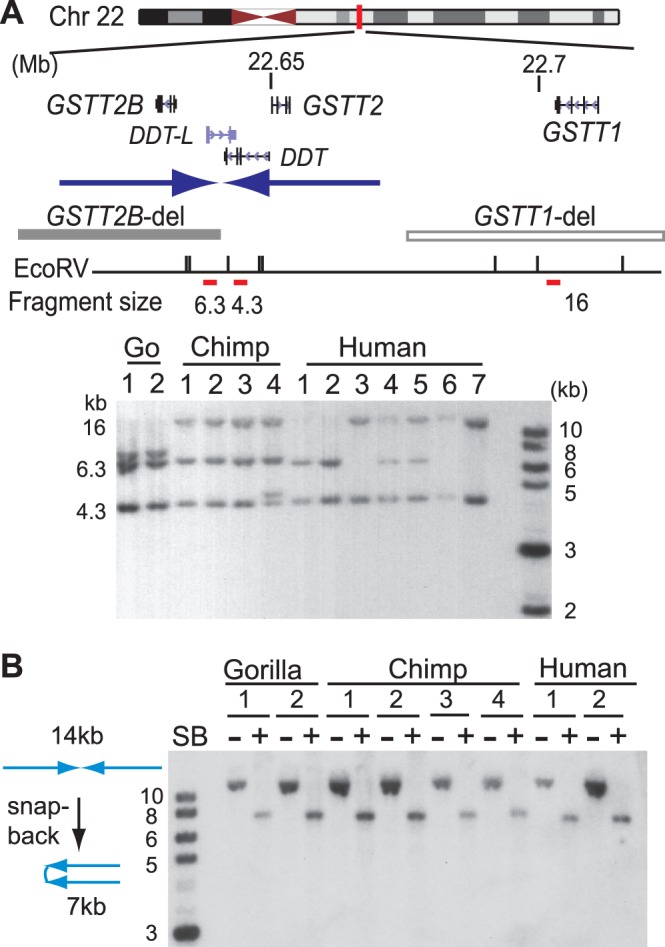
The origin of tandem inverted duplication of *GSTT2.* A. Conservation of the tandem inverted duplication of *GSTT2* gene in humans, chimpanzees and gorillas. The region harboring *GSTT2*, *GSTT1* and *DDT* genes in the human chromosome 22 (top) and Southern analysis for EcoRV-digested genomic DNA from gorilla, chimpanzee and human fibroblasts (bottom) are shown. Inverted duplication is shown as a pair of blue arrows. A red vertical bar indicates the probe for Southern analyses that hybridize to three *EcoRV* restriction fragments. Note that both 6.3-kb and 16-kb fragments are often missing in humans, which corresponds to the deletion of either *GSTT2B* (gray bar) or *GSTT1* (open bar) as previously described [46]. Mb: mega-bases (co-ordinates). B. Southern analysis for either genomic DNA (SB -) or snap-back DNA (SB+) from the fibroblasts analyzed in A. De-naturation and rapid re-naturation (snap-back) facilitates intra-stand annealing of inverted repeat DNA, and the restriction-digest of snap-back DNA results in a half-sized fragment (7-kb).

In the human genome, the 29-kb region including *GSTT2* is duplicated next to the parental gene in an inverted orientation on chromosome 22 (Figure 1A, blue arrows). Due to extensive segmental duplications, the syntenic regions in primate genomes other than humans have many gaps and are not completely assembled. Therefore, to first define the origin of duplication, we applied a Southern blotting-based restriction fragment length polymorphism (RFLP) analysis [46] for primate primary fibroblasts, a tissue that expresses *GSTT2* (Table S1). In human fibroblasts, three *EcoRV* fragments hybridized to a single probe (red bar); one for the repeat harboring *GSTT2B* (6.3-kb), one for the repeat harboring *GSTT2* (4.3-kb) and one for the region near *GSTT1* (16-kb) (Figure 1a, red bars). Consistent with our previous result, a deletion polymorphism involving *GSTT2B* (*GSTT2B*-del), judged by either the lack (Human-3, 6 and 7) or the reduced intensity (Human-4, 5) of a 6.3-kb *EcoRV* fragment, is common in humans. In contrast, all chimpanzee fibroblasts show an equal intensity between 6.3-kb and 4.3-kb fragments. Genotypes were further confirmed by a genotyping PCR assay (Figure S1). Sequences from the boundaries of duplication revealed the perfect alignment between humans and chimpanzees, confirming the common origin of duplication (Figure S2). The two fragments (6.3-kb and 4.3-kb) representing duplication were also identified in gorilla (*Gorilla gorilla*) (Figure 1A, Go). The duplication in gorilla is also tandem inverted, because Southern analysis using snap-back DNA [47] (genomic DNA treated by de-naturation followed by rapid re-naturation) identified a 7-kb *XhoI* fragment (SB +) from a 14-kb genomic fragment (SB −) (Figure 1B).

### Expression Divergence of *GSTT2*

**Figure 2:**
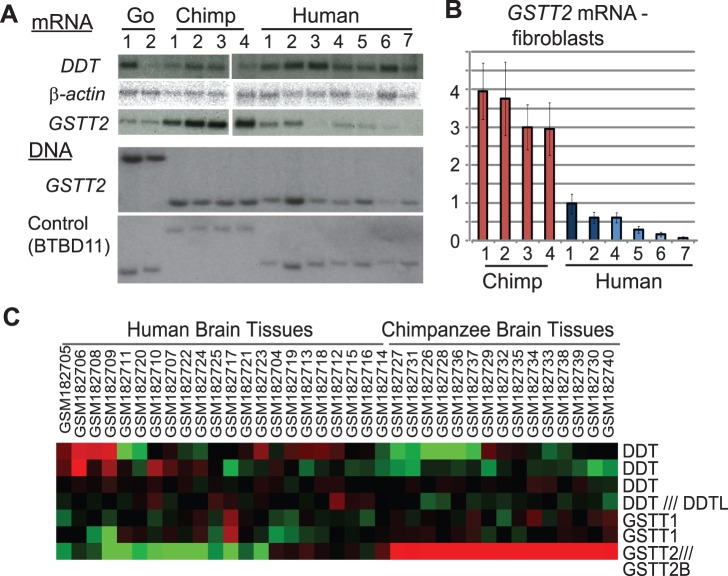
Expression divergence of *GSTT2.* A. Expression divergence of *GSTT2* between primate fibroblasts. Both Northern (mRNA) and Southern (DNA) analysis are shown. cDNA from *GSTT2*, *DDT* and *β-actin* (control) were used as probes for Northern analyses and DNA from *GSTT2* exon 1 and *BTBD11* (control) were used as probes for Southern analyses. B. Real-time PCR based quantification of *GSTT2* mRNA in fibroblasts. Relative expression level (to human fibroblast 1) is shown. The averages of three independent experiments are shown. An error bar represents a standard deviation. C. Expression divergence of *GSTT2* between human and chimpanzee brain. Heat map (red, high and green, low) was generated using Cluster 3.0 from the gene expression profiles from microarray data (Gene Expression Omnibus profiles, GDS2678) [50].

The alternative fates may indicate a differential level of selective constraints for *GSTT2* duplication between humans and chimpanzees. We addressed this issue by examining expression divergence [48], [49]. Indeed, very high-levels of *GSTT2* expression distinguished chimpanzee fibroblasts from human fibroblasts in Northern analyses (Figure 2A). Real-time PCR based quantification indicated that the expression of *GSTT2* mRNA in chimpanzees was at least three-fold higher than that in humans even when fibroblasts homozygous for the duplication allele (chimp 1–4 and human 1–2) were compared (Figure 2B). In contrast, *DDT*, another gene within the inverted duplication (Figure 1A), did not show the expression divergence. The high-level expression is not due to the chimpanzee-specific amplification of *GSTT2*, because the DNA copy number of *GSTT2* is equal between humans and chimpanzees (Figure 2A, bottom). We further investigated the expression divergence using a publically available dataset (Figure 2C). A global gene expression analysis for brain tissues from multiple individuals [50] showed that *GSTT2* mRNA level is consistently higher in all chimpanzees than in humans (Gene Expression Omnibus profiles, GDS2678/1099_s_at/GSTT2). Neither *GSTT1,* the only paralogue of *GSTT2* in both humans and chimpanzees, nor *DDT* showed such differences. These results suggest that the expression divergence of *GSTT2* is not limited to primary fibroblasts, but is also the case in brain tissues.

### Silenced *GSTT2* Duplicates in Humans

The expression divergence associated with the alternative fates between two closely-related species provides a setting to study early molecular trajectories of duplicate evolution. The expression divergence can be due to epigenetic transcriptional regulation, such as the hypermethylation of promoter CpG islands. To test the idea, bisulfate modified DNA was sequenced and methylated cytosines were mapped for the *GSTT2* (and *GSTT2B*) promoter CpG islands (Figure S3). In both humans and chimpanzees, CpG islands were almost methyl-cytosine free, indicating that transcriptional regulation by hypermethylation was unlikely the mechanism. Therefore, we turned our investigation to genetic changes associated with the expression divergence.

**Figure 3:**
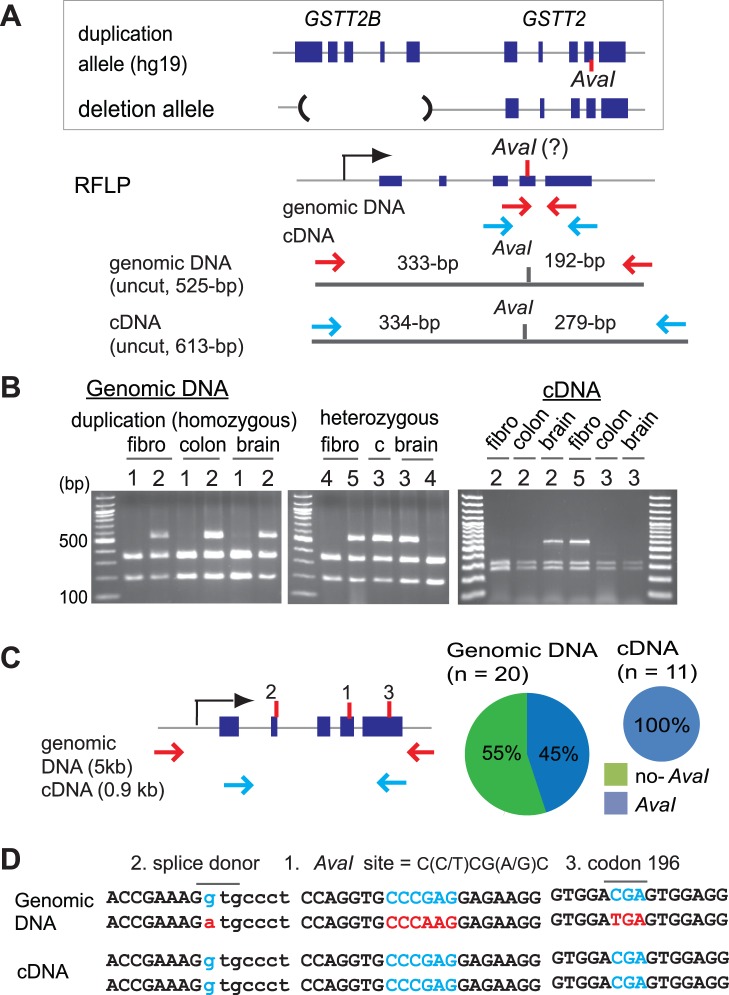
Silenced *GSTT2* duplicates in humans. A. Experimental design for distinguishing GSTT2 from GSTT2B using the paralogous SNP site in the exon 4 of *GSTT2/GSTT2B*. Schematic drawings of both the duplication allele and the deletion allele (top), PCR strategy (middle) and *AvaI* restriction maps for both genomic DNA and cDNA (bottom) are shown. Blue and red arrows represent primers for the PCR. DNA samples are genotyped for the duplicate with an *AvaI* site (333-bp and 192-bp) and the duplicate without an *AvaI* site (525-bp). For cDNA, the duplicate with an *AvaI* site is digested into 334-bp and 279-bp fragments, whereas the duplicate without an *AvaI* site remains as a 613-bp fragment. B. Distinguishing *GSTT2* from *GSTT2B* in several human primary tissues (fibroblasts, colon and brain) (left). The tissue samples harboring the duplicate without an *AvaI* site were examined for its mRNA expression (right). Note that in four tissue samples (fibro-2, Colon-2, colon-3 and brain-3), the PCR product without an *AvaI* site is not seen. c: colon tissue. C. Silenced duplicates confirmed by PCR-clone based single-duplicate sequencing. Results from human fibroblast-2 are shown. For the sequencing, different primer sets (blue arrows) from **B** were used. D. Mutations leading to premature stop codons are associated with silenced duplicates (sequences shown in red). The entire *GSTT2* genomic region was amplified (Figure 3C, red arrows) by PCR and PCR-clones were sequenced. The duplicate without an *AvaI* site (Figure 2c, 1) is associated with a G to A mutation at the consensus sequence of spliced donor of intron 2 (2) and a premature stop codon (CGA to TGA) within exon 5 (3). Both mutations were previously described for Australian individuals [72].

We first determined duplicate-specific expression. This was done by distinguishing *GSTT2* from *GSTT2B* using the paralogous variation (G/A) site represented in the reference human genome (hg19) (Figure 3A). The sequences surrounding a paralogous variation site within exon 4 of *GSTT2* (CCCGAG), but not *GSTT2B* (CCCAAG), is recognized by the restriction enzyme *AvaI.* This allows us to determine expression from each duplicate; a duplicate with an *AvaI* site (*AvaI*-duplicate) and a duplicate without an *AvaI* site (no-*AvaI* duplicate). Genotypes were determined for several human primary tissues (brain, colon and fibroblasts) that were either homozygous or heterozygous for the duplication allele and thus had at least one copy of *GSTT2B* (Figure 3B, left and Figure S1). Out of 11 samples, only 6 showed both an undigested and a digested fragment, indicating that the paralogous G/A site is polymorphic in humans. cDNA from these 6 samples was further examined for duplicate-specific expression (Figure 3B, right). In four samples, we only found fragments digested by *AvaI,* indicating that some of the duplicates without an *AvaI* site are silenced.

We further verified the silenced duplicate by sequencing PCR products (Figure 3C). A single PCR clone sequence represents a sequence from a single duplicate, and a silenced duplicate is seen as a duplicate that is under-represented in PCR clones amplified from cDNA. To rule out the amplification bias introduced by the specific primer set for the *AvaI*-restriction analysis, full-length cDNA was amplified for sequencing (Figure 3C, blue arrows). In fibroblast-2, PCR-clones without an *AvaI* site were exclusively obtained from genomic DNA (11 out of 20 clones), but not from cDNA (0/11 clones) (Figure 3C, right). This under-representation was also the case for primary tissues. The duplicates without an *AvaI* site were also common in PCR clones from genomic DNA for both colon-2 (10/21) and colon-3 (6/14), whereas such fragments were severely underrepresented in clones from cDNA (0/8 and 1/12, respectively, Figure S4).

To determine genetic changes associated with the duplicate-specific silencing, we sequenced long-PCR clones that covered the entire genomic locus of either *GSTT2* or *GSTT2B* (Figure 3C, red arrows and Figure 3D). There are thirty-one sequence changes (eight in coding) in fibro-2, twenty-three (four in coding) in colon-2 and twenty three (five in coding) in colon-3 between *GSTT2* and *GSTT2B* (Table S2). Among the sequence changes, two sequence changes likely affect the stability of mRNA by giving rise to premature stop codons: a mutation at the splice donor site of exon 2 (a consensus GT to AT mutation that would result in aberrant splicing and frame-shift) and a nonsense mutation at codon 196. The silenced duplicate was indeed associated with these two changes, suggesting the important role of these changes in the silencing. Overall, sequence identity between *AvaI* and no-*AvaI* duplicates is extremely high (99.4%, Table S2, Table S4; Genbank accession # JN819426 and JN819427). Therefore, the silencing could have been established by a small number of mutations.

### Propagation of a Regulatory Mutation by Gene Conversion

**Figure 4:**
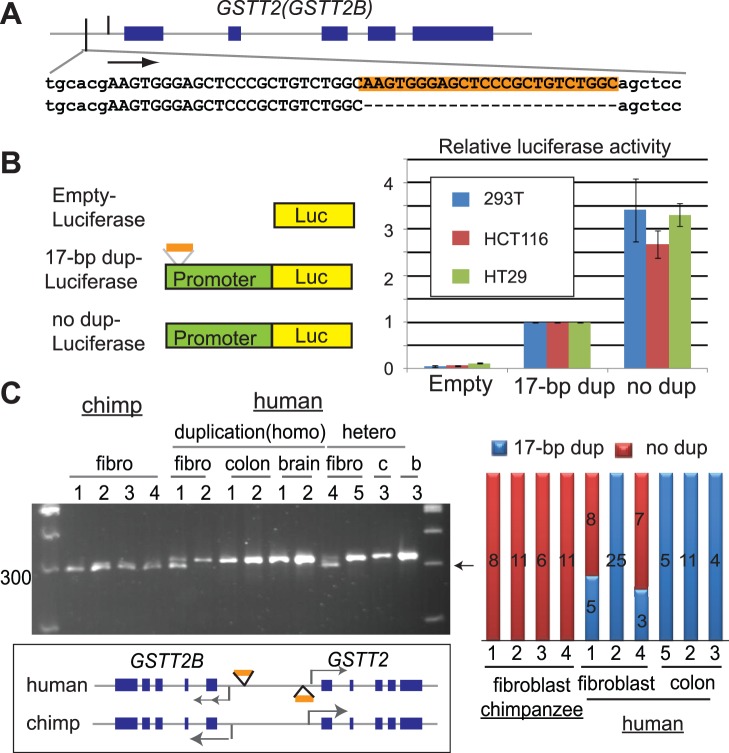
Propagation of a regulatory mutation by gene conversion. A. A 17-bp duplication mutation at the *GSTT2* promoter. Sequences from PCR-clones are shown for the promoter with 17-bp duplication (shaded in orange) and the promoter without 17-bp duplication. B. The 17-bp duplication is a hypomorphic mutation, judged by the reduced luciferase activity associated with the mutation. Either a promoter harboring 17-bp duplication (an orange rectangle) or without the duplication was cloned into pGL3-basic vector (Promega). Each vector was co-transfected with the control vector encoding *Renilla* luciferase into three human cell lines (a human kidney epithelial cell line HEK293T and human colorectal cancer cell lines HCT116 and HT29) for measuring luciferase activity. Relative activities of luciferase (*Firefly/Renilla*) to the activities from 17-bp dup promoters are shown. C. Propagation of the 17-bp duplication in humans. (left) PCR was used to amplify the genomic region containing the 17-bp duplication. Note that most of the human DNA samples show only one PCR product, the duplicate with 17-bp duplication (drawn schematically at the bottom). (right) The numbers of PCR clones for the duplicate without 17-bp duplication (red) and the one with 17-bp duplication (blue) are shown.

The three-fold reduction of *GSTT2* mRNA in humans (Figure 2B) would indicate additional underlying mechanisms for repression. We identified a human-specific, 17-bp duplication in the promoter region (Figure 4A). Such a mutation could function as a regulatory mutation and repress transcription. Indeed, the promoter with the 17-bp duplication (17bp dup-Luc) showed a considerably reduced level of luciferase activity than the promoter without the 17-bp duplication (no dup-Luc) (Figure 4B). In all three cell lines tested, we observed an almost 70% reduction of luciferase activity from the promoter with 17-bp duplication.

Considering the origin of *GSTT2* duplication in the common ancestor between humans, chimpanzees and gorillas, the sequence similarity between duplicates in humans (99.4%) is extremely high. Molecular processes, such as inter-locus gene conversion, could actively homogenize duplicates [33], [51]. Gene conversion could have propagated the regulatory mutation from one duplicate to the other, which could further reduce *GSTT2* mRNA. Indeed, a PCR assay for distinguishing the promoters with 17-bp duplication (322-bp) from the ones without duplication (305-bp) revealed that the majority of human samples only had the promoter with 17-bp duplication (Figure 4C, left). This was further confirmed by sequencing PCR clones (Figure 4C, right). In contrast, chimpanzees only have the promoters without a17-bp duplication. These results illustrate the molecular trajectory of *GSTT2* promoter: (1) the ancestral state is the promoter without a17-bp duplication, as judged by the lack of a 17-bp duplication in chimpanzees (and other primates, Figure S5) and in some humans, and (2) the 17-bp duplication is an acquired mutation in human lineage. The 17-bp duplication observed in both duplicates is unlikely to be anindependent occurrence of thesame exact mutation, but insteadwould likely have been propagated by gene conversion.

### Constraints for the Preserved Duplicates in Chimpanzee

**Figure 5:**
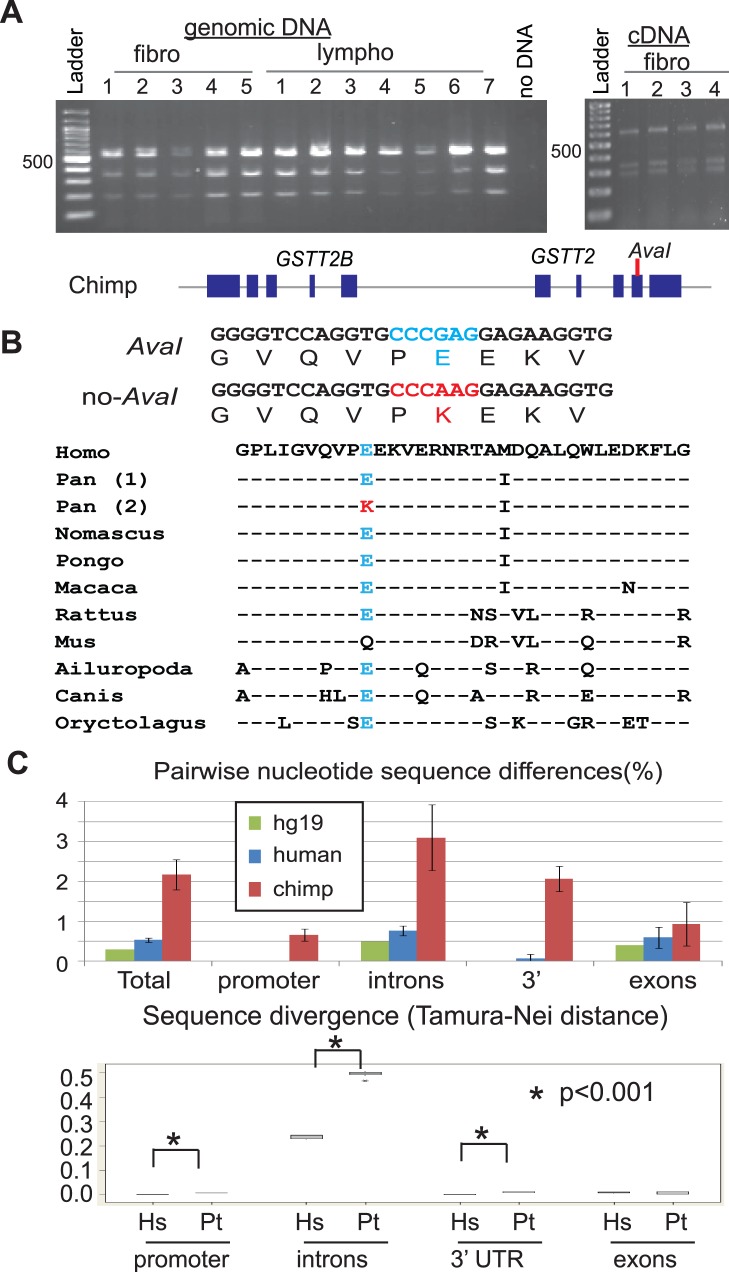
Natural selection for the *GSTT2* duplicates in chimpanzee. A. The paralogous SNP site is preserved and both duplicates are expressed in chimpanzee. *AvaI* RFLP (Figure 2a) shows that three fragments, representing both *AvaI*- and no *AvaI*-duplicates, are present in PCR products amplified from both genomic DNA (five fibroblasts and seven lymphoblasts) and cDNA (four fibroblasts). B. The paralogous SNP at an *AvaI* site is associated with a radical amino-acid change (Glutamate acid to Lysine). The lysine residue is unique to chimpanzees. C. (top) Pairwise nucleotide sequence differences between *AvaI-* and no *AvaI-*duplicates. Average differences in three human samples (fibro-2, colon-2 and -3) and chimpanzees (fibro-2, -3 and -4) are shown along with the differences in the reference human genome sequence (hg19). An error bar represents a standard deviation from an average. (bottom) Average evolutionary distances (Tamura-Nei distance) [71] of coding and non-coding sequences between *AvaI-* and no *AvaI-*duplicates.

Then, how has the duplication been preserved in chimpanzees? High-level expression from the preserved duplicates could indicate either (1) selection for the large amount of *GSTT2* protein, or (2) constraints for two proteins with slightly different function encoded by each duplicate. These possibilities could be distinguished by sequencing duplicates from chimpanzees. As an initial step, we determined the RFLP for the paralogous SNP site in exon 4 (Figure 5A and 3A) that is associated with acidic- to basic-amino acid change (Glutamate acid to Lysine) (Figure 5B). If a large amount of *GSTT2* is solely needed, preserving the paralogous SNP might not be important. Alternatively, if the variation is strictly preserved, both duplicates encoding slightly different proteins could be important. Genomic DNA from 12 unrelated chimpanzees showed that, in all cases, both *AvaI* and no-*AvaI* duplicates were preserved (Figure 5A). Both duplicates are equally expressed (Figure 5A, right) and do not carry mutations leading to premature translation termination. The Glutamate acid encoded by the *AvaI-*duplicate appears to be highly conserved in mammals (Figure 5B), suggesting that the substituted Lysine might have a unique and important function for chimpanzees.

The paralogous SNP observed in both chimpanzees and some humans indicates its origin in the common ancestor’s genome. Selective constraints might have actively preserved such divergence in chimpanzee, whereas gene conversion eliminated the paralogous SNP in some of the human duplicates (Figure 3B, fibro-1, 4, colon-1 and brain 1, 4). Although demonstrating evolutionary constraints in such highly similar sequences is a challenge [52], the patterns of sequence divergence between *AvaI*- and no-*AvaI* duplicates might imply such a scenario (Table S4; GneBank Accession # JN819426, JN819427, JN819428, JN819429, JN819430, JN819431, JN819432, JN819433, JN819434, JN819435, Jn819436 and JN819437). First, analysis of duplicates from three unrelated individuals of each species indicated that nucleotide sequences are appreciably less similar in chimpanzees than in humans (Figure 5C, top). Second, estimating divergence in coding and non-coding regions separately revealed that such a difference is significant only in non-coding regions (Figure 5C). A phylogram showed that, although exons are similarly related to each other in both species, introns are more diverged in chimpanzees than in humans (Figure S6). These patterns imply that *GSTT2* duplicates might have been under selective constraint, such as purifying selection, in chimpanzees. Intron divergence in chimpanzees is estimated to be 3%, thus, greater than the average sequence divergence between humans and chimpanzees (1.23%) [52], which is consistent with the occurrence of *GSTT2* duplication in the common ancestor of humans, chimpanzees and gorillas.

## Discussion

**Figure 6:**
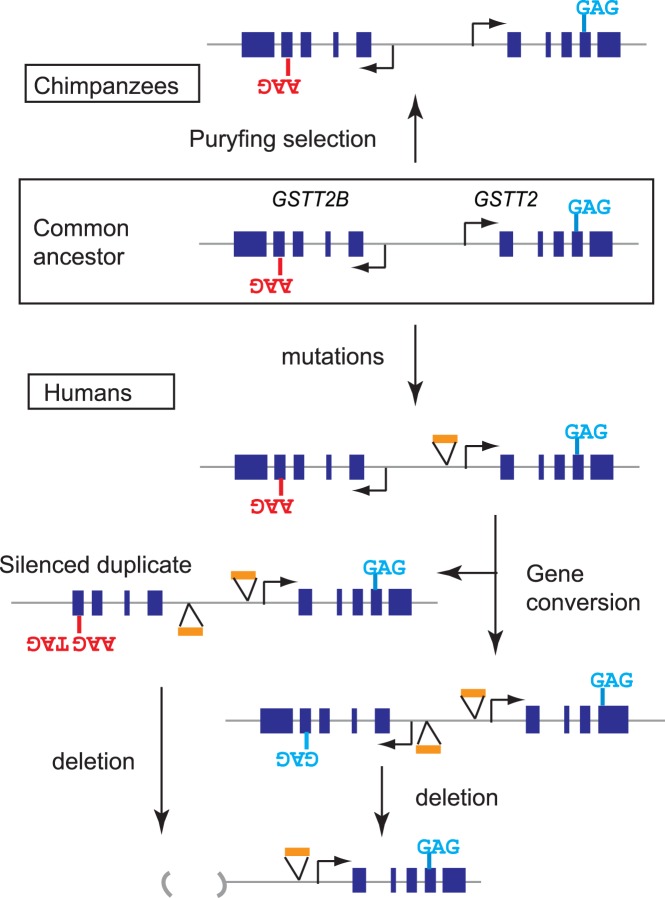
Molecular trajectories for the evolution of *GSTT2* duplication. Purifying selection could have maintained the paralogous SNP site in chimpanzee. Under relaxed selection (for human duplication), gene conversion have homogenized duplicates, which resulted in (1) erasing the paralogous SNP and (2) transferring hypomorphic mutations.

In this study, we delineated evolutionary processes that differed between preserved and dying *GSTT2 (Glutathione S-transferase Theta 2)* duplicates, and elucidated important molecular events for each trajectory (Figure 6). First, we traced back the origin of tandem inverted *GSTT2* duplication to the common ancestor of African great apes (Figure 1). The level of *GSTT2* mRNA expression distinguishes dying duplicates (in humans) from preserved ones (in chimpanzees) (Figure 2). The mutations introducing premature stop codons are associated with a silenced duplicate (Figure 3), and regulatory mutations have been propagated between duplicates by gene conversion (Figure 4). In contrast, natural selection could be responsible for the preservation of duplicates and high-level expression in chimpanzees (Figure 5). These processes have left a paradoxical molecular footprint for duplicate evolution; DNA sequences are more similar to each other in dying duplicates than in preserved duplicates (Figure 5 and S6).

In the case of *GSTT2* duplication, observed mRNA levels distinguished preserved duplicates from dying ones. The expression divergence was not only seen in primary fibroblasts but also found in brain tissues (Figure 2). In the study conducted by Caceres et al., *GSTT2* was one of the most differentially expressed genes between human and chimpanzee brain [50]. Thus, GSTT2 (and a paralogue GSTT2B) likely has a more important function in chimpanzees than in humans. GSTs (Alpha, Mu, Pi and Theta) are a group of phase II enzymes for cellular detoxification and solubilize harmful molecules by conjugating a hydrophilic tag (GSH) to the molecule. Among GSTs, theta class (GSTT) exhibits several distinct properties from other GSTs. First, theta is the most ancient class and is highly conserved from bacteria to mammals [42], [43], [53]. Other classes are considered to be derived from the theta class by gene duplication. Second, unlike other GSTs that have evolved to combat a broad spectrum of toxins and thus are predominantly expressed in liver and kidney [54], GSTT2 is ubiquitously expressed. Furthermore, GSTT2 has distinct structural features in both the active catalytic site and the C-terminus region that defines substrate repertoire from other GSTs [55]. The highly conserved Tyr residue, a critical residue for activating GSH binding in other classes, is replaced by Ser. The C-terminal extension in the theta-class proteins completely buries the substrate-binding pocket and creates the least accessible substrate binding sites among GSTs, indicating a narrow target specificity of GSTT2. These unique properties lead to the notion that GSTT2 is not a typical enzyme for eliminating wide range of exogenous toxins, but may protect cells from endogenous harmful molecules generated by oxidative stresses [55], [56]. The distinctive fate and expression divergence of such a gene could be important to consider for understanding phenotypic differences between two species. One possibility is that a redundant function of another theta class enzyme GSTT1 compensates the low level of GSTT2 expression in humans. However, this is unlikely, because the intra-class similarity in theta class is very low, and only 55% of amino-acids are conserved between GSTT1 and GSTT2 [42]. Furthermore, we did not observe an over-expression of GSTT1 in humans (Figure 2B and data not shown).

Three mutations (a 17-bp duplication mutation, a mutation at the spliced donor site of intron 2 and a premature stop codon) collectively silence a particular duplicate. A 17-bp duplication within the promoter greatly reduced *GSTT2* mRNA in our promoter assay (Figure 4B). On the other hand, the 17-bp duplication is prevalent in human duplicates, many of which express low levels of *GSTT2* mRNA. Therefore, the 17-bp duplication itself is not sufficient for the silencing. We further investigated the involvement of mRNA degradation process as a potential mechanism. A mutation at the splice donor site of intron 2 could cause an aberrant splicing that results in a frame-shift and a premature termination codon. A premature termination codon could induce an mRNA degradation process called nonsense mediated mRNA decay (NMD) [57], [58]. NMD is a conserved cellular process that surveys premature termination codons and prevents the expression of truncated proteins. NMD is triggered by a protein complex that is located within exon-exon junctions of mRNA (Exon junction complex, EJC). Termination codons that are located upstream of EJC can be recognized as “premature”, because authentic termination codons should be located at the downstream of last exon-exon junctions. (In this regard, the nonsense mutation located at the final exon, codon 196 in Figure 4D, will not trigger NMD.) We rigorously investigated the involvement of NMD in the silencing of a *GSTT2* duplicate by commonly used methods: inhibiting either a component of NMD (Upf1) by shRNA or translation using cyclohexamide. In either case, we were not able to detect the expression of an aberrantly spliced product (data not shown). Therefore, NMD is unlikely to contribute to the observed complete silencing. Other possible changes, such as a regulatory mutation further upstream of the promoter, could co-operate with the 17-bp duplication for the silencing.

We defined differential molecular trajectories between preserved and dying *GSTT2* duplicates. Although it remains to be determined how common these molecular trajectories are in the aging processes of gene duplicates, the trajectories offer important insights into molecular mechanisms underlying duplicate evolution. Several studies have shown that very young duplicates are over-abundant across the genomes of eukaryotes [5], [59], [60], [61], [62]. In addition to the accelerated recent duplication activities [14], [20], the rejection of molecular clock by gene conversion could also result in the over-abundance of dying, older duplicates that have very high sequence similarities. Older duplicates could look young when gene conversion homogenizes duplicates. However, such conversion could also propagate deleterious mutations [63], [64], [65] as we see for *GSTT2* duplicates, and would put the lives of both duplicates in danger. An allele with the deletion of one duplicate could rescue the function of pre-existing genes, because a deletion could prevent such unwanted gene conversion. Therefore, some of the duplicates with very high-sequence similarities may not indeed be young, but can instead be older and would soon be eliminated from the genomes.

We identified a silenced duplicate at an early stage of duplicate evolution. Silenced duplicates have long been conceived as an intermediate leading to a new gene function due to its neutrality for mutational effects [1], [66], [67], [68] (Figure S7). Mutations accumulating in duplicates may not be completely neutral, because the function of gene products depends on other gene products and environmental conditions [69], [70]. For example, gene products very often function in protein complexes and hence mutant proteins could compete with wild-type ones to participate in complexes. Mutations in silenced duplicates can be ignored by natural selection, because silenced duplicates are untranslatable. However, silenced duplicates need to regain expression. Two findings from our study suggest a mechanism. First, silenced duplicates can be established by a relatively small number of mutations. Second, gene conversion occurs in dying duplicates and can modify sequences for silenced duplicates. A typical short conversion tract could be sufficient to erase silencing mutations.

By defining duplicate-specific sequences from several individuals, we were able to determine the dynamic state of *GSTT2* duplicate evolution within a population. In humans, *GSTT2B* is either a functional gene (as represented in hg19), a silenced pseudo-gene or a deleted gene. In contrast, *GSTT2* maintains a paralogous SNP in chimpanzees. Therefore, in addition to the status of copy number variations, genomic sequence information is necessary to elucidate the evolutionary history of duplicate genes. This is also important for testing gene-disease associations, as defining the functional state of each copy is essential for accurately measuring disease associations.

## Materials and Methods

### DNA and RNA Manipulations

All the duplicate sequences (Table S4) were deposited to NCBI (GneBank Accession # JN819426, JN819427, JN819428, JN819429, JN819430, JN819431, JN819432, JN819433, JN819434, JN819435, Jn819436 and JN819437).

Details of primary tissues used for this study are described in Table S1. Primary fibroblasts from gorillas, chimpanzees and humans were obtained from Coriell Institute (http://www.coriell.org/). Commercially available normal colon DNA was purchased from Biochain (www.biochain.com). Primary brain tissues were obtained from the Cleveland Clinic Human Biospecimen Resource. Research using specimens obtained from the Cleveland Clinic Human Biospecimen Resource falls under the category of “human specimen research that does not involve human subjects” and is not regulated by 45 CFR Part 46.

PCR primers for cloning DNA fragments, for PCR-RFLP analyses, for sequencing and for quantitative Real-Time PCR are listed in Table S3. DNA and RNA extractions, Southern and Northern hybridizations, Real-time PCR were performed as described previously [46]. For generating snap-back DNA, *XhoI*-digested genomic DNA was denatured by boiling for 7 minutes in the presence of 100 mM NaCl, followed by rapid re-naturation on ice for 10 minutes [47].

### Long-PCR and Sequence Analyses

The genomic sequence from each duplicate was obtained by long-PCR and PCR-clone based sequencing. The genomic regions covering entire *GSTT2/GSTT2B* genes were amplified using two different primer sets (Table S3) to eliminate amplification bias from a single primer set. Human fibroblast DNA was amplified using FastStart High Fidelity TAQ Polymerase (Roche). PCR products were cloned into pSC-A (Stratgene). Chimpanzee fibroblast DNA was amplified using Phusion Hot Start Pol II (New England Biolabs) and cloned into pSC-B (Stratagene). PCR clones were isolated using the Fast Plasmid Miniprep Kit (Five Prime) and the entire 5 kb region was sequenced using six different primers. All of the sequences were compiled using the DNAStar Lasergene 8 programs, and sequence divergence between *GSTT2* (*AvaI*-duplicate) and *GSTT2B* (no-*AvaI* duplicate) was calculated using NCBI Blast Alignment.

The extent of evolutionary divergence between nucleotide sequences was calculated using MEGA5 (http://www.megasoftware.net/) [71], using Tamura-Nei substitution model that takes into account GC content biases as well as unequal rates of transition and transversion. To compute evolutionary distances, any site at which the alignment postulated a gap (indels) was removed from all comparisons so that a comparable set of sites was used for each comparison. Statistical analyses were done using Minitab 15 (http://www.minitab.com/en-US/default.aspx).

### Transcriptional Activity of *GSTT2*/*GSTT2B* Promoters

The promoter for *GSTT2*/*GSTT2B* was PCR-amplified and cloned from human fibroblast-1, a fibroblast that have both GSTT2 and 17-bp dup promoter (Figure 3a). The promoter region amplified was either a 1,054-bp (GSTT2) or a 1,071-bp fragment (17-bp dup) that corresponded to the sequence up to 1 kb upstream from the translation initiation site. The PCR products were verified by sequencing prior to being cloned into the upstream of a firefly luciferase gene (pGL3-Basic vector, Promega). For the luciferase assay, each luciferase construct was co-transfected with a control construct (encoding *Renilla* luciferase) into 1.6×10^5^ cells. At 48 h post-transfection, the amount of both firefly luciferase and Renilla luciferase was measured using a WallacVictor^3^ luminometer.

## Supporting Information

Figure S1 *GSTT2* duplication/deletion genotypes of human primary tissues (p. 2).
(EPS)

Figure S2 Sequence conservation of the boundaries of duplication between humans and chimpanzees (p. 2).
(EPS)

Figure S3 Maps of methylated cytosines for the *GSTT2/GSTT2B* promoter CpG islands (p. 3).
(EPS)

Figure S4 Silenced duplicates in the primary colon tissues (p. 4).
(EPS)

Figure S5 A 17-bp duplication is specific to human lineage (p. 4).
(EPS)

Figure S6 Neighbor joining tree of *GSTT2* exons (top) and introns (bottom) based on maximum composite likelihood (p. 5).
(PDF)

Figure S7 Silenced duplicate-mediated gene innovation (model) (p. 6).
(EPS)

Table S1 Primary tissue samples (p. 7).
(XLS)

Table S2 Sequence identity between *AvaI*- and no *AvaI*- duplicate (p. 8).
(XLSX)

Table S3 Primer list (p. 9).
(XLS)

Table S4 Sequences of AvaI- and no AvaI-duplicates (human and chimpanzee) (p. 10–37).
(DOC)
