# Supplementary material for: Molecular Trajectories Leading to the Alternative Fates of Duplicate Genes
Source: PLoS One. 2012 Jun 14;7(6):e38958. doi: 10.1371/journal.pone.0038958 (PMC3375281; doi:10.1371/journal.pone.0038958)
Supplement: Figure S6 — Neighbor joining tree of GSTT2 exons (top) and introns (bottom) based on maximum composite likelihood (p. 5). (PDF) [file pone.0038958.s006.pdf]

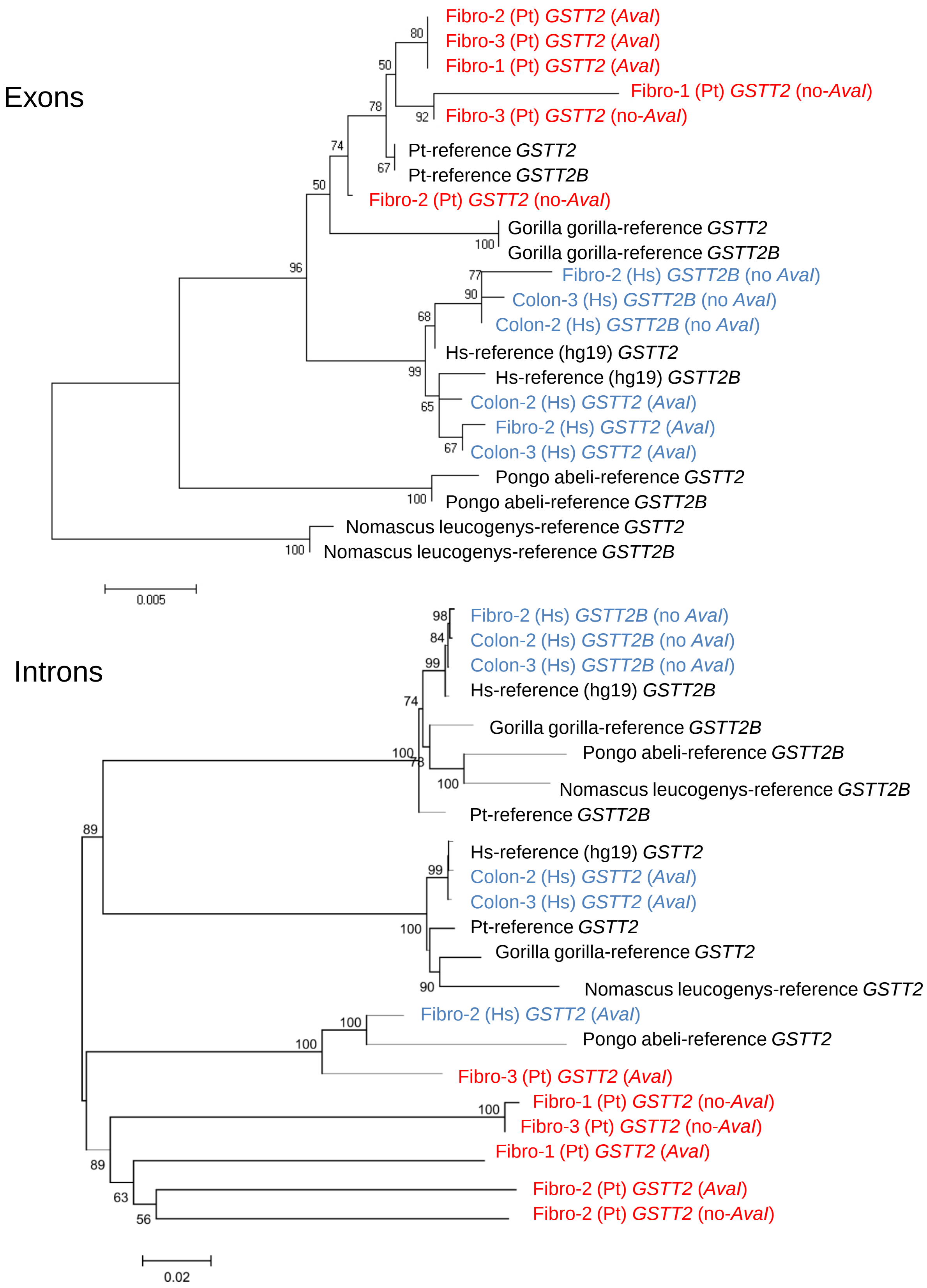

Figure S6. Neighbor joining tree of *GSTT2* exons (top) and introns (bottom) based on maximum composite likelihood. Numbers on branches represent confidence levels of the standard error test of branch length; only values >50% are shown. Note that exons are less diverged than introns. The sequences obtained from this study are shown in red (chimpanzees) and in blue (humans).
