# Supplementary material for: Molecular Trajectories Leading to the Alternative Fates of Duplicate Genes
Source: PLoS One. 2012 Jun 14;7(6):e38958. doi: 10.1371/journal.pone.0038958 (PMC3375281; doi:10.1371/journal.pone.0038958)
Supplement: Table S4 — Sequences of AvaI- and no AvaI-duplicates (human and chimpanzee) (p. 10–37). (DOC) [file pone.0038958.s011.doc]

**Table S4.** Sequences of *AvaI*- and *no AvaI*- duplicates (human and chimpanzee)

**Accession No. JN819426 - Human Fibroblast-2 (AVA)**

>Promoter

CCGTGAGAGTAACTGGGATTGCAGGTGTGGGCCAGCATGCCCTGTTATCACTCCCTTTCTCTGAGTCAGCTTTTCTCTCTCAGTCTCGTCTACTCCTCGAACTCAGGAAATGTCACTACAGGTGGCCCCAGCTCCCGTTTACCTCCCCATAGGGAGCTCCTCTCTCCAGTTCCAGTTTCAAAACTCCCAAGGAAGCATTCTGGTTCACTCGCTTGGGCCACTGGCCAGAGGGATGGGATACTCTGAAAGATTCAGCTAGAGTCCCGGGCCCAGCCCTGGACCATCACTGTGCCCCCTGGTGAGATGCCAGGGCTGGGATTCAGGGAGAAGAAAGGAGGTTCCCGGACAGTCATTCCTGCCTCCCGCGGCTGCGGGCTCCCTGCCCCCATCCTGTGCACGAAGTGGGAGCTCCCGCTGTCTGGCAGCTCCCGCTGTCTGGCAGCAGCTGCTCTGCAGGGGACAGTCTGGACGGCAGAAAGTTCATCCTTAACCCCAGCCTTCCAGTCAAGGTTCCCACCAGTTTGGGACACCTGCAAATGTCACATCCCACTGGGTGAAACTCTAAGATCCCTTTTAGGGGATCCCATTCGCTCCCTCCCTTCCGCCACCATGCAGCGCCGAGAAACAGAGCTCTGAACGAACCCTCAGATGTCCGTGCGCTGGGGCCTTTCCAGGACGGCGGCGCCCAGTCGTTTCTGGGTCAGGGCGACGCCTGGAACTGGGCAGGGTCCCTGGCACCGGGATCCCGAAAAGCAGACCTGCTTCTCCCTGTCCAGCCGGTTCCCCTTCCCCTTGCAGTCGGCCCCCTGCATCCGCGTCCTCCCTGCCAGTCGAGGGTCCCCAGCTCCAACTCCACCCTCCCAGCTGTGCGTTCATAGCGACCGCCCTCCCTGTAGGGACGCACGGATCTGGTGGTGGAGTCTTGGCCGGCAGGACTGGACAGGAACCGAAGGGGCGAGGCGGGTCCGGGGGTGGTGCGCTCCAATTGGGTGCTGTCCCCAGGGGGTGGGGCCTGATCCCCTATTTCCCGGCGCGCCGG

>Exon 1

GATCCTGCCACAGCTGCTGCCCACACCGCGCTCAGCGCCTTCACTGCCATCCCCGCTGTCCTTGCCGCCCCCGCCATGGGCCTAGAGCTGTTTCTTGACCTGGTGTCCCAGCCCAGCCGCGCCGTCTACATCTCCGCCAAGAAGAATGGCATCCCCTTAGAGCTGCGCACCGTGGATTTGGTCAAAG

>Intron 1

GTGGGCCCAGCCCGTTTCCCCGCGTGTCCACAAACCCAGTGCACCCCCAGGCCCCCGCCCTGCTCTGCCCTGAGCGTCTCGCCGCCCGCACAGCCCCCTCACCTCCTCCTGCAGCGTCTGCCACCAGAGAATGCTGTGGACTGAGTGGCCTTGGAGGGATCACAGCCTCTCTGAACCTTAGCTTGCCTTCTAAAAAGGAGGATAACGTTACCTTCTGCTCTGTAGGGATGGAAAGAAAATACTGAATGGAGTTGACAGAGTTCTTGCGTGGAATGCACGCATATAAATTCACAAAGCCCAGAAGACCTCGGGAAGAAGGACATGCTGTTGTGAGAATTAAGAGATGGGAAGAGATGAGCCACCCCAGTTTGCCTCCCCTCCCCTGGCCCACCAGAGTCCGGCTAGAAAACTTCTCTTTATCCACCTGCTGCACCTGGCCCCACCCACCAAAGCCCCCCAGCTGCCCCGGAATGTGGCAGGGCAGGGAGGCCCAGCCAGGGAGTGAGGCTGATCCAGGCCTCTAGTCCCAGACCTTGCTGTTTCTCAGGGCTGTGGGGCTCCGCTTGGGGAGGAGGAGGGAGGGTGTAGAGGTGCAGCGTTTTTACTCTGAAGACCTTTTCTGACTTCTTCCTCTTCAG

>Exon 2

GGCAGCACAAGAGCAAGGAGTTCTTGCAGATCAACAGCCTGGGGAAACTGCCGACGCTCAAGGATGGTGATTTCATCTTGACCGAAAG

>Intron 2

GTGCCCTCCTTCCCTCACCCCTCACCGCATCCGGAGCCCATGTGACCTTGGCTCTCCCCACTGGCCCCGGGCCCCAGTGGCCCTCCCATACCCCATGGGGCAGCGAGGGAGGGGAAAGGCGAGGGATCTGGCCGGGCGCGGTGGCTCACGCCTGTCATCCCGGCACTTCGGGAGGCCAAGGCGGGCGGATCACGAGGTCAGGAGACCGAGACCATCCTGGCTAACACCGTGAAACACCGTCTCTACTAAAAATGGAAAAAAAGTTAACCGGGCGTGGTGGCGGGCGTCTGTAGTCCCAGCTACTCTGGAGGCTGAGGCAGGAGAATGGTGTGAACCCAGGAAGCGGAGCTTGCAGTGTGCTGAGATCGCGCCACTGCACTCCAGCCTGGGCGACAGAGCTAGACTCCGTCTCAAAAAGGCCGGGCGCGGTGGTTTACGCCTGTAATCCCAGCACTTTGGGAGGCCGAGGTGGGCGGATCACGAGGTCAGGAGATCGAGACCATCCTGGCTAACACGGTGAAACCCTGTCTCTACTAAAAATACAAAACATTAGCAGGGCGTGGTGGCGGCCGCCTGTAGTCCCAGCTACTGGGGAGGCTGAGGCAGGAGAATGGCGTGAACCCAGGAGGCGGAGCTTGCAATGAGCAGAGATAGCGCCACTGCACTCCAGCCTGGGTGACAGAGGGAGCCCACTCCAGCCTGGGCGACAGAGGGAGACTCCGTCTCAAAAAAAAGGAAAGAAAGAAAGGAGAGGTATCTGGGGAGAAGGTACAGCTTGGGGTGTGACCGGGATGAGCAGGGGCTGACAGAACATGTCCCCCCACCTCTCATCTTCCAGCCTTTTCTGAGCCGCAGGGCCTCTCCACTCCCAGACTGAAGGGTATTAGAAGAGAAGACAAGGGAACATTTTTCCACTGTTGCGCATTTGTTCAACAAATGCTAGCTGAAAAGAGCCTCTAGTGACTTGTCGCAGACTACCCAATCTACCCAGGCCGGGCCTAGAGGCCAATGCCATGGCCCAAAGGCACAGCTCATGGTGAGGTCCAGCTGCTGGGCAGGAAAAGGACAAGAGGTCAGGTGGCTGCAGAGGTGATGGCTGGGGGCCTGTCAGACGGGGGCCAAAGACATTCCTCCCCTCGTGATCCCTGACCCAAGCGCGTGGACATGCAAGGGACTCCACGGAGCATCCACTGTGTGCCAGCCCCATGCAGGGTTCCAGGGGTCCAGGGAGCCTATTCTGAGCTGCACCGCCTCGGACAAGTCACTTGACCATTCTGACCTTGAGTTTTCTCTTGTGCTAAAAGGCTAACAGGAGTGTCTACCTCACAGGGCGGCTGCTGGCATATCACGGAGATGAGGTTCTCAAAATGCAAAGCAGAAGGTCCAGCCAAGAGTCGGTGCCCAAGGCAACAAAGACAGGAGGAGACTCGTAGGAGGAGGGGGTGGTGTTGGGGAGCTGGAGATGGAGGGCGAGGCTGGAGGGCAGTCCTTCAAATGCAGAGAAGCCCCCGGGCCCCACTGGCAGATGGGAGCAGTTAGGGGTAAATGCCTGGTGCCAGTGTCCTTATAGCCACTGCCCATTTGTTCCCAG

>Exon 3

CTCGGCCATCCTGATTTACCTGAGCTGTAAGTACCAGACGCCGGACCACTGGTATCCATCTGACCTGCAGGCTCGTGCCCGTGTTCATGAGTACCTGGGCTGGCATGCCGACTGCATCCGTGGCACCTTTGGTATACCCCTGTGGGTCCAG

>Intron 3

GTGAGGAGAGCCATCTGGAGAGTGATTGGCCATCAGAGAGTAGTTGGCAGTAGGTCGGGGCCATAGACTGACCCACTGTCTGCCCCCATCAG

>Exon 4

GTGTTGGGGCCACTCATTGGGGTCCAGGTGCCCGAGGAGAAGGTGGAACGCAACAGGACTGCCATGGACCAGGCCCTGCAATGGCTGGAGGACAAGTTCCTGGGGGACAGGCCCTTCCTCGCTGGCCAGCAGGTGACACTGGCTGATCTCATGGCCCTGGAGGAGCTGATGCAG

>Intron 4

GTGTGAGCTCAGCCTGTGGGCAGTGTCCCTCTTCGTGTCACACCCATGAGGCAGACAGAAACACTGAGGCCTGGAGAAAGCCAGAACTTTGCCCAGAATCATAGAGCAAGTCTCTGGATGATCTGGGGCCAGAACCCTGAACTTCTGCCTCCTGCCTGGGTGTGGGGTCTCACCCTGGCTGCTCTTGGGCTCTAAGGCTGAACATACTGCCTGGGCCCCTGTGGTCCATTCACTTAGGGGCTGGGGAATGGACCATGTCTCTGATACTTCTGCCCATGGTTCCAGCATTCGGGTCGGCAGTGACAACTGGGAAAGTTGTATGCCCACAACTTTTTCATCCTTGTCCCTACAG

>Exon 5

CCGGTGGCTCTCGGCTACGAACTGTTTGAGGGACGGCCACGACTGGCAGCATGGCGTGGACGAGTGGAGGCTTTCCTGGGTGCTGAGCTATGCCAGGAGGCCCACAGCATCATCTTGAGCATCCTGGAACAGGCGGCCAAGAAAACCCTCCCAACACCCTCACCAGAGGCCTATCAGGCTATGCTGCTTCGAATCGCCAGGATCCCCTGA

>Post

AGGGTCTGGGATGGGGGCCAGGAGATTAGCAACAAGGATTCATTCTGTTACTTACTTGCCCCTTTTTATCTTTCCCTCTTGCCCCAGTCCCTTCTCTCCAGCTTCATGTGAAGCTCTGCACAGACAAGACACTCAGTGTCCTTGGCAGTGCTGCTACTCCTCAGGTGCAGCATACATAACCAGTAAGAGACTAAATCTGCAATATATAAAGAGCTCCTACAAATCAGTAACATGAAGAACACTCAAAAATTGGCAAATGTCATCAGTGTTTTAAACAGAATAAAGATTCCAAACACTTTGAATAGAGAACCAAGAGTTATTGGTTTTACTACATTGTTGTGTTATACATATGGAGTAAAAGTATGTGCTAGTAATCCTCATCATGGTTAATAACAAAGTAACCTCACAATAACGAGTCAACATAATTGTATCACCAGGGCAACAAAATGTTAAGTAAGTAACCAATTCGAATTGCAAACTGTTAAAGGATATAGGCGATGTTTCACAGGGCATAGCAACGGTCTTTGAAGTCTAGGAA

**Accession No. JN819427 - Human Fibroblast-2 (NO AVA)**

>Promoter

CCGTGAGAGTAACTGGGATTGCAGGTGTGGGCCAGCATGCCCTGTTATCACTCCCTTTCTCTGAGTCAGCTTTTCTCTCTCAGTCTCGTCTACTCCTCGAACTCAGGAAATGTCACTACAGGTGGCCCCAGCTCCCGTTTACCTCCCCATAGGGAGCTCCTCTCTCCAGTTCCAGTTTCAAAACTCCCAAGGAAGCATTCTGGTTCACTCGCTTGGGCCACTGGCCAGAGGGATGGGATACTCTGAAAGATTCAGCTAGAGTCCCGGGCCCAGCCCTGGACCATCACTGTGCCCCCTGGTGAGATGCCAGGGCTGGGATTCAGGGAGAAGAAAGGAGGTTCCCGGACAGTCATTCCTGCCTCCCGCGGCTGCGGGCTCCCTGCCCCCATCCTGTGCACGAAGTGGGAGCTCCCGCTGTCTGGCAGCTCCCGCTGTCTGGCAGCAGCTGCTCTGCAGGGGACAGTCTGGACGGCAGAAAGTTCATCCTTAACCCCAGCCTTCCAGTCAAGGTTCCCACCAGTTTGGGACACCTGCAAGTGTCACATCCCACTGGGTGAAACTCTAAGATCCCTTTTAGGGGATCCCATTCGCTCCCTCCCTTCCGCCACCATGCAGCGCCGAGAAACAGAGCTCTGAACGAACCCTCAGATGTCCGTGCGCTGGGGCCTTTCCAGGACGGCGGCGCCCAGTCGTTTCTGGGTCAGGGCGACGCCTGGAACTGGGCAGGGTCCCTGGCACCGGGATCCCGAAAAGCAGACCTGCTTCTCCCTGTCCAGCCGGTTCCCCTTCCCCTTGCAGTCGGCCCCCTGCATCCGCGTCCTCCCTGCCAGTCGAGGGTCCCCAGCTCCAACTCCACCCTCCCAGCTGTGCGTTCATAGCGACCGCCCTCCCCGTAGGGACGCACGGATCTGGTGGTGGAGTCTTGGCCGGCAGGACTGGACAGGAACCGAAGGGGCGAGGCGGGTCCGGGGGTGGTGCGCTCCAATTGGGTGCTGTCCCCAGGGGGTGGGGCCTGATCCCCTATTTCCCGGCGCGCCGG

>Exon 1

GATCCTGCCACAGCTGCTGCCCACACCGCGCTCAGCGCCTTCACTGCCATCCCCGCTGTCCTTGCCGCCCCCGCCATGGGCCTAGAGCTGTTTCTTGACCTGGTGTCCCAGCCCAGCCGCGCCGTCTACATCTTCGCCAAGAAGAATGGCATCCCCTTAGAGCTGCGCACCGTGGATTTGGTCAAAG

>Intron 1

GTGGGCCCAGCCCGTTTCCCCGCGTGTCCACAAACCCAGTGCACCCCCAGGCCCCCGCCCTGCTCTGCCCTGAGCGTCTCGCCGCCCGCACAGCCCCCTCACCTCCTCCTGCAGCGTCTGCCACCAGAGAATGCTGTGGACTGAGTGGCCTTGGAGGGATCACAGCCTCTCTGAACCTTAGCTTGCCTTCTGAAAAGGAGGATAATGTTACCTTCTGCTCTGTAGGGATGGAAAGAAAATACTGAATGGAGTTGACAGAGTTCTTGCGTGGAATGCACGCATATAAATTCACAAAGCCCAGAAGACCTCGGGAAGAAGGACATACTGTTGTGAGAATTAAGAGATGGGAAGAGATGAGCCACCCCAGTTTGCCTCCCCTCCCCTGGCCCACCAGAGTCCGGCTAGAAAACTTCTCTTTATCCACCTGCTGCACCTGGCCCCACCCACCAAAACCCCCCAGCTGCCCCGGAATGTGGCAGGGCAGGGAGGCCCAGCCGGGGAGTGAGGCTGATCCAGGCCTCTAGTCCCAGACCTTGCTGTTTCTCAGGGCTGTGGGGCTCCGCTTGGGGAGGAGGAGGGAGGGTGTAGAGGTGCAGCGTTTTTACTCTGAAGACCTTTTCTGACTTCTTCTTCTTCAG

>Exon 2

GGCAGCACAAGAGCAAGGAGTTCTTGCAGATCAACAGCCTGGGGAAACTGTCGACGCTCAAGGATGGTGATTTCATCTTGACCGAAAG

>Intron 2

ATGCCCTCCTTCCCTCACCCCTCACCGCATCCGGAGCCCATGTGACCTTGGCTCTCCCCACTGGCCCCGGGCCCCAATGGCCCTCCCATACCCCATGGGGCAGCGAGGGAGGGGAAAGGCGAGGGATCTGGCCGGGCGCGGTGGCTCACGCCTGTCATCCCGGCACTTCGGGAGGCCAAGGCGGGCGGATCACGAGGTCAGGAGACCGAGACCATCCTGGCTAACACCGTGAAACACCGTCTCTACTAAAAATGGAAAAAAAAATTAACCGGGCGTGGTGGCGGGCGTCTGTAGTCCCAGCTACTCTGGAGGCTGAGGCAGGAGAATGGTGTGAACCCAGGAGGCGGAGCTTGCAGTGTGCTGAGATCGCGCCACTGCACTCCAGCCTGGGCGACAGAGCTAGACTCCGTCTCAAAAAGGCCGGGCGCGGTGGTTTACGCCTGTAATCCCAGCACTTTGGGAGGCCGAGGTGGGCGGATCACGAGGTCAGGAGATCGAGACCATCCTGGCTAACACGGTGAAACCCTGTCTCTACTAAAAATACAAAACATTAGCAGGGCGTGGTGGCGGCCGCCTGTAGTCCCAGCTACTGGGGAGGCTGAGGCAGGAGAATGGCGTGAACTCAGGAGGCGGAGCTTGCAATGAGCAGAGGTCGCGCCACTGCACTCCAGCCTGGGTGACAGAGGGAGCCCACTCCAGCCTGGGCGACAGAGGGAGACTCCGTCTCAAAAAAAAAGGAAAGAAAGAAAGGAGAGGTATCTGGGGAGAAGGTACAGCTTGGGGTGTGTCCGGGATGAGCAGGGGCTGACAGAACATGTCCCCCCACCTCTCATCTTCAGCCTTTTCTGAGCCGCAGGGCCTCTCCACTCCCAGACTGAAGGGTATTAGAAGAGAAGACAAGGGAACATTTTTCCACTGTTGCGCATTTGTTCAACAAATGCTAGCTGAAAAGAGCCTCTAGTGACTTGTCGCAGACTACCCAATCTACCCAGGCCGGGCCTAGAGGCCAATGCCATGGCCCAAGGGCACAGCTCATGGTGAGGTCCAGCTGCTGGGCAGGAAAAGGACAAGAGGTCAGGTGGCTGCAGAGGTGATGGCTGGGGGCCTGTCAGACGGGGGCCAAAGACATTCCTCCCCTCGTGATCCCTGACCCAAGCGCGTGGACATGCAAGGGACTCCACGGAGCATCCACTGTGTGCCAGCCCCATGCAGGGTTCCAGGGGTCCAGGGAGCCTATTCTGAGCTGCACCGCCTCGGACAAGTCACTTGACCATTCTGACCTTGAGTTTTCTCTTGTGCTAAAAGGCTAACAGGAGTGTCTGCCTCACAGGGCGGCTGCTGGCATATCACAGAGATGAGGTTCTCAAAATGCAAAGCAGAAGGTCCAGCCAAGAGTCGGTGCCCAAGGCAACAAAGACAGGAGGAGACTCGTAGGAGGAGGGGGTGGTGTTGGGGAGCTGGAGATGGAGGGCGAGGCTGGAGGGCAGTCCTTCAAATGCAGAGAAGCCCCCGGGCCCCACTGGCAGATGGGAGCAGTTAGGGGTAAATGCCTGGTGCCAGTGTCCTTATAGCCACTGCCCATTTGTTCCCAG

>Exon 3

CTCGGCCATCCTGATTTACCTGAGCTGTAAGTACCAGACGCCGGACCACTGGTATCCATCTGACCTGCAGGCTCGTGCCCGTGTTCATGAGTACCTGGGCTGGCATGCCGACTGCATCCGTGGCACCTTTGGTATACCCCTGTGGGTCCAG

>Intron 3

GTGAGGAGAGCCATCTGGAGAGTGATTGGCCATCAGGGAGTAGTTGGCAGTAGGCCGGGGCCATAGACTGACCCACTCTCTGCCCCCATCAG

>Exon 4

ATGTTGGGGCCACTCATTGGGGTCCAGGTGCCCAAGGAGAAGGTGGAACGCAACAGGACTGCCATGGACCAGGCCCTGCAATGGCTGGAGGGCAAGTTCCTGGGGGACAGGCCCTTCCTCGCTGGCCAGCAGGTGACACTGGCTGATCTCATGGCCCTGGAGGAGCTGATGCAG

>Intron 4

GTGTGAGCTCAGCCTGTGGGCAGTGTCCCCCTTCGTGTCACACCCATGAGGCAGACAGAAACACTGAGGACTGGAGAAAGCCAGAACTTTGCCCAGAATCATAGAGCAAGTCTCTGGATGATCTGGGGCCAgAACCCTGAACTTCTGCCTCCTGCCTGGGTGTGGGGTCTCACCCTGGCTGCTCTTGGGCTCTAAGGCTGAACATACTGCCTGGGCCCCTGTGGTCCATTCACTTAGGGGCTGGGGAATGGACCATGTCTCTGATACTTCTGCCCATGGTTCCAGCATTCGGGTCGGCAGTGACAACTGGGAAAGTTGTATGCCCACAACTTTTTCATCCTTGTCCCTACAG

>Exon 5

CCGGTGGCTCTCGGCTATGAACTGTTTGAGGGACGGCCACGACTGGCAGCATGGCGTGGATGAGTGGAGGCTTTCCTGGGTGCTGAGCTACGCCAGGAGGCCCACAGCATCATCTTGAGCATCCTGGAACAGGCGGCCAAGAAAACCCTCCCAACACCCTCACCAGAGGCCTATCAGGCTATGCTGCTTCGAATCGCCAGGATCCCCTGA

>Post

AGGGTCTGGGATGGGGGCCAGGAGATTAGCAACAAGGATTCATTCTGTTACTTACTTGCCCCTTTTTATCTTTCCCTCTTGCCCCAGTCCCTTCTCTCCAGCTTCATGTGAAGCTCTGCACAGACAAGACACTCAGTGTCCTTGGCAGTGCTGCTACTCCTCAGGTGCAGCATACATAACCAGTAAGAGACTAAATCTGCAATATATAAAGAGCTCCTACAAATCAGTAACATGAAGAACACTCAAAAATTGGCAAATGTCATCAGTGTTTTAAACAGAATAAAGATTCCAAACACTTTGAATAGAGAACCAAGAGTTATTGGTTTTACTACATTGTTGTGTTATACATATGGAGTAAAAGTATGTGCTAGTAATCCTCATCATGGTTAATAACAAAGTAACCTCACAATAACGAGTCAACATAATTGTATCACCAGGGCAACAAAATGTTAAGTAAGTAACCAATTCGAATTGCAAACTGTTAAAGGATATAGGCGATGTTTCACAGGGCATAGCAACGGTCTTTGAAGTCTAGGAA

**Accession No. JN819430 - Human Colon-2 (AVA)**

>Promoter

CCGTGAGAGTAACTGGGATTGCAGGTGTGGGCCAGCATGCCCTGTTATCACTCCCTTTCTCTGAGTCAGCTTTTCTCTCTCAGTCTCGTCTACTCCTCGAACTCAGGAAATGTCACTACAGGTGGCCCCAGCTCCCGTTTACCTCCCCATAGGGAGCTCCTCTCTCCAGTTCCAGTTTCAAAACTCCCAAGGAAGCATTCTGGTTCACTCGCTTGGGCCACTGGCCAGAGGGATGGGATACTCTGAAAGATTCAGCTAGAGTCCCGGGCCCAGCCCTGGACCATCACTGTGCCCCCTGGTGAGATGCCAGGGCTGGGATTCAGGGAGAAGAAAGGAGGTTCCCGGACAGTCATTCCTGCCTCCCGCGGCTGCGGGCTCCCTGCCCCCATCCTGTGCACGAAGTGGGAGCTCCCGCTGTCTGGCAGCTCCCGCTGTCTGGCAGCAGCTGCTCTGCAGGGGACAGTCTGGACGGCAGAAAGTTCATCCTTAACCCCAGCCTTCCAGTCAAGGTTCCCACCAGTTTGGGACACCTGCAAGTGTCACATCCCACTGGGTGAAACTCTAAGATCCCTTTTAGGGGATCCCATTCGCTCCCTCCCTTCCGCCACCATGCAGCGCCGAGAAACAGAGCTCTGAACGAACCCTCAGATGTCCGTGCGCTGGGGCCTTTCCAGGACGGCGGCGCCCAGTCGTTTCTGGGTCAGGGCGACGCCTGGAACTGGGCAGGGTCCCTGGCACCGGGATCCCGAAAAGCAGACCTGCTTCTCCCTGTCCAGCCGGTTCCCCTTCCCCTTGCAGTCGGCCCCCTGCATCCGCGTCCTCCCTGCCAGTCGAGGGTCCCCAGCTCCAACTCCACCCTCCCAGCTGTGCGTTCATAGCGACCGCCCTCCCTGTAGGGACGCACGGATCTGGTGGTGGAGTCTTGGCCGGCAGGACTGGACAGGAACCGAAGGGGCGAGGCGGGTCCGGGGGTGGTGCGCTCCAATTGGGTGCTGTCCCCAGGGGGTGGGGCCTGATCCCCTATTTCCCGGCGCGCCGG

>Exon 1

GATCCTGCCACAGCTGCTGCCCACACCGCGCTCAGCGCCTTCACTGCCATCCCCGCTGTCCTTGCCGCCCCCGCCATGGGCCTAGAGCTGTTTCTTGACCTGGTGTCCCAGCCCAGCCGCGCCGTCTACATCTTCGCCAAGAAGAATGGCATCCCCTTAGAGCTGCGCACCGTGGATTTGGTCAAAG

>Intron 1

GTGGGCCCAGCCCGTTTCCCCGCGTGTCCACAAACCCAGTGCACCCCCAGGCCCCCGCCCTGCTCTGCCCTGAGCGTCTCGCCGCCCGCACAGCCCCCTCACCTCCTCCTGCAGCGTCTGCCACCAGAGAATGCTGTGGACTGAGTGGCCTTGGAGGGATCACAGCCTCTCTGAACCTTAGCTTGCCTTCTGAAAAGGAGGATAATGTTACCTTCTGCTCTGTAGGGATGGAAAGAAAATACTGAATGGAGTTGACAGAGTTCTTGCGTGGAATGCACGCATATAAATTCACAAAGCCCAGAAGACCTCGGGAAGAAGGACATACTGTTGTGAGGATTAAGAGATGGGAAGAGATGAGCCACCCCAGTTTGCCTCCCCTCCCCTGGCCCACCAGAGTCCGGCTAGAAAACTTCTCTTTATCCACCTGCTGCACCTGGCCCCACCCACCAAAACCCCCCAGCTGCCCCGGAATGTGGCAGGGCAGGGAGGCCCAGCCAGGGAGTGAGGCTGATCCAGGCCTCTAGTCCCAGACCTTGCTGTTTCTCAGGGCTGTGGGGCTCCGCTTGGGGAGGAGGAGGGAGGGTGTAGAGGTGCAGCGTTTTTACTCTGAAGATCTTTTCTGACTTCTTCTTCTTCAG

>Exon 2

GGCAGCACAAGAGCAAGGAGTTCTTGCAGATCAACAGCCTGGGGAAACTGCCGACGCTCAAGGATGGTGATTTCATCTTGACCGAAAG

>Intron 2

ATGCCCTCCTTCCCTCACCCCTCACCGCATCCGGAGCCCATGTGACCTTGGCTCTCCCCACTGGCCCCGGGCCCCAATGGCCCTCCCATACCCCATGGGGCAGCGAGGGAGGGGAAAGGCGAGGGATCTGGCCGGGCGCGGTGGCTCACGCCTGTCATCCCGGCACTTCGGGAGGCCAAGGCGGGCGGATCACGAGGTCAGGAGACCGAGACCATCCTGGCTAACACCGTGAAACACCGTCTCTACTAAAAATGGAAAAAAAAATTAACCGGGCGTGGTGGCGGGCGTCTGTAGTCCCAGCTACTCTGGAGGCTGAGGCAGGAGAATGGTGTGAACCCAGGAGGCGGAGCTTGCAGTGTGCTGAGATCGCGCCACTGCACTCCAGCCTGGGCGACAGAGCTAGACTCCGTCTCAAAAAGGCCGGGCGCGGTGGTTTACGCCTGTAATCCCAGCACTTTGGGAGGCCGAGGTGGGCGGATCACGAGGTCAGGAGATCGAGACCATCCTGGCTAACACGGTGAAACCCTGTCTCTACTAAAAATACAAAACATTAGCAGGGCGTGGTGGCGGCCGCCTGTAGTCCCAGCTACTGGGGAGGCTGAGGCAGGAGAATGGCGTGAACTCAGGAGGCGGAGCTTGCAATGAGCAGAGGTCGCGCCACTGCACTCCAGCCTGGGTGACAGAGGGAGCCCACTCCAGCCTGGGCGACAGAGGGAGACTCCGTCTCAAAAAAAAAGGAAAGAAAGAAAGGAGAGGTATCTGGGGAGAAGGTACAGCTTGGGGTGTGTCCGGGATGAGCAGGGGCTGACAGAACATGTCCCCCCACCTCTCATCTTCAGCCTTTTCTGAGCCGCAGGGCCTCTCCACTCCCAGACTGAAGGGTATTAGAAGAGAAGACAAGGGAACATTTTTCCACTGTTGCGCATTTGTTCAACAAATGCTAGCTGAAAAGAGCCTCTAGTGACTTGTCGCAGACTACCCAATCTACCCAGGCCGGGCCTAGAGGCCAATGCCATGGCCCAAGGGCACAGCTCATGGTGAGGTCCAGCTGCTGGGCAGGAAAAGGACAAGAGGTCAGGTGGCTGCAGAGGTGATGGCTGGGGGCCTGTCAGACGGGGGCCAAAGACATTCCTCCCCTCGTGATCCCTGACCCAAGCGCGTGGACATGCAAGGGACTCCACGGAGCATCCACTGTGTGCCAGCCCCATGCAGGGTTCCAGGGGTCCAGGGAGCCTATTCTGAGCTGCACCGCCTCGGACAAGTCACTTGACCATTCTGACCTTGAGTTTTCTCTTGTGCTAAAAGGCTAACAGGAGTGTCTACCTCACAGGGCGGCTGCTGGCATATCACAGAGATGAGGTTCTCAAAATGCAAAGCAGAAGGTCCAGCCAAGAGTCGGTGCCCAAGGCAACAAAGACAGGAGGAGACTCGTAGGAGGAGGGGGTGGTGTTGGGGAGCTGGAGATGGAGGGCGAGGCTGGAGGGCAGTCCTTCAAATGCAGAGAAGCCCCCGGGCCCCACTGGCAGATGGGAGCAGTTAGGGGTAAATGCCTGGTGCCAGTGTCCTTATAGCCACTGCCCATTTGTTCCCAG

>Exon 3

CTCGGCCATCCTGATTTACCTGAGCTGTAAGTACCAGACGCCGGACCACTGGTATCCATCTGACCTGCAGGCTCGTGCCCGTGTTCATGAGTACCTGGGCTGGCATGCCGACTGCATCCGTGGCACCTTTGGTATACCCCTGTGGGTCCAG

>Intron 3

GTGAGGAGAGCCATCTGGAGAGTGATTGGCCATCAGGGAGTAGTTGGCAGTAGGCCGGGGCCATAGACTGACCCACTCTCTGCCCCCATCAG

>Exon 4

ATGTTGGGGCCACTCATTGGGGTCCAGGTGCCCAAGGAGAAGGTGGAACGCAACAGGACTGCCATGGACCAGGCCCTGCAATGGCTGGAGGACAAGTTCCTGGGGGACAGGCCCTTCCTCGCTGGCCAGCAGGTGACACTGGCTGATCTCATGGCCCTGGAGGAGCTGATGCAG

>Intron 4

GTGTGAGCTCAGCCTGTGGGCAGTGTCCCTCTTCGTGTCACACCCATGAGGCAGACAGAAACACTGAGGACTGGAGAAAGCCAGAACTTTGCCCAGAATCATAGAGCAAGTCTCTGGATGATCTGGGGCCAGAACCCTGAACTTCTGCCTCCTGCCTGGGTGTGGGGTCTCACCCTGGCTGCTCTTGGGCTCTAAGGCTGAACATACTGCCTGGGCCCCTGTGGTCCATTCACTTAGGGGCTGGGGAATGGACCATGTCTCTGATACTTCTGCCCATGGTTCCAGCATTCGGGTCGGCAGTGACAACTGGGAAAGTTGTATGCCCACAACTTTTTCATCCTTGTCCCTACAG

>Exon 5

CCGGTGGCTCTCGGCTATGAACTGTTTGAGGGACGGCCACGACTGGCAGCATGGCGTGGATGAGTGGAGGCTTTCCTGGGTGCTGAGCTATGCCAGGAGGCCCACAGCATCATCTTGAGCATCCTGGAACAGGCGGCCAAGAAAACCCTCCCAACACCCTCACCAGAGGCCTATCAGGCTATGCTGCTTCGAATCGCCAGGATCCCCTGA

>Post

AGGGTCTGGGATGGGGGCCAGGAGATTAGCAACAAGGATTCATTCTGTTACTTACTTGCCCCTTTTTATCTTTCCCTCTTGCCCCAGTCCCTTCTCTCCAGCTTCATGTGAAGCTCTGCACAGACAAGACACTCAGTGTCCTTGGCAGTGCTGCTACTCCTCAGGTGCAGCATACATAACCAGTAAGAGACTAAATCTGCAATATATAAAGAGCTCCTACAAATCAGTAACATGAAGAACACTCAAAAATTGGCAAATGTCATCAGTGTTTTAAACAGAATAAAGATTCCAAACACTTTGAATAGAGAACCAAGAGTTATTGGTTTTACTACATTGTTGTGTTATACATATGGAGTAAAAGTATGTGCTAGTAATCCTCATCATGGTTAATAACAAAGTAACCTCACAATAACGAGTCAACATAATTGTATCACCAGGGCAACAAAATGTTAAGTAAGTAACCAATTCGAATTGCAAACTGTTAAAGGATATAGGCGATGTTTCACAGGGCATAGCAACGGTCTTTGAAGTCTAGGAA

**Accession No. JN819431 - Human Colon-2 (NO AVA)**

>Promoter

CCGTGAGAGTAACTGGGATTGCAGGTGTGGGCCAGCATGCCCTGTTATCACTCCCTTTCTCTGAGTCAGCTTTTCTCTCTCAGTCTCGTCTACTCCTCGAACTCAGGAAATGTCACTACAGGTGGCCCCAGCTCCCGTTTACCTCCCCATAGGGAGCTCCTCTCTCCAGTTCCAGTTTCAAAACTCCCAAGGAAGCATTCTGGTTCACTCGCTTGGGCCACTGGCCAGAGGGATGGGATACTCTGAAAGATTCAGCTAGAGTCCCGGGCCCAGCCCTGGACCATCACTGTGCCCCCTGGTGAGATGCCAGGGCTGGGATTCAGGGAGAAGAAAGGAGGTTCCCGGACAGTCATTCCTGCCTCCCGCGGCTGCGGGCTCCCTGCCCCCATCCTGTGCACGAAGTGGGAGCTCCCGCTGTCTGGCAGCTCCCGCTGTCTGGCAGCAGCTGCTCTGCAGGGGACAGTCTGGACGGCAGAAAGTTCATCCTTAACCCCAGCCTTCCAGTCAAGGTTCCCACCAGTTTGGGACACCTGCAAGTGTCACATCCCACTGGGTGAAACTCTAAGATCCCTTTTAGGGGATCCCATTCGCTCCCTCCCTTCCGCCACCATGCAGCGCCGAGAAACAGAGCTCTGAACGAACCCTCAGATGTCCGTGCGCTGGGGCCTTTCCAGGACGGCGGCGCCCAGTCGTTTCTGGGTCAGGGCGACGCCTGGAACTGGGCAGGGTCCCTGGCACCGGGATCCCGAAAAGCAGACCTGCTTCTCCCTGTCCAGCCGGTTCCCCTTCCCCTTGCAGTCGGCCCCCTGCATCCGCGTCCTCCCTGCCAGTCGAGGGTCCCCAGCTCCAACTCCACCCTCCCAGCTGTGCGTTCATAGCGACCGCCCTCCCTGTAGGGACGCACGGATCTGGTGGTGGAGTCTTGGCCGGCAGGACTGGACAGGAACCGAAGGGGCGAGGCGGGTCCGGGGGTGGTGCGCTCCAATTGGGTGCTGTCCCCAGGGGGTGGGGCCTGATCCCCTATTTCCCGGCGCGCCGG

>Exon 1

GATCCTGCCACAGCTGCTGCCCACACCGCGCTCAGCGCCTTCACTGCCATCCCCGCTGTCCTTGCCGCCCCCGCCATGGGCCTAGAGCTGTTTCTTGACCTGGTGTCCCAGCCCAGCCGCGCCGTCTACATCTTCGCCAAGAAGAATGGCATCCCCTTAGAGCTGCGCACCGTGGATTTGGTCAAAG

>Intron 1

GTGGGCCCAGCCCGTTTCCCCGCGTGTCCACAAACCCAGTGCACCCCCAGGCCCCCGCCCTGCTCTGCCCTGAGCGTCTCGCCGCCCGCACAGCCCCCTCACCTCCTCCTGCAGCGTCTGCCACCAGAGAATGCTGTGGACTGAGTGGCCTTGGAGGGATCACAGCCTCTCTGAACCTTAGCTTGCCTTCTGAAAAGGAGGATAATGTTACCTTCTGCTCTGTAGGGATGGAAAGAAAATACTGAATGGAGTTGACAGAGTTCTTGCGTGGAATGCACGCATATAAATTCACAAAGCCCAGAAGACCTCGGGAAGAAGGACATACTGTTGTGAGGATTAAGAGATGGGAAGAGATGAGCCACCCCAGTTTGCCTCCCCTCCCCTGGCCCACCAGAGTCCGGCTAGAAAACTTCTCTTTATCCACCTGCTGCACCTGGCCCCACCCACCAAAACCCCCCAGCTGCCCCGGAATGTGGCAGGGCAGGGAGGCCCAGCCAGGGAGTGAGGCTGATCCAGGCCTCTAGTCCCAGACCTTGCTGTTTCTCAGGGCTGTGGGGCTCCGCTTGGGGAGGAGGAGGGAGGGTGTAGAGGTGCAGCGTTTTTACTCTGAAGATCTTTTCTGACTTCTTCTTCTTCAG

>Exon 2

GGCAGCACAAGAGCAAGGAGTTCTTGCAGATCAACAGCCTGGGGAAACTGCCGACGCTCAAGGATGGTGATTTCATCTTGACCGAAAG

>Intron 2

ATGCCCTCCTTCCCTCACCCCTCACCGCATCCGGAGCCCATGTGACCTTGGCTCTCCCCACTGGCCCCGGGCCCCAATGGCCCTCCCATACCCCATGGGGCAGCGAGGGAGGGGAAAGGCGAGGGATCTGGCCGGGCGCGGTGGCTCACGCCTGTCATCCCGGCACTTCGGGAGGCCAAGGCGGGCGGATCACGAGGTCAGGAGACCGAGACCATCCTGGCTAACACCGTGAAACACCGTCTCTACTAAAAATGGAAAAAAAAATTAACCGGGCGTGGTGGCGGGCGTCTGTAGTCCCAGCTACTCTGGAGGCTGAGGCAGGAGAATGGTGTGAACCCAGGAGGCGGAGCTTGCAGTGTGCTGAGATCGCGCCACTGCACTCCAGCCTGGGCGACAGAGCTAGACTCCGTCTCAAAAAGGCCGGGCGCGGTGGTTTACGCCTGTAATCCCAGCACTTTGGGAGGCCGAGGTGGGCGGATCACGAGGTCAGGAGATCGAGACCATCCTGGCTAACACGGTGAAACCCTGTCTCTACTAAAAATACAAAACATTAGCAGGGCGTGGTGGCGGCCGCCTGTAGTCCCAGCTACTGGGGAGGCTGAGGCAGGAGAATGGCGTGAACTCAGGAGGCGGAGCTTGCAATGAGCAGAGGTCGCGCCACTGCACTCCAGCCTGGGTGACAGAGGGAGCCCACTCCAGCCTGGGCGACAGAGGGAGACTCCGTCTCAAAAAAAAAGGAAAGAAAGAAAGGAGAGGTATCTGGGGAGAAGGTACAGCTTGGGGTGTGTCCGGGATGAGCAGGGGCTGACAGAACATGTCCCCCCACCTCTCATCTTCAGCCTTTTCTGAGCCGCAGGGCCTCTCCACTCCCAGACTGAAGGGTATTAGAAGAGAAGACAAGGGAACATTTTTCCACTGTTGCGCATTTGTTCAACAAATGCTAGCTGAAAAGAGCCTCTAGTGACTTGTCGCAGACTACCCAATCTACCCAGGCCGGGCCTAGAGGCCAATGCCATGGCCCAAGGGCACAGCTCATGGTGAGGTCCAGCTGCTGGGCAGGAAAAGGACAAGAGGTCAGGTGGCTGCAGAGGTGATGGCTGGGGGCCTGTCAGACGGGGGCCAAAGACATTCCTCCCCTCGTGATCCCTGACCCAAGCGCGTGGACATGCAAGGGACTCCACGGAGCATCCACTGTGTGCCAGCCCCATGCAGGGTTCCAGGGGTCCAGGGAGCCTATTCTGAGCTGCACCGCCTCGGACAAGTCACTTGACCATTCTGACCTTGAGTTTTCTCTTGTGCTAAAAGGCTAACAGGAGTGTCTACCTCACAGGGCGGCTGCTGGCATATCACAGAGATGAGGTTCTCAAAATGCAAAGCAGAAGGTCCAGCCAAGAGTCGGTGCCCAAGGCAACAAAGACAGGAGGAGACTCGTAGGAGGAGGGGGTGGTGTTGGGGAGCTGGAGATGGAGGGCGAGGCTGGAGGGCAGTCCTTCAAATGCAGAGAAGCCCCCGGGCCCCACTGGCAGATGGGAGCAGTTAGGGGTAAATGCCTGGTGCCAGTGTCCTTATAGCCACTGCCCATTTGTTCCCAG

>Exon 3

CTCGGCCATCCTGATTTACCTGAGCTGTAAGTACCAGACGCCGGACCACTGGTATCCATCTGACCTGCAGGCTCGTGCCCGTGTTCATGAGTACCTGGGCTGGCATGCCGACTGCATCCGTGGCACCTTTGGTATACCCCTGTGGGTCCAG

>Intron 3

GTGAGGAGAGCCATCTGGAGAGTGATTGGCCATCAGGGAGTAGTTGGCAGTAGGCCGGGGCCATAGACTGACCCACTCTCTGCCCCCATCAG

>Exon 4

ATGTTGGGGCCACTCATTGGGGTCCAGGTGCCCAAGGAGAAGGTGGAACGCAACAGGACTGCCATGGACCAGGCCCTGCAATGGCTGGAGGACAAGTTCCTGGGGGACAGGCCCTTCCTCGCTGGCCAGCAGGTGACACTGGCTGATCTCATGGCCCTGGAGGAGCTGATGCAG

>Intron 4

GTGTGAGCTCAGCCTGTGGGCAGTGTCCCTCTTCGTGTCACACCCATGAGGCAGACAGAAACACTGAGGACTGGAGAAAGCCAGAACTTTGCCCAGAATCATAGAGCAAGTCTCTGGATGATCTGGGGCCAGAACCCTGAACTTCTGCCTCCTGCCTGGGTGTGGGGTCTCACCCTGGCTGCTCTTGGGCTCTAAGGCTGAACATACTGCCTGGGCCCCTGTGGTCCATTCACTTAGGGGCTGGGGAATGGACCATGTCTCTGATACTTCTGCCCATGGTTCCAGCATTCGGGTCGGCAGTGACAACTGGGAAAGTTGTATGCCCACAACTTTTTCATCCTTGTCCCTACAG

>Exon 5

CCGGTGGCTCTCGGCTATGAACTGTTTGAGGGACGGCCACGACTGGCAGCATGGCGTGGATGAGTGGAGGCTTTCCTGGGTGCTGAGCTATGCCAGGAGGCCCACAGCATCATCTTGAGCATCCTGGAACAGGCGGCCAAGAAAACCCTCCCAACACCCTCACCAGAGGCCTATCAGGCTATGCTGCTTCGAATCGCCAGGATCCCCTGA

>Post

AGGGTCTGGGATGGGGGCCAGGAGATTAGCAACAAGGATTCATTCTGTTACTTACTTGCCCCTTTTTATCTTTCCCTCTTGCCCCAGTCCCTTCTCTCCAGCTTCATGTGAAGCTCTGCACAGACAAGACACTCAGTGTCCTTGGCAGTGCTGCTACTCCTCAGGTGCAGCATACATAACCAGTAAGAGACTAAATCTGCAATATATAAAGAGCTCCTACAAATCAGTAACATGAAGAACACTCAAAAATTGGCAAATGTCATCAGTGTTTTAAACAGAATAAAGATTCCAAACACTTTGAATAGAGAACCAAGAGTTATTGGTTTTACTACATTGTTGTGTTATACATATGGAGTAAAAGTATGTGCTAGTAATCCTCATCATGGTTAATAACAAAGTAACCTCACAATAACGAGTCAACATAATTGTATCACCAGGGCAACAAAATGTTAAGTAAGTAACCAATTCGAATTGCAAACTGTTAAAGGATATAGGCGATGTTTCACAGGGCATAGCAACGGTCTTTGAAGTCTAGGAA

**Accession No. JN819428 - Human Colon-3 (AVA)**

>Promoter

CCGTGAGAGTAACTGGGATTGCAGGTGTGGGCCAGCATGCCCTGTTATCACTCCCTTTCTCTGAGTCAGCTTTTCTCTCTCAGTCTCGTCTACTCCTCGAACTCAGGAAATGTCACTACAGGTGGCCCCAGCTCCCGTTTACCTCCCCATAGGGAGCTCCTCTCTCCAGTTCCAGTTTCAAAACTCCCAAGGAAGCATTCTGGTTCACTCGCTTGGGCCACTGGCCAGAGGGATGGGATACTCTGAAAGATTCAGCTAGAGTCCCGGGCCCAGCCCTGGACCATCACTGTGCCCCCTGGTGAGATGCCAGGGCTGGGATTCAGGGAGAAGAAAGGAGGTTCCCGGACAGTCATTCCTGCCTCCCGCGGCTGCGGGCTCCCTGCCCCCATCCTGTGCACGAAGTGGGAGCTCCCGCTGTCTGGCAGCTCCCGCTGTCTGGCAGCAGCTGCTCTGCAGGGGACAGTCTGGACGGCAGAAAGTTCATCCTTAACCCCAGCCTTCCAGTCAAGGTTCCCACCAGTTTGGGACACCTGCAAGTGTCACATCCCACTGGGTGAAACTCTAAGATCCCTTTTAGGGGATCCCATTCGCTCCCTCCCTTCCGCCACCATGCAGCGCCGAGAAACAGAGCTCTGAACGAACCCTCAGATGTCCGTGCGCTGGGGCCTTTCCAGGACGGCGGCGCCCAGTCGTTTCTGGGTCAGGGCGACGCCTGGAACTGGGCAGGGTCCCTGGCACCGGGATCCCGAAAAGCAGACCTGCTTCTCCCTGTCCAGCCGGTTCCCCTTCCCCTTGCAGTCGGCCCCCTGCATCCGCGTCCTCCCTGCCAGTCGAGGGTCCCCAGCTCCAACTCCACCCTCCCAGCTGTGCGTTCATAGCGACCGCCCTCCCTGTAGGGACGCACGGATCTGGTGGTGGAGTCTTGGCCGGCAGGACTGGACAGGAACCGAAgGGGCGAGGCGGGTCCGGGGGTGGTGCGCTCCAATTGGGTGCTGTCCCCAGGGGGTGGGGCCTGATCCCCTATTTCCCGGCGCGCCGG

>Exon 1

GATCCTGCCACAGCTGCTGCCCACACCGCGCTCAGCGCCTTCACTGCCATCCCCGCTGTCCTTGCCGCCCCCGCCATGGGCCTAGAGCTGTTTCTTGACCTGGTGTCCCAGCCCAGCCGCGCCGTCTACATCTTCGCCAAGAAGAATGGCATCCCCTTAGAGCTGCGCACCGTGGATTTGGTCAAAG

>Intron 1

GTGGGCCCAGCCCGTTTCCCCGCGTGTCCACAAACCCAGTGCACCCCCAGGCCCCCGCCCTGCTCTGCCCTGAGCGTCTCGCCGCCCGCACAGCCCCCTCACCTCCTCCTGCAGCGTCTGCCACCAGAGAATGCTGTGGACTGAGTGGCCTTGGAGGGATCACAGCCTCTCTGAACCTTAGCTTGCCTTCTGAAAAGGAGGATAACGTTACCTTCTGCTCTGTAGGGATGGAAAGAAAATACTGAATGGAGTTGACAGAGTTCTTGCGTGGAATGCACGCATATAAATTCACAAAGCCCAGAAGACCTCGGGAAGAAGGACATGCTGTTGTGAGAATTAAGAGATGGGAAGAGATGAGCCACCCCAGTTTGCCTCCCCTCCCCTGGCCCACCAGAGTCCGGCTAGAAAACTTCTCTTTATCCACCTGCTGCACCTGGCCCCACCCACCAAAGCCCCCCAGCTGCCCCGGAATGTGGCAGGGCAGGGAGGCCCAGCCAGGGAGTGAGGCTGATCCAGGCCTCTAGTCCCAGACCTTGCTGTTTCTCAGGGCTGTGGGGCTCCGCTTGGGGAGGAGGAGGGAGGGTGTAGAGGTGCAGCGTTTTTACTCTGAAGACCTTTTCTGACTTCTTCCTCTTCAG

>Exon 2

GGCAGCACAAGAGCAAGGAGTTCTTGCAGATCAACAGCCTGGGGAAACTGCCGACGCTCAAGGATGGTGATTTCATCTTGACCGAAAG

>Intron 2

GTGCCCTCCTTCCCTCACCCCTCACCGCATCCGGAGCCCATGTGACCTTGGCTCTCCCCACTGGCCCCGGGCCCCAGTGGCCCTCCCATACCCCATGGGGCAGCGAGGGAGGGGAAAGGCGAGGGATCTGGCCGGGCGCGGTGGCTCACGCCTGTCATCCCGGCACTTCGGGAGGCCAAGGCGGGCGGATCACGAGGTCAGGAGACCGAGACCATCCTGGCTAACACCGTGAAACACCGTCTCTACTAAAAATGGAAAAAAAGTTAACCGGGCGTGGTGGCGGGCGTCTGTAGTCCCAGCTACTCTGGAGGCTGAGGCAGGAGAATGGTGTGAACCCAGGAAGCGGAGCTTGCAGTGTGCTGAGATCGCGCCACTGCACTCCAGCCTGGGCGACAGAGCTAGACTCCGTCTCAAAAAGGCCGGGCGCGGTGGTTTACGCCTGTAATCCCAGCACTTTGGGAGGCCGAGGTGGGCGGATCACGAGGTCAGGAGATCGAGACCATCCTGGCTAACACGGTGAAACCCTGTCTCTACTAAAAATACAAAACATTAGCAGGGCGTGGTGGCGGCCGCCTGTAGTCCCAGCTACTGGGGAGGCTGAGGCAGGAGAATGGCGTGAACCCAGGAGGCGGAGCTTGCAATGAGCAGAGATAGCGCCACTGCACTCCAGCCTGGGTGACAGAGGGAGCCCACTCCAGCCTGGGCGACAGAGGGAGACTCCGTCTCAAAAAAAAAGGAAAGAAAGAAAGGAGAGGTATCTGGGGAGAAGGTACAGCTTGGGGTGTGACCGGGATGAGCAGGGGCTGACAGAACATGTCCCCCCACCTCTCATCTTCCAGCCTTTTCTGAGCCGCAGGGCCTCTCCACTCCCAGACTGAAGGGTATTAGAAGAGAAGACAAGGGAACATTTTTCCACTGTTGCGCATTTGTTCAACAAATGCTAGCTGAAAAGAGCCTCTAGTGACTTGTCGCAGACTACCCAATCTACCCAGGCCGGGCCTAGAGGCCAATGCCATGGCCCAAAGGCACAGCTCATGGTGAGGTCCAGCTGCTGGGCAGGAAAAGGACAAGAGGTCAGGTGGCTGCAGAGGTGATGGCTGGGGGCCTGTCAGACGGGGGCCAAAGACATTCCTCCCCTCGTGATCCCTGACCCAAGCGCGTGGACATGCAAGGGACTCCACGGAGCATCCACTGTGTGCCAGCCCCATGCAGGGTTCCAGGGGTCCAGGGAGCCTATTCTGAGCTGCACCGCCTCGGACAAGTCACTTGACCATTCTGACCTTGAGTTTTCTCTTGTGCTAAAAGGCTAACAGGAGTGTCTACCTCACAGGGCGGCTGCTGGCATATCACAGAGATGAGGTTCTCAAAATGCAAAGCAGAAGGTCCAGCCAAGAGTCGGTGCCCAAGGCAACAAAGACAGGAGGAGACTCGTAGGAGGAGGGGGTGGTGTTGGGGAGCTGGAGATGGAGGGCGAGGCTGGAGGGCAGTCCTTCAAATGCAGAGAAGCCCCCGGGCCCCACTGGCAGATGGGAGCAGTTAGGGGTAAATGCCTGGTGCCAGTGTCCTTATAGCCACTGCCCATTTGTTCCCAG

>Exon 3

CTCGGCCATCCTGATTTACCTGAGCTGTAAGTACCAGACGCCGGACCACTGGTATCCATCTGACCTGCAGGCTCGTGCCCGTGTTCATGAGTACCTGGGCTGGCATGCCGACTGCATCCGTGGCACCTTTGGTATACCCCTGTGGGTCCAG

>Intron 3

GTGAGGAGAGCCATCTGGAGAGTGATTGGCCATCAGAGAGTAGTTGGCAGTAGGTCGGGGCCATAGACTGACCCACTGTCTGCCCCCATCAG

>Exon 4

GTGTTGGGGCCACTCATTGGGGTCCAGGTGCCCGAGGAGAAGGTGGAACGCAACAGGACTGCCATGGACCAGGCCCTGCAATGGCTGGAGGACAAGTTCCTGGGGGACAGGCCCTTCCTCGCTGGCCAGCAGGTGACACTGGCTGATCTCATGGCCCTGGAGGAGCTGATGCAG

>Intron 4

GTGTGAGCTCAGCCTGTGGGCAGTGTCCCTCTTCGTGTCACACCCATGAGGCAGACAGAAACACTGAGGCCTGGAGAAAGCCAGAACTTTGCCCAGAATCATAGAGCAAGTCTCTGGATGATCTGGGGCCAGAACCCTGAACTTCTGCCTCCTGCCTGGGTGTGGGGTCTCACCCTGGCTGCTCTTGGGCTCTAAGGCTGAACATACTGCCTGGGCCCCTGTGGTCCATTCACTTAGGGGCTGGGGAATGGACCATGTCTCTGATACTTCTGCCCATGGTTCCAGCATTCGGGTCGGCAGTGACAACTGGGAAAGTTGTATGCCCACAACTTTTTCATCCTTGTCCCTACAG

>Exon 5

CCGGTGGCTCTCGGCTACGAACTGTTTGAGGGACGGCCACGACTGGCAGCATGGCGTGGACGAGTGGAGGCTTTCCTGGGTGCTGAGCTATGCCAGGAGGCCCACAGCATCATCTTGAGCATCCTGGAACAGGCGGCCAAGAAAACCCTCCCAACACCCTCACCAGAGGCCTATCAGGCTATGCTGCTTCGAATCGCCAGGATCCCCTGA

>Post

AGGGTCTGGGATGGGGGCCAGGAGATTAGCAACAAGGATTCATTCTGTTACTTACTTGCCCCTTTTTATCTTTCCCTCTTGCCCCAGTCCCTTCTCTCCAGCTTCATGTGAAGCTCTGCACAGACAAGACACTCAGTGTCCTTGGCAGTGCTGCTACTCCTCAGGTGCAGCATACATAACCAGTAAGAGACTAAATCTGCAATATATAAAGAGCTCCTACAAATCAGTAACATGAAGAACACTCAAAAATTGGCAAATGTCATCAGTGTTTTAAACAGAATAAAGATTCCAGACACTTTGAATAGAGAACCAAGAGTTATTGGTTTTACTACATTGTTGTGTTATACATATGGAGTAAAAGTATGTGCTAGTAATCCTCATCATGGTTAATAACAAAGTAACCTCACAATAACGAGTCAACATAATTGTATCACCAGGGCAACAAAATGTTAAGTAAGTAACCAATTCGAATTGCAAACTGTTAAAGGATATAGGCGATGTTTCACAGGGCATAGCAACGGTCTTTGAAGTCTAGGAA

**Accession No. JN819429 - Human Colon-3 (NO AVA)**

>Promoter

CCGTGAGAGTAACTGGGATTGCAGGTGTGGGCCAGCATGCCCTGTTATCACTCCCTTTCTCTGAGTCAGCTTTTCTCTCTCAGTCTCGTCTACTCCTCGAACTCAGGAAATGTCACTACAGGTGGCCCCAGCTCCCGTTTACCTCCCCATAGGGAGCTCCTCTCTCCAGTTCCAGTTTCAAAACTCCCAAGGAAGCATTCTGGTTCACTCGCTTGGGCCACTGGCCAGAGGGATGGGATACTCTGAAAGATTCAGCTAGAGTCCCGGGCCCAGCCCTGGACCATCACTGTGCCCCCTGGTGAGATGCCAGGGCTGGGATTCAGGGAGAAGAAAGGAGGTTCCCGGACAGTCATTCCTGCCTCCCGCGGCTGCGGGCTCCCTGCCCCCATCCTGTGCACGAAGTGGGAGCTCCCGCTGTCTGGCAGCTCCCGCTGTCTGGCAGCAGCTGCTCTGCAGGGGACAGTCTGGACGGCAGAAAGTTCATCCTTAACCCCAGCCTTCCAGTCAAGGTTCCCACCAGTTTGGGACACCTGCAAGTGTCACATCCCACTGGGTGAAACTCTAAGATCCCTTTTAGGGGATCCCATTCGCTCCCTCCCTTCCGCCACCATGCAGCGCCGAGAAACAGAGCTCTGAACGAACCCTCAGATGTCCGTGCGCTGGGGCCTTTCCAGGACGGCGGCGCCCAGTCGTTTCTGGGTCAGGGCGACGCCTGGAACTGGGCAGGGTCCCTGGCACCGGGATCCCGAAAAGCAGACCTGCTTCTCCCTGTCCAGCCGGTTCCCCTTCCCCTTGCAGTCGGCCCCCTGCATCCGCGTCCTCCCTGCCAGTCGAGGGTCCCCAGCTCCAACTCCACCCTCCCAGCTGTGCGTTCATAGCGACCGCCCTCCCTGTAGGGACGCACGGATCTGGTGGTGGAGTCTTGGCCGGCAGGACTGGACAGGAACCGAAGGGGCGAGGCGGGTCCGGGGGTGGTGCGCTCCAATTGGGTGCTGTCCCCAGGGGGTGGGGCCTGATCCCCTATTTCCCGGCGCGCCGG

>Exon 1

GATCCTGCCACAGCTGCTGCCCACACCGCGCTCAGCGCCTTCACTGCCATCCCCGCTGTCCTTGCCGCCCCCGCCATGGGCCTAGAGCTGTTTCTTGACCTGGTGTCCCAGCCCAGCCGCGCCGTCTACATCTTCGCCAAGAAGAATGGCATCCCCTTAGAGCTGCGCACCGTGGATTTGGTCAAAG

>Intron 1

GTGGGCCCAGCCCGTTTCCCCGCGTGTCCACAAACCCAGTGCACCCCCAGGCCCCCGCCCTGCTCTGCCCTGAGCGTCTCGCCGCCCGCACAGCCCCCTCACCTCCTCCTGCAGCGTCTGCCACCAGAGAATGCTGTGGACTGAGTGGCCTTGAAGGGATCACAGCCTCTCTGAACCTTAGCTTGCCTTCTGAAAAGGAGGATAATGTTACCTTCTGCTCTGTAGGGATGGAAAGAAAATACTGAATGGAGTTGACAGAGTTCTTGCGTGGAATGCACGCATATAAATTCACAAAGCCCAGAAGACCTCGGGAAGAAGGACATACTGTTGTGAGAATTAAGAGATGGGAAGAGATGAGCCACCCCAGTTTGCCTCCCCTCCCCTGGCCCACCAGAGTCCGGCTAGAAAACTTCTCTTTATCCACCTGCTGCACCTGGCCCCACCCACCAAAACCCCCCAGCTGCCCCGGAATGTGGCAGGGCAGGGAGGCCCAGCCAGGGAGTGAGGCTGATCCAGGCCTCTAGTCCCAGACCTTGCTGTTTCTCAGGGCTGTGGGGCTCCGCTTGGGGAGGAGGAGGGAGGGTGTAGAGGTGCAGCGTTTTTACTCTGAAGACCTTTTCTGACTTCTTCTTCTTCAG

>Exon 2

GGCAGCACAAGAGCAAGGAGTTCTTGCAGATCAACAGCCTGGGGAAACTGCCGACGCTCAAGGATGGTGATTTCATCTTGACCGAAAG

>Intron 2

ATGCCCTCCTTCCCTCACCCCTCACCGCATCCGGAGCCCATGTGACCTTGGCTCTCCCCACTGGCCCCGGGCCCCAATGGCCCTCCCATACCCCATGGGGCAGCGAGGGAGGGGAAAGGCGAGGGATCTGGCCGGGCGCGGTGGCTCACGCCTGTCATCCCGGCACTTCGGGAGGCCAAGGCGGGCGGATCACGAGGTCAGGAGACCGAGACCATCCTGGCTAACACCGTGAAACACCGTCTCTACTAAAAATGGAAAAAAAAATTAACCGGGCGTGGTGGCGGGCGTCTGTAGTCCCAGCTACTCTGGAGGCTGAGGCAGGAGAATGGTGTGAACCCAGGAGGCGGAGCTTGCAGTGTGCTGAGATCGCGCCACTGCACTCCAGCCTGGGCGACAGAGCTAGACTCCGTCTCAAAAAGGCCGGGCGCGGTGGTTTACGCCTGTAATCCCAGCACTTTGGGAGGCCGAGGTGGGCGGATCACGAGGTCAGGAGATCGAGACCATCCTGGCTAACACGGTGAAACCCTGTCTCTACTAAAAATACAAAACATTAGCAGGGCGTGGTGGCGGCCGCCTGTAGTCCCAGCTACTGGGGAGGCTGAGGCAGGAGAATGGCGTGAACTCAGGAGGCGGAGCTTGCAATGAGCAGAGGTCGCGCCACTGCACTCCAGCCTGGGTGACAGAGGGAGCCCACTCCAGCCTGGGCGACAGAGGGAGACTCCGTCTCAAAAAAAAAGGAAAGAAAGAAAGGAGAGGTATCTGGGGAGAAGGTACAGCTTGGGGTGTGTCCGGGATGAGCAGGGGCTGACAGAACATGTCCCCCCACCTCTCATCTTCAGCCTTTTCTGAGCCGCAGGGCCTCTCCACTCCCAGACTGAAGGGTATTAGAAGAGAAGACAAGGGAACATTTTTCCACTGTTGCGCATTTGTTCAACAAATGCTAGCTGAAAAGAGCCTCTAGTGACTTGTCGCAGACTACCCAATCTACCCAGGCCGGGCCTAGAGGCCAATGCCATGGCCCAAGGGCACAGCTCATGGTGAGGTCCAGCTGCTGGGCAGGAAAAGGACAAGAGGTCAGGTGGCTGCAGAGGTGATGGCTGGGGGCCTGTCAGACGGGGGCCAAAGACATTCCTCCCCTCGTGATCCCTGACCCAAGCGCGTGGACATGCAAGGGACTCCACGGAGCATCCACTGTGTGCCAGCCCCATGCAGGGTTCCAGGGGTCCAGGGAGCCTATTCTGAGCTGCACCGCCTCGGACAAGTCACTTGACCATTCTGACCTTGAGTTTTCTCTTGTGCTAAAAGGCTAACAGGAGTGTCTACCTCACAGGGCGGCTGCTGGCATATCACAGAGATGAGGTTCTCAAAATGCAAAGCAGAAGGTCCAGCCAAGAGTCGGTGCCCAAGGCAACAAAGACAGGAGGAGACTCGTAGGAGGAGGGGGTGGTGTTGGGGAGCTGGAGATGGAGGGCGAGGCTGGAGGGCAGTCCTTCAAATGCAGAGAAGCCCCCGGGCCCCACTGGCAGATGGGAGCAGTTAGGGGTAAATGCCTGGTGCCAGTGTCCTTATAGCCACTGCCCATTTGTTCCCAG

>Exon 3

CTCGGCCATCCTGATTTACCTGAGCTGTAAGTACCAGACGCCGGACCACTGGTATCCATCTGACCTGCAGGCTCGTGCCCGTGTTCATGAGTACCTGGGCTGGCATGCCGACTGCATCCGTGGCACCTTTGGTATACCCCTGTGGGTCCAG

>Intron 3

GTGAGGAGAGCCATCTGGAGAGTGATTGGCCATCAGGGAGTAGTTGGCAGTAGGCCGGGGCCATAGACTGACCCACTCTCTGCCCCCATCAG

>Exon 4

ATGTTGGGGCCACTCATTGGGGTCCAGGTGCCCAAGGAGAAGGTGGAACGCAACAGGACTGCCATGGACCAGGCCCTGCAATGGCTGGAGGACGAGTTCCTGGGGGACAGGCCCTTCCTCGCTGGCCAGCAGGTGACACTGGCTGATCTCATGGCCCTGGAGGAGCTGATGCAG

>Intron 4

GTGTGAGCTCAGCCTGTGGGCAGTGTCCCTCTTCGTGTCACACCCATGAGGCAGACAGAAACACTGAGGCCTGGAGAAAGCCAGAACTTTGCCCAGAATCATAGAGCAAGTCTCTGGATGATCTGGGGCCAGAACCCTGAACTTCTGCCTCCTGCCTGGGTGTGGGGTCTCACCCTGGCTGCTCTTGGGCTCTAAGGCTGAACATACTGCCTGGGCCCCTGTGGTCCATTCACTTAGGGGCTGGGGAATGGACCATGTCTCTGATACTTCTGCCCATGGTTCCAGCATTCGGGTCGGCAGTGACAACTGGGAAAGTTGTATGCCCACAACTTTTTCATCCTTGTCCCTACAG

>Exon 5

CCGGTGGCTCTCGGCTATGAACTGTTTGAGGGACGGCCACGACTGGCAGCATGGCGTGGATGAGTGGAGGCTTTCCTGGGTGCTGAGCTATGCCAGGAGGCCCACAGCATCATCTTGAGCATCCTGGAACAGGCGGCCAAGAAAACCCTCCCAACACCCTCACCAGAGGCCTATCAGGCTATGCTGCTTCGAATCGCCAGGATCCCCTGA

>Post

AGGGTCTGGGATGGGGGCCAGGAGATTAGCAACAAGGATTCATTCTGTTACTTACTTGCCCCTTTTTATCTTTCCCTCTTGCCCCAGTCCCTTCTCTCCAGCTTCATGTGAAGCTCTGCACAGACAAGACACTCAGTGTCCTTGGCAGTGCTGCTACTCCTCAGGTGCAGCATACATAACCAGTAAGAGACTAAATCTGCAATATATAAAGAGCTCCTACAAATCAGTAACATGAAGAACACTCAAAAATTGGCAAATGTCATCAGTGTTTTAAACAGAATAAAGATTCCAAACACTTTGAATAGAGAACCAAGAGTTATTGGTTTTACTACATTGTTGTGTTATACATATGGAGTAAAAGTATGTGCTAGTAATCCTCATCATGGTTAATAACAAAGTAACCTCACAATAACGAGTCAACATAATTGTATCACCAGGGCAACAAAATGTTAAGTAAGTAACCAATTCGAATTGCAAACTGTTAAAGGATATAGGCGATGTTTCACAGGGCATAGCAACGGTCTTTGAAGTCTAGGAA

**hg19-1 (AVA)**

>Promoter

CCGTGAGAGTAACTGGGATTGCAGGTGTGGGCCAGCATGCCCTGTTATCACTCCCTTTCTCTGAGTCAGCTTTTCTCTCTCAGTCTCGTCTACTCCTCGAACTCAGGAAATGTCACTACAGGTGGCCCCAGCTCCCGTTTACCTCCCCATAGGGAGCTCCTCTCTCCAGTTCCAGTTTCAAAACTCCCAAGGAAGCATTCTGGTTCACTCGCTTGGGCCACTGGCCAGAGGGATGGGATACTCTGAAAGATTCAGCTAGAGTCCCGGGCCCAGCCCTGGACCATCACTGTGCCCCCTGGTGAGATGCCAGGGCTGGGATTCAGGGAGAAGAAAGGAGGTTCCCGGACAGTCATTCCTGCCTCCCGCGGCTGCGGGCTCCCTGCCCCCATCCTGTGCACGAAGTGGGAGCTCCCGCTGTCTGGCAGCTCCCGCTGTCTGGCAGCAGCTGCTCTGCAGGGGACAGTCTGGACGGCAGAAAGTTCATCCTTAACCCCAGCCTTCCAGTCAAGGTTCCCACCAGTTTGGGACACCTGCAAGTGTCACATCCCACTGGGTGAAACTCTAAGATCCCTTTTAGGGGATCCCATTCGCTCCCTCCCTTCCGCCACCATGCAGCGCCGAGAAACAGAGCTCTGAACGAACCCTCAGATGTCCGTGCGCTGGGGCCTTTCCAGGACGGCGGCGCCCAGTCGTTTCTGGGTCAGGGCGACGCCTGGAACTGGGCAGGGTCCCTGGCACCGGGATCCCGAAAAGCAGACCTGCTTCTCCCTGTCCAGCCGGTTCCCCTTCCCCTTGCAGTCGGCCCCCTGCATCCGCGTCCTCCCTGCCAGTCGAGGGTCCCCAGCTCCAACTCCACCCTCCCAGCTGTGCGTTCATAGCGACCGCCCTCCCTGTAGGGACGCACGGATCTGGTGGTGGAGTCTTGGCCGGCAGGACTGGACAGGAACCGAAGGGGCGAGGCGGGTCCGGGGGTGGTGCGCTCCAATTGGGTGCTGTCCCCAGGGGGTGGGGCCTGATCCCCTATTTCCCGGCGCGCCGG

>Exon 1

GATCCTGCCACAGCTGCTGCCCACACCGCGCTCAGCGCCTTCACTGCCATCCCCGCTGTCCTTGCCGCCCCCGCCATGGGCCTAGAGCTGTTTCTTGACCTGGTGTCCCAGCCCAGCCGCGCCGTCTACATCTTCGCCAAGAAGAATGGCATCCCCTTAGAGCTGCGCACCGTGGATTTGGTCAAAG

>Intron 1

GTGGGCCCAGCCCGTTTCCCCGCGTGTCCACAAACCCAGTGCACCCCCAGGCCCCCGCCCTGCTCTGCCCTGAGCGTCTCGCCGCCCGCACAGCCCCCTCACCTCCTCCTGCAGCGTCTGCCACCAGAGAATGCTGTGGACTGAGTGGCCTTGAAGGGATCACAGCCTCTCTGAACCTTAGCTTGCCTTCTGAAAAGGAGGATAACGTTACCTTCTGCTCTGTAGGGATGGAAAGAAAATACTGAATGGAGTTGACAGAGTTCTTGCGTGGAATGCACGCATATAAATTCACAAAGCCCAGAAGACCTCGGGAAGAAGGACATACTGTTGTGAGAATTAAGAGATGGGAAGAGATGAGCCACCCCAGTTTGCCTCCCCTCCCCTGGCCCACCAGAGTCCGGCTAGAAAACTTCTCTTTATCCACCTGCTGCACCTGGCCCCACCCACCAAAACCCCCCAGCTGCCCCGGAATGTGGCAGGGCAGGGAGGCCCAGCCAGGGAGTGAGGCTGATCCAGGCCTCTAGTCCCAGACCTTGCTGTTTCTCAGGGCTGTGGGGCTCCGCTTGGGGAGGAGGAGGGAGGGTGTAGAGGTGCAGCGTTTTTATTCTGAAGACCTTTTCTGACTTCTTCCTCTTCAG

>Exon 2

GGCAGCACAAGAGCAAGGAGTTCTTGCAGATCAACAGCCTGGGGAAACTGCCGACGCTCAAGGATGGTGATTTCATCTTGACTGAAAG

>Intron 2

GTGCCCTCCTTCCCTCACCCCTCACCGCATCCGGAGCCCATGTGACCTTGGCTCTCCCCACTGGCCCCGGGCCCCAGTGGCCCTCCCATACCCCATGGGGCAGCGAGGGAGGGGAAAGGCGAGGGATCTGGCCGGGCGCGGTGGCTCACGCCTGTCATCCCGGCACTTCGGGAGGCCAAGGCGGGCGGATCACGAGGTCAGGAGACCGAGACCATCCTGGCTAACACCGTGAAACACCGTCTCTACTAAAAATGGAAAAAAA-GTTAACCGGGCGTGGTGGCGGGCGCCTGTAGTCCCAGCTACTCTGGAGGCTGAGGCAGGAGAATGGTGTGAACCCAGGAGGCGGAGCTTGCAGTGTGCTGAGATCGCGCCACTGCACTCCAGCCTGGGCGACAGAGCTAGACTCCGTCTCAAAAAGGCCGGGCGCGGTGGTTTACGCCTGTAATCCCAGCACTTTGGGAGGCCGAGGTGGGCGGATCACGAGGTCAGGAGATCGAGACCATCCTGGGTAACACGGTGAAACCCTGTCTCTACTAAAAATACAAAACATTAGCAGGGCGTGGTGGCGGCCGCCTGTAGTCCCAGCTACTGGGGAGGCTGAGGCAGGAGAATGGCGTGAACCCAGGAGGCGGAGCTTGCAATGAGCAGAGATAGCACCACTGCACTCCAGCCTGGGTGACAGAGGGAGCCCACTCCAGCCTGGGCGACAGAGGGAGACTCCGTCTCAAAAAAAAAGGAAAGAAAGAAAGGAGAGGTATCTGGGGAGAAGGTACAGCTTGGGGTGTGACCGGGATGAGCAGGGGCTGACAGAACATGTCCCCCCACCTCTCATCTTCCAGCCTTTTCTGAGCCGCAGGGCCTCTCCACTCCCAGACTGAAGGGTATTAGAAGAGAAGACAAGGGAACATTTTTCCACTGTTGCGCATTTGTTCAACAAATGCTAGCTGAAAAGAGCCTCTAGTGACTTGTCGCAGACTACCCAATCTACCCAGGCCGGGCCTAGAGGCCAATGCCATGGCCCAAGGGCACAGCTCATGGTGAGGTCCAGCTGCTGGGCAGGAAAAGGACAAGAGGTCAGGTGGCTGCAGAAGTGATGGCTGGGGGCCTGTCAGACGGGGGCCAAAGACATTCCTCCCCTCGTGATCCCTGACCCAAGCGCGTGGACATGCAAGGGACTCCACGGAGCATCCACTGTGTGCCAGCCCCATGCAGGGTTCCAGGGGTCCAGGGAGCCTATTCTGAGCTGCACCGCCTCGGACAAGTCACTTGACCATTCTGACCTTGAGTTTTCTCTTGTGCTAAAAGGCTAACAGGAGTGTCTACCTCACAGGGCGGCTGCTGGCATATCACAGAGATGAGGTTCTCAAAATGCAAAGCAGAAGGTCCAGCCAAGAGTCGGTGCCCAAGGCAACAAAGACAGGAGGAGACTCGTAGGAGGAGGGGGTGGTGTTGGGGAGCTGGAGATGGAGGGCGAGGCTGGAGGGCAGTCCTTCAAATGCAGAGAAGCCCCCGGGCCCCACTGGCAGATGGGAGCAGTTAGGGGTAAATGCCTGGTGCCAGTGTCCTTATAGCCACTGCCCATTTGTTCCCAG

>Exon 3

CTCGGCCATCCTGATTTACCTGAGCTGTAAGTACCAGACGCCGGACCACTGGTATCCATCTGACCTGCAGGCTCGTGCCCGTGTTCATGAGTACCTGGGCTGGCATGCCGACTGCATCCGTGGCACCTTTGGTATACCCCTGTGGGTCCAG

>Intron 3

GTGAGGAGAGCCATCTGGAGAGTGATTGGCCATCAGGGAGTAGTTGGCAGTAGGCCGGGGCCATAGACTGACCCACTCTCTGCCCCCATCAG

>Exon 4

GTGTTGGGGCCCCTCATTGGGGTCCAGGTGCCCGAGGAGAAGGTGGAACGCAACAGGACTGCCATGGACCAGGCCCTGCAATGGCTGGAGGACAAGTTCCTGGGGGACAGGCCCTTCCTCGCTGGCCAGCAGGTGACACTGGCTGATCTCATGGCCCTGGAGGAGCTGATGCAG

>Intron 4

GTGTGAGCTCAGCCTGTGGGCAGTGTCCCTCTTCGTGTCACACCCATGAGGCAGACAGAAACACTGAGGCCTGGAGAAAGCCAGAACTTTGCCCAGAATCATAGAGCAAGTCTCTGGATGATCTGGGGCCAGAACCCTGAACTTCTGCCTCCTGCCTGGGTGTGGGGTCTCACCCTGGCTGCTCTTGGGCTCTAAGGCTGAACATACTGCCTGGGCCCCTGTGGTCCATTCACTTAGGGGCTGGGGAATGGACCATGTCTCTGATACTTCTGCCCATGGTTCCAGCATTCGGGTCGGCAGTGACAACTGGGAAAGTTGTATGCCCACAACTTTTTCATCCTTGTCCCTACAG

>Exon 5

CCGGTGGCTCTCGGCTATGAACTGTTTGAGGGACGGCCACGACTGGCAGCATGGCGTGGACGAGTGGAGGCTTTCCTGGGTGCTGAGCTATGCCAGGAGGCCCACAGCATCATCTTGAGCATCCTGGAACAGGCGGCCAAGAAAACCCTCCCAACACCCTCACCAGAGGCCTATCAGGCTATGCTGCTTCGAATCGCCAGGATCCCCTGA

>Post

AGGGTCTGGGATGGGGGCCAGGAGATTAGCAACAAGGATTCATTCTGTTACTTACTTGCCCCTTTTTATCTTTCCCTCTTGCCCCAGTCCCTTCTCTCCAGCTTCATGTGAAGCTCTGCACAGACAAGACACTCAGTGTCCTTGGCAGTGCTGCTACTCCTCAGGTGCAGCATACATAACCAGTAAGAGACTAAATCTGCAATATATAAAGAGCTCCTACAAATCAGTAACATGAAGAACACTCAAAAATTGGCAAATGTCATCAGTGTTTTAAACAGAATAAAGATTCCAAACACTTTGAATAGAGAACCAAGAGTTATTGGTTTTACTACATTGTTGTGTTATACATATGGAGTAAAAGTATGTGCTAGTAATCCTCATCATGGTTAATAACAAAGTAACCTCACAATAACGAGTCAACATAATTGTATCACCAGGGCAACAAAATGTTAAGTAAGTAACCAATTCGAATTGCAAACTGTTAAAGGATATAGGCGATGTTTCACAGGGCATAGCAACGGTCTTTGAAGTCTAGGAA

**hg19-2 (NO AVA)**

>Promoter

CCGTGAGAGTAACTGGGATTGCAGGTGTGGGCCAGCATGCCCTGTTATCACTCCCTTTCTCTGAGTCAGCTTTTCTCTCTCAGTCTCGTCTACTCCTCGAACTCAGGAAATGTCACTACAGGTGGCCCCAGCTCCCGTTTACCTCCCCATAGGGAGCTCCTCTCTCCAGTTCCAGTTTCAAAACTCCCAAGGAAGCATTCTGGTTCACTCGCTTGGGCCACTGGCCAGAGGGATGGGATACTCTGAAAGATTCAGCTAGAGTCCCGGGCCCAGCCCTGGACCATCACTGTGCCCCCTGGTGAGATGCCAGGGCTGGGATTCAGGGAGAAGAAAGGAGGTTCCCGGACAGTCATTCCTGCCTCCCGCGGCTGCGGGCTCCCTGCCCCCATCCTGTGCACGAAGTGGGAGCTCCCGCTGTCTGGCAGCTCCCGCTGTCTGGCAGCAGCTGCTCTGCAGGGGACAGTCTGGACGGCAGAAAGTTCATCCTTAACCCCAGCCTTCCAGTCAAGGTTCCCACCAGTTTGGGACACCTGCAAGTGTCACATCCCACTGGGTGAAACTCTAAGATCCCTTTTAGGGGATCCCATTCGCTCCCTCCCTTCCGCCACCATGCAGCGCCGAGAAACAGAGCTCTGAACGAACCCTCAGATGTCCGTGCGCTGGGGCCTTTCCAGGACGGCGGCGCCCAGTCGTTTCTGGGTCAGGGCGACGCCTGGAACTGGGCAGGGTCCCTGGCACCGGGATCCCGAAAAGCAGACCTGCTTCTCCCTGTCCAGCCGGTTCCCCTTCCCCTTGCAGTCGGCCCCCTGCATCCGCGTCCTCCCTGCCAGTCGAGGGTCCCCAGCTCCAACTCCACCCTCCCAGCTGTGCGTTCATAGCGACCGCCCTCCCTGTAGGGACGCACGGATCTGGTGGTGGAGTCTTGGCCGGCAGGACTGGACAGGAACCGAAGGGGCGAGGCGGGTCCGGGGGTGGTGCGCTCCAATTGGGTGCTGTCCCCAGGGGGTGGGGCCTGATCCCCTATTTCCCGGCGCGCCGG

>Exon 1

GATCCTGCCACAGCTGCTGCCCACACCGCGCTCAGCGCCTTCACTGCCATCCCCGCTGTCCTTGCCGCCCCCGCCATGGGCCTAGAGCTGTTTCTTGACCTGGTGTCCCAGCCCAGCCGCGCCGTCTACATCTTCGCCAAGAAGAATGGCATCCCCTTAGAGCTGCGCACCGTGGATTTGGTCAAAG

>Intron 1

GTGGGCCCAGCCCGTTTCCCCGCGTGTCCACAAACCCAGTGCACCCCCAGGCCCCCGCCCTGCTCTGCCCTGAGCGTCTCGCCGCCCGCACAGCCCCCTCACCTCCTCCTGCAGCGTCTGCCACCAGAGAATGCTGTGGACTGAGTGGCCTTGAAGGGATCACAGCCTCTCTGAACCTTAGCTTGCCTTCTGAAAAGGAGGATAACGTTACCTTCTGCTCTGTAGGGATGGAAAGAAAATACTGAATGGAGTTGACAGAGTTCTTGCGTGGAATGCACGCATATAAATTCACAAAGCCCAGAAGACCTCGGGAAGAAGGACATACTGTTGTGAGAATTAAGAGATGGGAAGAGATGAGCCACCCCAGTTTGCCTCCCCTCCCCTGGCCCACCAGAGTCCGGCTAGAAAACTTCTCTTTATCCACCTGCTGCACCTGGCCCCACCCACCAAAACCCCCCAGCTGCCCCGGAATGTGGCAGGGCAGGGAGGCCCAGCCAGGGAGTGAGGCTGATCCAGGCCTCTAGTCCCAGACCTTGCTGTTTCTCAGGGCTGTGGGGCTCCGCTTGGGGAGGAGGAGGGAGGGTGTAGAGGTGCAGCGTTTTTACTCTGAAGACCTTTTCTGACTTCTTCCTCTTCAG

>Exon 2

GGCAGCACAAGAGCAAGGAGTTCTTGCAGATCAACAGCCTGGGGAAACTGCCGACGCTCAAGGATGGTGATTTCATCTTGACCGAAAG

>Intron 2

GTGCCCTCCTTCCCTCACCCCTCACCGCATCCGGAGCCCATGTGACCTTGGCTCTCCCCACTGGCCCCGGGCCCCAGTGGCCCTCCCATACCCCATGGGGCAGCGAGGGAGGGGAAAGGCGAGGGATCTGGCCGGGCGCGGTGGCTCACGCCTGTCATCCCGGCACTTCGGGAGGCCAAGGCGGGCGGATCACGAGGTCAGGAGACCGAGACCATCCTGGCTAACACCGTGAAACACCGTCTCTACTAAAAATGGAAAAAAAAGTTAACCGGGCGTGGTGGCGGGCGCCTGTAGTCCCAGCTACTCTGGAGGCTGAGGCAGGAGAATGGTGTGAACCCAGGAGGCGGAGCTTGCAGTGTGCTGAGATCGCGCCACTGCACTCCAGCCTGGGCGACAGAGCTAGACTCCGTCTCAAAAAGGCCGGGCGCGGTGGCTCACGCCTGTAATCCCAGCACTTTGGGAGGCCGAGGTGGGCGGATCACGAGGTCAGGAGATCGAGACCATCCTGGCTAACACGGTGAAACCCTGTCTCTACTAAAAATACAAAACATTAGCAGGGCGTGGTGGCGGCCGCCTGTAGTCCCAGCTACTGGGGAGGCTGAGGCAGGAGAATGGCGTGAACTCAGGAGGCGGAGCTTGCAATGAGCAGAGGTCGCGCCACTGCACTCCAGCCTGGGTGACAGAGGGAGCCCACTCCAGCCTGGGCGACAGAGGGAGACTCCGTCTCAAAAAAAAAGGAAAGAAAGAAAGGAGAGGTATCTGGGGAGAAGGTACAGCTTGGGGTGTGTCCGGGATGAGCAGGGGCTGACAGAACATGTCCCCCCACCTCTCATCTT-CAGCCTTTTCTGAGCCGCAGGGCCTCTCCACTCCCAGACTGAAGGGTATTAGAAGAGAAGACAAGGGAACATTTTTCCACTGTTGCGCATTTGTTCAACAAATGCTAGCTGAAAAGAGCCTCTAGTGACTTGTCGCAGACTACCCAATCTACCCAGGCCGGGCCTAGAGGCCAATGCCATGGCCCAAGGGCACAGCTCATGGTGAGGTCCAGCTGCTGGGCAGGAAAAGGACAAGAGGTCAGGTGGCTGCAGAGGTGATGGCTGGGGGCCTGTCAGACGGGGGCCAAAGACATTCCTCCCCTCGTGATCCCTGACCCAAGCGCGTGGACATGCAAGGGACTCCACGGAGCATCCACTGTGTGCCAGCCCCATGCAGGGTTCCAGGGGTCCAGGGAGCCTATTCTGAGCTGCACCGCCTCGGACAAGTCACTTGACCATTCTGACCTTGAGTTTTCTCTTGTGCTAAAAGGCTAACAGGAGTGTCTACCTCACAGGGCGGCTGCTGGCATATCACAGAGATGAGGTTCTCAAAATGCAAAGCAGAAGGTCCAGCCAAGAGTCGGTGCCCAAGGCAACAAAGACAGGAGGAGACTCGTAGGAGGAGGGGGTGGTGTTGGGGAGCTGGAGATGGAGGGCGAGGCTGGAGGGCAGTCCTTCAAATGCAGAGAAGCCCCCGGGCCCCACTGGCAGATGGGAGCAGTTAGGGGTAAATGCCTGGTGCCAGTGTCCTTATAGCCACTGCCCATTTGTTCCCAG

>Exon 3

CTCGGCCATCCTGATTTACCTGAGCTGTAAGTACCAGACGCCGGACCACTGGTATCCATCTGACCTGCAGGCTCGTGCCCGTGTTCATGAGTACCTGGGCTGGCATGCCGACTGCATCCGTGGCACCTTTGGTATACCCCTGTGGGTCCAG

>Intron 3

GTGAGGAGAGCCATCTGGAGAGTGATTGGCCATCAGGGAGTAGTTGGCAGTAGGCCGGGGCCATAGACTGACCCACTCTCTGCCCCCATCAG

>Exon 4

GTGTTGGGGCCACTCATTGGGGTCCAGGTGCCCAAGGAGAAGGTGGAACGCAACAGGACTGCCATGGACCAGGCCCTGCAATGGCTGGAGGACAAGTTCCTGGGGGACAGGCCCTTCCTCGCTGGCCAGCAGGTGACACTGGCTGATCTCATGGCCCTGGAGGAGCTGATGCAG

>Intron 4

GTGTGAGCTCAGCCTGTGGGCAGTGTCCCTCTTCGTGTCACACCCATGAGGCAGACAGAAACACTGAGGACTGGAGAAAGCCAGAACTTTGCCCAGAATCATAGAGCAAGTCTCTGGATGATCTGGGGCCAGAACCCTGAACTTCTGCCTCCTGCCTGGGTGTGGGGTCTCACCCTGGCTGCTCTTGGGCTCTAAGGCTGAACATACTGCCTGGGCCCCTGTGGTCCATTCACTTAGGGGCTGGGGAATGGACCATGTCTCTGATACTTCTGCCCATGGTTCCAGCATTCGGGTCGGCAGTGACAACTGGGAAAGTTGTATGCCCACAACTTTTTCATCCTTGTCCCTACAG

>Exon 5

CCGGTGGCTCTCGGCTATGAACTGTTTGAGGGACGGCCACGACTGGCAGCATGGCGTGGACGAGTGGAGGCTTTCCTGGGTGCTGAGCTATGCCAGGAGGCCCACAGCATCATCTTGAGCATCCTGGAACAGGCGGCCAAGAAAACCCTCCCAACACCCTCACCAGAGGCCTATCAGGCTATGCTGCTTCGAATCGCCAGGATCCCCTGA

>Post

AGGGTCTGGGATGGGGGCCAGGAGATTAGCAACAAGGATTCATTCTGTTACTTACTTGCCCCTTTTTATCTTTCCCTCTTGCCCCAGTCCCTTCTCTCCAGCTTCATGTGAAGCTCTGCACAGACAAGACACTCAGTGTCCTTGGCAGTGCTGCTACTCCTCAGGTGCAGCATACATAACCAGTAAGAGACTAAATCTGCAATATATAAAGAGCTCCTACAAATCAGTAACATGAAGAACACTCAAAAATTGGCAAATGTCATCAGTGTTTTAAACAGAATAAAGATTCCAAACACTTTGAATAGAGAACCAAGAGTTATTGGTTTTACTACATTGTTGTGTTATACATATGGAGTAAAAGTATGTGCTAGTAATCCTCATCATGGTTAATAACAAAGTAACCTCACAATAACGAGTCAACATAATTGTATCACCAGGGCAACAAAATGTTAAGTAAGTAACCAATTCGAATTGCAAACTGTTAAAGGATATAGGCGATGTTTCACAGGGCATAGCAACGGTCTTTGAAGTCTAGGAA

**Accession No. JN819433 - Chimpanzee Fibroblast - 2 (AVA)**

>Promoter

CCGTGAGAGTAACTGGGATTGCAGGTGTGGGCCAGCATGCCCTGTTATCACTCCCTTTCTCTGAGTCAGCTTTTCTCTCCCAGACTCGTCTACTCCTTGAACTCAGGAAATGTCACTACAGGTGGCCCCAGCTCCCATTTACCTCCCCATAGGGAGCTCCTCTCTCCAGTTCCAATTTCAAAACTCTCAAGGAAGCATTCTGGTTCACTCGCTTGGGCCACTGGCCAGAGGGATGGGATACTCTGAAAGATTCAGCTAGAGTCCCGGGCCCAGCCCTGGACCATCACTGTGCCCCCTGGTGAGATGCCAGGGCTGGGATTCAGGGAGAAGAAAGGAGGTTCCCGGACAGTCATTCCTGCCTCCCGCGGCTGCGGGCTCCCTGCCCCCATCCTGTGCACGAAGTGGGAGCTCCCGCTGTCTGGCAGCAGCTGCTCTGCAGGGGACAGTCTGGACGGTAGAAAGTTCATCCTTAACCCCAGCCTTCCAGTCAAGGTTCCCACCAGTTTGGGACACCTGCAAGTGTCACATCCCACTGGGTGAAACTCTAAGATCCCTTTTAGAGGATCCCATTCGCTCCCTCCCTTCCGCCACCATGCAGCGCCGAGAAACAGAGCTCTGAACGAACCCTCAGATGCCCGTGCGCTGGGGCCTTTCCAGGACGGCGGCGCCCAGTCGTTTCTGGGTCGGGGCGACGCCTGGAACTGGGCAGGGTCCCTGGCACCGGGATCCCGAAAAGCAGACCTGCTTCTCCCTGTCCAGCCGGTTCCCCTTCCCCTTGCTGTCGGCCCCCTGCATCCGCGTCCTCCCTGCCAGTAGAGGGCCCCCAGCTCCAACTCCACCCTCCCAGCTGTGCGTGGATAGCGACCGCCCTCCCTGTAGGGACGCACGGACCTGGTGGTGGAGTCTTGGCCGGCAGGACTGGACAGAAACGGAAGGGGCGAGGCGGGTCCGGGGGTGGTGCGCTCCAATTGGGTGCTGTCCCCAGGGGGTGGGGCCTGATCCCCTATTTCCCGGCGCGCCGG

>Exon 1

GATCCTGCCACAGCTGCTGCCCACACCGCGCTCAGCGCCTTCACTACCTTCCCCGCTGTCCTTGCCGCCCCCGCCATGGGCCTAGAGCTGTTTCTTGACCTGGTGTCCCAGCCCAGCCGCGCCGTCTACATCTTCGCCAAGAAGAATGGCATCCCCTTAGAGCTGCGCACCGTGGATCTTATCAAAG

>Intron 1

GTGGGCCCAGCCCGTTTCCCCGCGTGTCCACAAACCCAGTGGGCCCCCAGGCCCCCGCCCTGCTCTGCCCTGAGCGTCTCGCCGCCCGCACAGCCCCCTCACCTCCTCCTGCAGCGTCTGCCACCAGAGAATGCTGTGGACTGAGTGGCCTTGGAGGGATCACAGCCTCTCTGAACCTTAGCTTGCCTTCTGAAAAGGAGGATAAGGTTACCTTCTGCTCTGTAGGGATGGAAAGAAAATACTGAATGGAGTTTACAGAGTTCTTGCGTGGAATGCACGCATATAAATTCACAAAGCCCAGAAGACCTTGGGAAGAAGGACATGCTGTTGTGAGAATTAAGAGATGGGAAGAGATGAGCCACCCCAGTTTGCCTCCCCTCCCCTGGCCCACCAGAGTCCGGCTAGAAAACTTCTCTTTATCCACCTGCTGCACCTGGCCCCACCCACCAAAGCCCCCCAGCTGCCCCGGAATGTGGCAGGGCAGGGAGGCCCAGCCAGGGATTGAGGCTGATCCAGGCCTCTAGTCCCAGACCTTGCTGTTTCTCAGGGCTGTGGGGCTCCGCTTGAGGAGGGGGAGGGAGGGTGTAGAGGTGCAGCGTTTTTACTCTGAAGACCTTTTGTGACTTCTTCCTCTTCAG

>Exon 2

GGCAGCACAAGAGCAAGGAGTTCTTGCAGATCAACAGCCTGGGGAAACTGCCGACGCTCAAGGATGGTGATTTCATCTTGACCGAAAG

>Intron 2

GTGCCCTCCTTCCCTCACCCCTCACCGCATCCGGAGCCCCTGTGACCCTGGCTCTCCCCACTGGCCCCCGGGCCCCACTGGCCCTCCCATACCCCATGGGGCAGCGAGGGAGGGGAAAGGCGAGGGATCTGGCCGGGCGCGGTGGCTCACGCCTGTCATCCCAGCACTTCGGGAGGCCAAGGCGGGCGGATCACGAGGTCAGGAGACCGAGACCATCCTGGCTAACACCGTGAAACACCGTCTCTACTAAAAATGGAAAAAAAAATTAACCGGGCGTGGTGGCGGGCGTCTGTAGTCCCAGCTACTCGGGAGGCTGAGGCAGGAGAATGGCGTGAACCCAGGAGGCGGAGCTTGCGGTGAGCTGAGATCGCGCCACTGCACTCCATCCTGGGCGACAGAGCTAGACTCCGTCTCAAAAAGGTCGGGCGCGGTGTCTCACGCCTGTAATCCCAGCACTTTGGGAGGCCGAGGTGGGCGGATCACGAGGTCAGGAGATCGAGACCATCCTGGCTAACACGGTGAAACCCCGTCTGTACTAAAAATACAAAACATTAGCAGGGCGTGGTGGCGGGCGCCTGTAGTCCCAGCTACTCCAGAGGCTGAGGCAGGAGAATGGCGTGAACCGGGGAGGCGGAGCTTGCAGTGAGCTCGCGCCACTACACTCCAGCCTGGGCGAGAGAGGGAGACTCCGTCTCAAAAAAAAAAAAAAAGAAAGAAAGAAAAGAAAAAAAATAAAGGAGAGGGATCTGGGGAGAAGGTACAGCTTGGGGTGTGTCCGGGATGAGCAGGGGCTGACAGAACATGTCCCCGCACCTCTCATCTTCAGCCTTTTCTGAGCCGCAGGGCCTCTCCACTCACAGACTGAAGGCTATTAGAAGAGAAGACAAGGGAACATTTTTCCACTGTTGCGCATTTGTTCAACAAATGCTAGCTGAAAAGAGCCTCTAGTGACTTGTCGCAGACTACCCAATCTACCCAGGCCAGGCCTAGAGGCCAATGCCATGGCCCAAGGGCACAGCTCATGGTGAGGTCCAGCTGCTGGGCAGGAAAAGGACAAGAGGTCAGGTGGCTGCAGAAGTGATGGCTGGGGGCCTGTCAGACGGGGGCCAAAGACATTCCTCCCCTCGTGATCCCTGACCCAAGCGCGTGGACATGCAAGGGACTCCACGGAGCATCCACTGTGTGCCAGCCCCATGCAGGGTTCCAGGGGTCCAGGGAGCCTATTCTGAGCTGCACCGCCTCGGACAAGTCACTTGACCATTTCTGACCTTGAGTTTTCTCTTGTGCTAAAAGGCTAACAGGAGTCTCTACCTCACAGGGCGGCTGCTGGCATATCACAGAGATGAGGGTCTCAAAGTGCAAAGCAGAAGGTCCAGCCAAGAGTCGGTGCCCAAGGCAACAAAGACAGGAGGAGACTCGTAGGAGGAGGGGGTGGTGTTGGGGAGCTGGAGCTGGAGGGCGAGGCTGGAGGGCAGTCCTTCAAATGCAGAGAAGCCCCCGGGCCCCACTGGCAGATGGGAGCAGTTAGGGGTAAATGCCTGGTGCCAGTGTCCTTATAGCGACTGCCCATTTGTTCCCAG

>Exon 3

CTCGGCCATCCTGATTTACCTGAGCTGTAAGTACCAGACGCCGGACCACTGGTATCCATCTGACCTGCAGGCTCGTGCCCGTGTTCATGAGTACCTGGGCTGGCATGCCGACTGCATCCGTGGCACCTTTGGTATACCTCTGTGGGTCCAG

>Intron 3

GTGAGGAGCGCCATCTGGAGAGTGATTGGCCATCAGAAAGTAGTTGGCAGTAGGCCGGGGCCATAGACTGACCCACTCTCTGCCCCCATCAG

>Exon 4

GTGTTGGGGCCACTCATTGGGGTCCAGGTGCCCGAGGAGAAGGTGGAACGCAACAGGACTGCCATAGACCAGGCCCTGCAATGGCTGGAGGACAAGTTCCTGGGGGACAGGCCCTTCCTTGCTGGCCAGCAGGTGACACTGGCTGATCTCATGGCTCTGGAGGAGCTGATGCAG

> Intron 4

GTGTGAGCTCAGCCTGTGGGCAATGTCCCTCTTCGTGTCACACCCATGAGGCAGACAGACACACTGAGGCCTGGAGAAAGCCAGAACTTTGCCCAGAATCATAGAGCAAGTCTCTGGATGATCTGGGGCCAGAACCCTGAACTTCTGCCTCCTGCCTGGGTGTGGGGTCTCACCCTGGCTGCTCTTGGGCTCTAAGGCTGAACATACTGCCTGGGCCGCTGTGGTCCATTCACTTAGGGGCTGGGGAATGGACCATGTCTCTGATACTTCTGCCCATGGTTCCAGCATTCGGGTCGGCAGTGACAACTGGGAAAGTTGTATGCCCACAACTTTTTCATCCTTGTCCCTACAG

>Exon 5

CCGGTGGCTCTCGGCTATGAACTATTTGAGGGACGGCCACGACTGGCAGCATGGCGTGGACGAGTGGAGGCTTTCCTGGGTGCTGAGCTATGCCAGGAGGCCCACAGCATCATCTTGAGCATCCTGGAACAGGCGGCCAAGAAAACCCTCCCAACACCCTCACCAGAGGCCTATCAGGCTATGCTGCTTCGAATCGCCAGGATCCCCTGA

>Post

AGGGTCTGGGATGGGGGCCAGGAGATTAGCAACAAGGATTCATTCTGTTACTTACTTGCCCCTTTTTATCTTTCCCTCTTGCCCCAGTCCCTTCTCTCCAGCTTCATGTGAAGCTCTGCACAGACAAGACACTCAGTGTCCTTGGCAGTGCTGCTACTCCTCAGGTGCAGCATACATAACCAGTAAGAGACTAAATCTGCAATATATAAAGAGCTCCTACAAATCAATAACATGAAGAACACTCAAAAATTGGCAAATGTCATCAGTGTTTTAAATAGAATAAAGATTCCAAACACTTTGAATAGAGAACCAAGAGTTATTGGTTTTACTACATTGTTGTGTTATACATATGGAGTAAAAGTATGTGCTAGTAATCCTCATCATGGTTAACCAAAGTAACCTCACAATAACAAGTCAACATAATTGTATCACCAGGGCAACAAAATATTAAGTAAGTAACCAATTCGAATTGCAAACTGTTAAAGGATATAGGCGATGTTTCACAGGGCATAGCAACGGTCTTTGAAGTCTAGGAA

**Accession No. JN819432 - Chimpanzee Fibroblast-2 (NO AVA)**

>Promoter CCGTGAGAGTAACTGGGATTGCAGGTGTGGGCCAGCATGCCCTGTTATCACTCCCTTTCTCTGAGTCAGCTTTTCTCTCCCAGACTCGTCTACTCCTTGAACTCAGGAAATGTCACTACAGGTGGCCCCAGCTCCCATTTACCTCCCCATAGGGAGCTCCTCTCTCCAGTTCCAATTTCAAAACTCTCAAGGAAGCATTCTGGTTCACTCGCTTGGGCCACTGGCCAGAGGGATGGGATACTCTGAAAGATTCAGCTAGAGTCCCGGGCCCAGCCCTGGACCATCACTGTGCCCCCTGGTGAGATGCCAGGGCTGGGATTCAGGGAGAAGAAAGGAGGTTCCCGGACAGTCATTCCTGCCTCCCGCGGCTGCGGGCTCCCTGCCCCCATCCTGTGCACGAAGTGGGAGCTCCCGCTGTCTGGCAGCAGCTGCTCTGCAGGGGACAGTCTGGACGGTAGAAAGTTCATCCTTAACCCCAGCCTTCCAGTCAAGGTTCCCACCAGTTTGGGACACCTGCAAGTGTCACATCCCACTGGGTGAAACTCTAAGATCCCTTTTAGAGGATCCCATTCGCTCCCTCCCTTCCGCCACCATGCAGCGCCGAGAAACAGAGCTCTGAACGAACCCTCAGATGCCCGTGCGCTGGGGCCTTTCCAGGACGGCGGCGCCCAGTCGTTTCTGGGTCGGGGCGACGCCTGGAACTGGGCAGGGTCCCTGGCACAGGGATCCCGAAAAGCAGACCTGCTTCTCCCTGTCCAGCCGGTTCCCCTTCCCCTTGCTGTCGGCCCCCTGCATCCGCGTCCTCCCTGCCAGTAGAGGGCCCCCAGCTCCAACTCCACCCTCCCAGCTGTGGGTTCATAGCGACCGCCCTCCCTGTAGGGACGCACGGATCTGGTGGTGGAGTCTTGGCCGGCAGGACTGGACAGGAACCGAAGGGGCGAGGCGGGTCCGGGGGTGGTGCGCTCCAATTGGGTGCTGTCCCCAGGGGGTGGGGCCTGATCCCCTATTTCCCGGCGCGCCGG

>Exon 1

GATCCTGCCACAGCTGCTGCCCACACCGCGCTCAGCGCCTTCACTGCCTTCCCCGCTGTCCTTGCCGCCCCCGCCATGGGCCTAGAGCTGTTTCTTGACCTGGTGTCCCAGCCCAGCCGCGCCGTCTACATCTTCGCCAAGAAGAATGGCATCCCCTTAGAGCTGCGCACCGTGGATCTTATCAAAG

>Intron 1

GTGGGCCCAGCCCGTTTCCCCGCGTGTCCACAAACCCAGTGCGCCCCCAGGCCCCCGCCCTGCTCTGCCCTGAGCGTCTCGCCGCCCGCACAGCCCCCTCACCTCCTCCTGCAGCGTCTGCCACCAGAGAATGCTGTGGACTGAGTGGCCTTGGAGGGATCACAGCCTCTCTGAACCTTAGCTTGCCTTCTGAAAAGGAGGATAAGGTTACCTTCTGCTCTGTAGGGATGGAAAGAAAATACTGAATGGAGTTTACAGAGTTCTTGCGTGGAATGCACGCATATAAATTCACAAAGCCCAGAAGACCTTGGGAAGAAGGACATGCTGTTGTGAGAATTAAGAGATGGGAAGAGATGAGCCACCCCAGTTTGCCTCCCCTCCCCTGGCCCACCAGAGTCCGGCTAGAAAACTTCTCTTTATCCACCTGCTGCACCTGGCCCCACCCACCAAAGCCCCCCAGCTGCCCCGGAATGTGGCAGGGCAGGGAGGCCCAGCCAGGGAGTGAGGCTGATCCAGGCCTCTAGTCCCAGACCTTGCTGTTTCTCAGGGCTGTGGGGTTCCGCTTGGGGAGGGGGAGGGAGGGTGTAGAGGTGCAGCGTTTTTACTCTGAAGACCTTTTGTGACTTCTTCCTCTTCAG

>Exon 2

GGCAGCACAAGAGCAAGGAGTTCTTGCAGATCAACAGCCTGGGGAAACTGCCGACGCTCAAGGATGGTGATTTCATCTTGACCGAAAG

>Intron 2

GTGCCCTCCTTCCCTCACCCCTCACCGCATCCGGAGCCCCTGTGACCCTGGCTCTCCCCACTGGCCCCCGGGCCCCACTGGCCCTCCCATACCCCATGGGGCAGCGAGGGAGGGGAAAGGCGAGGGATCTGGCCGGGCGCGGTGGCTCACGCCTGTCATCCCAGCACTTCGGGAGGCCAAGGCGGGCGGATCACGAGGTCAGGAGACCGAGACCATCCTGGCTAACACAGTGAAACACCGTCTCTACTAAAAATACAAAAAAAAAAAAAAAAAAAATTACCCGGGCGTGGTGGCGGGCGTCTGTAGTCCCAGCTACTCGGGAGGCTGAGGCAGGAGAATGGCGTGAACCCAGGAGGCGGAGCTTGCGGTGAGCTGAGATCGCGCCACTGCACTCCATCCTCGGCGACACAGCTAGACTCCGTCTCAAAAAGGTCGGGCGCGGTGTCTCACGCCTGTAATCCCAGCACTTTGGGAGGCCGAGGTGGGCGGATCACGAGGTCAGGAGATCGAGACCATCCTGGCTAACACGGTGAAACCCCGTCTGTACTAAAAATACAAAACATTAGCAGGGCGTGGTGGCGGGCGCCTGTAGTCCCAGCTACTCCAGAGGCTGAGGCAGGAGAATGGCGTGAACCGGGGAGGCGGAGCTTGCAGTGAGCTCGCGCCACTACACTCCAGCCTGGGCGAGAGAGGGAGACTCCGTCTCAAAAAAAAAAAAAAAGAAAGAAAGAAAAGAAAAAAAATAAAGGAGAGGGATCTGGGGAGAAGGTACAGCTTGGGGTGTGTCCGGGATGAGCAGGGGCTGACAGAGCATGTCCCCACACCTCTCATCTTCAGCCTTTTCTGAGCCGCAGGGCCTCTCCACTCCCAGACTGAAGGCTATTAGAAGAGAAGACAAGGGAACATTTTCCCACTGTTGCGCAGAGTAGGTTCAACAAATGCCAGCTGAAAAGAGCCTCTAGTGACTTGTCGCAGACTACCCCAATCTGCCTAGGCTGGGCCTAGAGGCCAATGCCATGGCCCAAGGGCACAGCTCATGGTGAGGTCCAGCTGCTGGGCAGGAAAAGGACAAGAGGTCAGGTGGCTGCAGAAGTGATGGCTGGGGGCCTGTCAGACGAGGGCCAAAGACATTCCTCCTCTCGTGATCCCTGACCCAAGTGCGTGGACATGCAAGGGACTCCACGGAGCATCCACTGTGTGCCAGCCCCATGCAGGGTTCCAGGGGTCCAGGGAGCCTATTCTGAGCTGCACCGCCTCAGACAAGTCACTTGACCGTTCTGACCTTGAGTTTTCTCTTGTGCTAAAAGGCTAACAGGAGTCTCTACCTCACAGGGCGGCTGCTGGCATATCACAGAGATGAGGGTCTCAAAGTGCAAAGCAGAAGGTCCAGCCAAGAGTCGGTGCCCAAGGCAACAAAGACAGGAGGAGACTCGTAGGAGGAGGGGGTGGTGTTGGGGAGCTGGAGATGGAGGGCGAGGCTGGAGGGCAGTCCTTCAAATGCAGAGAAGCCCCTGGACCCCACTGGCGGATGGGAGCAGCTAGGGGTAAATGCCTGGTGCCAGTGTCCTTATAGCCACTGCCCATTTGTTCCCAG

>Exon 3

CTCGGCCATCCTGATTTACCTGAGCTGTAAGTACCAGACGCCGGACCACTGGTATCCATCTGACCTGCAGGCTCGTGCCCGTGTTCATGAGTACCTGGGCTGGCATGCCGACTGCATCCGTGGCACCTTTGGTATACCTCTGTGGGTCCAG

>Intron 3

GTGAGGAGAGCCATCTGGAGAGTGATTGGCCATCAAAGAGTAGTTGGCAGTAGGCCGGGGCCATAGACTGACCCTCTCTCTGCCCCCATCAG

>Exon 4

GTGTTGGGGCCACTCATTGGGGTCCAGGTGCCCAAGGAGAAGGTGGAACGCAACAGGACTGCCATAGACCAGGCCCTGCAATGGCTGGAGGACAAGTTCCTGGGGGACATGCCCTTCCTTGCTGGCCAGCAGGTGACACTGGCTGATCTCATGGCTCTGGAGGAGCTGATGCAG

>Intron 4

GTGTGAGCTCAGCCTGTGGGCAGTGTCCCTCTTCGTGTCACACCCATGAGGCAGACAGACACACTGAGGCCTGGAGAAAGCCAGAACTTTGCCCAGAATCATAGAGCAAGTCTCTGGATGATCTGGGGCCAGAACCCTGAACTTCTGCCTCCTGCCTGGGTGTGGGGTCTCACCCTGGCTGCTCTTGGGCTCTAAGGCTGAACATACTGCCTGGGCCACTGTGGTCCATTCACTTAGGGGCTGGGGAATGGGCCAAGTCTCTGAAAGTTCTGCCCATGGTTCCAGCATTCGGTCGGCAGTGACAGCTGGGAAAGGTGGATGCCCACAACTTTTTGATCCATGTCCCTACAG

>Exon 5

CCGGTGGCTATCGGCTATGAGCTATTGAGGGACGGCCACGACTGGCAGCATGGCGTGGGCGAGTGGAGGCTTTCCTGGGTGCTGAGCTATGCCAGGAGGCCCACAGCATCATCTTGAGCATCATGGAACAGGGGGCCAAAAAAACCCTCCCAACACCCTCACCAGAGGCCTATCAAGCTATGCTACTTCGAATCGCCAGGATCCCCTGA

>Post

AGGGTCTGGGATGGGGGCCAGGAGATTAGCAACAAGGATTCATTCTGTTACTTACTTGCCCCATTTTATCTTTCCCTCGTGCCCCAGTCCCTTCTCTCCAGCTTCATGTGAAGCTCTGCACAGACAAGACACTCAGTGTCCTCGGCAGTGCTGCTACTCCTCAGGTGCAGCATACATAACCAGTAAGAGACTAAATCTGCAATATATAAAGAGCTCCTACAAATCAGTAACATGAAGAACACTCAAAAATTGGCAAATGTCATCAGTGTTTTAAATAGAATAAAGATTCCAAACACTTTGAATAGAGAACCAAGAGTTATTGGTTGTACTACATTGTTGTGTTATACATATGGAGTAAAAGTATGTGCTAGTAATCCTCATCATGGTTAATAACAAAGTAACCTCACAATAACAAGTGAACATAATTGTATCACCAGGGCAACAAAATGTTAAGTAAGTAACCAATTCAAATTGCAAACTATTAAAGGATATAGGCGATGTTTCACAGGGCATAGCAACGGTCTTTGAAGTCTAGGAA

**Accession No. JN819434 - Chimpanzee Fibroblast-3 (AVA)**

>Promoter

CCGTGAGAGTAACTGGGATTGCAGGTGTGGGCCAGCATGCCCTGTTATCACTCCCTTTCTCTGAGTCAGCTTTTCTCTCCCAGACTCGTCTACTCCTTGAACTCAGGAAATGTCACTACAGGTGGCCCCAGCTCCCATTTACCTCCCCATAGGGAGCTCCTCTCTCCAGTTCCAATTTCAAAACTCTCAAGGAAGCATTCTGGTTCACTCGCTTGGGCCACTGGCCAGAGGGATGGGATACTCTGAAAGATTCAGCTAGAGTCCCGGGCCCAGCCCTGGACCATCACTGTGCCCCCTGGTGAGATGCCAGGGCTGGGATTCAGGGAGAAGAAAGGAGGTTCCCGGACAGTCATTCCTGCCTCCCGCGGCTGCGGGCTCCCTGCCCCCATCCTGTGCACGAAGTGGGAGCTCCCGCTGTCTGGCAGCAGCTGCTCTGCAGGGGACAGTCTGGACGGTAGAAAGTTCATCCTTAACCCCAGCCTTCCAGTCAAGGTTCCCACCAGTTTGGGACACCTGCAAGTGTCACATCCCACTGGGTGAAACTCTAAGATCCCTTTTAGAGGATCCCATTCGCTCCCTCCCTTCCGCCACCATGCAGCGCCGAGAAACAGAGCTCTGAACGAACCCTCAGATGCCCGTGCGCTGGGGCCTTTCCAGGACGGCGGCGCCCAGTCGTTTCTGGGTCGGGGCGACGCCTGGAACTGGGCAGGGTCCCTGGCACCGGGATCCCGAAAAGCAGACCTGCTTCTCCCTGTCCAGCCGGTTCCCCTTCCCCTTGCTGTCGGCCCCCTGCATCCGCGTCCTCCCTGCCAGTAGAGGGCCCCCAGCTCCAACTCCACCCTCCCAGCTGTGCGTGGATAGCGACCGCCCTCCCTGTAGGGACGCACGGACCTGGTGGTGGAGTCTTGGCCGGCAGGACTGGACAGAAACGGAAGGGGCGAGGCGGGTCCGGGGGTGGTGCGCTCCAATTGGGTGCTGTCCCCAGGGGGTGGGGCCTGATCCCCTATTTCCCGGCGCGCCGG

>Exon 1

GATCCTGCCACAGCTGCTGCCCACACCGCGCTCAGCGCCTTCACTACCTTCCCCGCTGTCCTTGCCGCCCCCGCCATGGGCCTAGAGCTGTTTCTTGACCTGGTGTCCCAGCCCAGCCGCGCCGTCTACATCTTCGCCAAGAAGAATGGCATCCCCTTAGAGCTGCGCACCGTGGATCTTATCAAAG

>Intron 1

GTGGGCCCAGCCCGTTTCCCCGCGTGTCCACAAACCCAGTGGGCCCCCAGGCCCCCGCCCTGCTCTGCCCTGAGCGTCTCGCCGCCCGCACAGCCCCCTCACCTCCTCCTGCAGCGTCTGCCACCAGAGAATGCTGTGGACTGAGTGGCCTTGGAGGGATCACAGCCTCTCTGAACCTTAGCTTGCCTTCTGAAAAGGAGGATAAGGTTACCTTCTGCTCTGTAGGGATGGAAAGAAAATACTGAATGGAGTTTACAGAGTTCTTGCGTGGAATGCACGCATATAAATTCACAAAGCCCAGAAGACCTTGGGAAGAAGGACATGCTGTTGTGAGAATTAAGAGATGGGAAGAGATGAGCCACCCCAGTTTGCCTCCCCTCCCCTGGCCCACCAGAGTCCGGCTAGAAAACTTCTCTTTATCCACCTGCTGCACCTGGCCCCACCCACCAAAGCCCCCCAGCTGCCCCGGAATGTGGCAGGGCAGGGAGGCCCAGCCAGGGATTGAGGCTGATCCAGGCCTCTAGTCCCAGACCTTGCTGTTTCTCAGGGCTGTGGGGCTCCGCTTGAGGAGGGGGAGGGAGGGTGTAGAGGTGCAGCGTTTTTACTCTGAAGACCTTTTGTGACTTCTTCCTCTTCAG

>Exon 2

GGCAGCACAAGAGCAAGGAGTTCTTGCAGATCAACAGCCTGGGGAAACTGCCGACGCTCAAGGATGGTGATTTCATCTTGACCGAAAG

>Intron 2

GTGCCCTCCTTCCCTCACCCCTCACCGCATCCGGAGCCCCTGTGACCCTGGCTCTCCCCACTGGCCCCCGGGCCCCACTGGCCCTCCCATACCCCATGGGGCAGCGAGGGAGGGGAAAGGCGAGGGATCTGGCCGGGCGCGGTGGCTCACGCCTGTCATCCCAGCACTTCGGGAGGCCAAGGCGGGCGGATCACGAGGTCAGGAGACCGAGACCATCCTGGCTAACACCGTGAAACACCGTCTCTACTAAAAATGGAAAAAAAAATTAACCGGGCGTGGTGGCGGGCGTCTGTAGTCCCAGCTACTCGGGAGGCTGAGGCAGGAGAATGGCGTGAACCCAGGAGGCGGAGCTTGCGGTGAGCTGAGATCGCGCCACTGCACTCCATCCTGGGCGACAGAGCTAGACTCCGTCTCAAAAAGGTCGGGCGCGGTGTCTCACGCCTGTAATCCCAGCACTTTGGGAGGCCGAGGTGGGCGGATCACGAGGTCAGGAGATCGAGACCATCCTGGCTAACACGGTGAAACCCCGTCTGTACTAAAAATACAAAACATTAGCAGGGCGTGGTGGCGGGCGCCTGTAGTCCCAGCTACTCCAGAGGCTGAGGCAGGAGAATGGCGTGAACCGGGGAGGCGGAGCTTGCAGTGAGCTCGCGCCACTACACTCCAGCCTGGGCGAGAGAGGGAGACTCCGTCTCAAAAAAAAAAAAAAAAGAAAGAAAGAAAAGAAAAAAAATAAAGGAGAGGGATCTGGGGAGAAGGTACAGCTTGGGGTGTGTCCGGGATGAGCAGGGGCTGACAGAACATGTCCCCGCACCTCTCATCTTCAGCCTTTTCTGAGCCGCAGGGCCTCTCCACTCACAGACTGAAGGCTATTAGAAGAGAAGACAAGGGAACATTTTTCCACTGTTGCGCATTTGTTCAACAAATGCTAGCTGAAAAGAGCCTCTAGTGACTTGTCGCAGACTACCCAATCTACCCAGGCCAGGCCTAGAGGCCAATGCCATGGCCCAAGGGCACAGCTCATGGTGAGGTCCAGCTGCTGGGCAGGAAAAGGACAAGAGGTCAGGTGGCTGCAGAAGTGATGGCTGGGGGCCTGTCAGACGGGGGCCAAAGACATTCCTCCCCTCGTGATCCCTGACCCAAGCGCGTGGACATGCAAGGGACTCCACGGAGCATCCACTGTGTGCCAGCCCCATGCAGGGTTCCAGGGGTCCAGGGAGCCTATTCTGAGCTGCACCGCCTCGGACAAGTCACTTGACCATTTCTGACCTTGAGTTTTCTCTTGTGCTAAAAGGCTAACAGGAGTCTCTACCTCACAGGGCGGCTGCTGGCATATCACAGAGATGAGGGTCTCAAAGTGCAAAGCAGAAGGTCCAGCCAAGAGTCGGTGCCCAAGGCAACAAAGACAGGAGGAGACTCGTAGGAGGAGGGGGTGGTGTTGGGGAGCTGGAGCTGGAGGGCGAGGCTGGAGGGCAGTCCTTCAAATGCAGAGAAGCCCCCGGGCCCCACTGGCAGATGGGAGCAGTTAGGGGTAAATGCCTGGTGCCAGTGTCCTTATAGCGACTGCCCATTTGTTCCCAG

>Exon 3

CTCGGCCATCCTGATTTACCTGAGCTGTAAGTACCAGACGCCGGACCACTGGTATCCATCTGACCTGCAGGCTCGTGCCCGTGTTCATGAGTACCTGGGCTGGCATGCCGACTGCATCCGTGGCACCTTTGGTATACCTCTGTGGGTCCAG

>Intron 3

GTGAGGAGCGCCATCTGGAGAGTGATTGGCCATCAGAAAGTAGTTGGCAGTAGGCCGGGGCCATAGACTGACCCACTCTCTGCCCCCATCAG

>Exon 4

GTGTTGGGGCCACTCATTGGGGTCCAGGTGCCCGAGGAGAAGGTGGAACGCAACAGGACTGCCATAGACCAGGCCCTGCAATGGCTGGAGGACAAGTTCCTGGGGGACAGGCCCTTCCTTGCTGGCCAGCAGGTGACACTGGCTGATCTCATGGCTCTGGAGGAGCTGATGCAG

>Intron 4

GTGTGAGCTCAGCCTGTGGGCAATGTCCCTCTTCGTGTCACACCCATGAGGCAGACAGACACACTGAGGCCTGGAGAAAGCCAGAACTTTGCCCAGAATCATAGAGCAAGTCTCTGGATGATCTGGGGCCAGAACCCTGAACTTCTGCCTCCTGCCTGGGTGTGGGGTCTCACCCTGGCTGCTCTTGGGCTCTAAGGCTGAACATACTGCCTGGGCCGCTGTGGTCCATTCACTTAGGGGCTGGGGAATGGACCATGTCTCTGATACTTCTGCCCATGGTTCCAGCATTCGGGTCGGCAGTGACAACTGGGAAAGTTGTATGCCCACAACTTTTTCATCCTTGTCCCTACAG

>Exon 5

CCGGTGGCTCTCGGCTATGAACTATTTGAGGGACGGCCACGACTGGCAGCATGGCGTGGACGAGTGGAGGCTTTCCTGGGTGCTGAGCTATGCCAGGAGGCCCACAGCATCATCTTGAGCATCCTGGAACAGGCGGCCAAGAAAACCCTCCCAACACCCTCACCAGAGGCCTATCAGGCTATGCTGCTTCGAATCGCCAGGATCCCCTGA

>Post

AGGGTCTGGGATGGGGGCCAGGAGATTAGCAACAAGGATTCATTCTGTTACTTACTTGCCCCTTTTTATCTTTCCCTCTTGCCCCAGTCCCTTCTCTCCAGCTTCATGTGAAGCTCTGCACAGACAAGACACTCAGTGTCCTTGGCAGTGCTGCTACTCCTCAGGTGCAGCATACATAACCAGTAAGAGACTAAATCTGCAATATATAAAGAGCTCCTACAAATCAATAACATGAAGAACACTCAAAAATTGGCAAATGTCATCAGTGTTTTAAATAGAATAAAGATTCCAAACACTTTGAATAGAGAACCAAGAGTTATTGGTTTTACTACATTGTTGTGTTATACATATGGAGTAAAAGTATGTGCTAGTAATCCTCATCATGGTTAACCAAAGTAACCTCACAATAACAAGTCAACATAATTGTATCACCAGGGCAACAAAATATTAAGTAAGTAACCAATTCGAATTGCAAACTGTTAAAGGATATAGGCGATGTTTCACAGGGCATAGCAACGGTCTTTGAAGTCTAGGAA

**Accession No. JN819435 - Chimpanzee Fibroblast-3 (NO AVA)**

>Promoter

CCGTGAGAGTAACTGGGATTGCAGGTGTGGGCCAGCATGCCCTGTTATCACTCCCTTTCTCTGAGTCAGCTTTTCTCTCCCAGACTCGTCTACTCCTTGAACTCAGGAAATGTCACTACAGGTGGCCCCAGCTCCCATTTACCTCCCCATAGGGAGCTCCTCTCTCCAGTTCCAATTTCAAAACTCTCAAGGAAGCATTCTGGTTCACTCGCTTGGGCCACTGGCCAGAGGGATGGGATACTCTGAAAGATTCAGCTAGAGTCCCGGGCCCAGCCCTGGACCATCACTGTGCCCCCTGGTGAGATGCCAGGGCTGGGATTCAGGGAGAAGAAAGGAGGTTCCCGGACAGTCATTCCTGCCTCCCGCGGCTGCGGGCTCCCTGCCCCCATCCTGTGCACGAAGTGGGAGCTCCCGCTGTCTGGCAGCAGCTGCTCTGCAGGGGACAGTCTGGACGGTAGAAAGTTCATCCTTAACCCCAGCCTTCCAGTCAAGGTTCCCACCAGTTTGGGACACCTGCAAGTGTCACATCCCACTGGGTGAAACTCTAAGATCCCTTTTAGAGGATCCCATTCGCTCCCTCCCTTCCGCCACCATGCAGCGCCGAGAAACAGAGCTCTGAACGAACCCTCAGATGCCCGTGCGCTGGGGCCTTTCCAGGACGGCGGCGCCCAGTCGTTTCTGGGTCGGGGCGACGCCTGGAACTGGGCAGGGTCCCTGGCACCGGGATCCCGAAAAGCAGACCTGCTTCTCCCTGTCCAGCCGGTTCCCCTTCCCCTTGCTGTCGGCCCCCTGCATCCGCGTCCTCCCTGCCAGTAGAGGGCCCCCAGCTCCAACTCCACCCTCCCAGCTGTGGGTTCATAGCGACCGCCCTCCCTGTAGGGACGCACGGACCTGGTGGTGGAGTCTTGGCCGGCAGGACTGGACAGGAACCGAAGGGGCGAGGCGGGTCCGGGGGTGGTGCGCTCCAATTGGGTGCTGTCCCCAGGGGGTGGGGCCTGATCCCCTATTTCCCGGCGCGCCGG

>Exon 1

GATCCTGCCACAGCTGCTGCCCACACCGCGCTCAGCGCCTTCACTGCCATCCCCGCTGTCCTTGCCGCCCCCGCCATGGGCCTAGAGCTGTTTCTTGACCTGGTGTCCCAGCCCAGCCGCGCCGTCTACATCTTCGCCAAGAATGGCATCCCCTTAGAGCTGCGCACCGTGGATCTTATCAAAG

>Intron 1

GTGGGCCCAGCCCGTTTCCCCGCGTGTCCACAAACCCAGTGCGCCCCCAGGCCCCCGCCCTGCTCTGCCCTGAGCGTCTCGCCGCCCGCACAGCCCCCTCACCTCCTCCTGCAGCGTCTGCCACCAGAGAATGCTGTGGACTGAGTGGCCTTGGAGGGATCACAGCCTCTCTGAACCTTAGCTTGCCTTCTGAAAAGGAGGATAAGGTTACCTTCTGCTCTGTAGGGATGGAAAGAAAATACTGAATGGAGTTTACAGAGTTCTTGCGTGGAATGCACGCATATAAATTCACAAAGCCCAGAAGACCTTGGGAAGAAGGACATGCTGTTGTGAGAATTAAGAGATGGGAAGAGATGAGCCACCCCAGTTTGCCTCCCCTCCCCTGGCCCACCAGAGTCCGGCTAGAAAACTTCTCTTTATCCACCTGCTGCACCTGGCCCCACCCACCAAAGCCCCCCAGCTGCCCCGGAATGTGGCAGGGCAGGGAGGCCCAGCCAGGGATTGAGGCTGATCCAGGCCTCTAGTCCCAGACCTTGCTGTTTCTCAGGGCTGTGGGGCTCCGCTTGGGGAGGGGGAGGGAGGGTGTAGAGGTGCAGCGTTTTTACTCTGAAAACCTTTTGTGACTTCTTCCTCTTCAG

>Exon 2

GGCAGCACAAGAGCAAGGAGTTCTTGCAGATCAACAGCCTGGGGAAACTGCCGACGCTCAAGGATGGTGATTTCATCTTGACCGAAAG

>Intron 2

GTGCCCTCCTTCCCTCACCCCTCACCGCATCCGGAGCCCATGTGACCTTGGCTCTCCCCACTGGCCCCGGGCCCCAATGGCCCTCCCATACCCCATGGGGCAGCGAGGGAGGGGAAAGGCGAGGGATCTGGCCGGGCGCGGTGGCTCACGCCTGTCATCCCAGCACTTCGGGAGGCCAAGGCGGGCGGATCACGAGGTCAGGAGACCGAGACCATCCTGGCTAACATAGTGAAACACCGTCTCTACTAAAAATACAAAAAAAAAAAAAAAAAAAAAAAAAAAAAAATTAGCCGGGCGTGGTGGCGGGCGTCTGTAGTCCCAGCTACTCGGGAGGCTGAGGCTGGAGAATGGCGTGAACCCAGGAGGCGGAGCTTGCGGTGAGCTGAGATCGCGCCACTGCACTCCATCCTGGGCGACAGAGCTAGACTCCGTCTCAAAAAGGTCGGGCGCGGTGGCTCACGCCTGTAATCCCAGCACTTTGGGAGGCCGAGGTGGGCGGATCACGAGGTCAGGAGATCGAGACCATCCTGGCTAACACGGTGAAACCCCGTCTGTACTAAAAATACAAAACATTAGCAGGGCGTGGTGGCGGGCGCCTGTAGTCCCAGCTACTTCAGAGGCTGAGGCAGGAGAATGGCGTGAACCCGGGGAGGCGGAGCTTGCAGTGAGCTCGCGCCACTACACTCCAGTCTGGGCGACAGAGGGAGACTCCGTCTCAAAAAAAAAAAAAAGAAAGAAAGAAAAGAAAAAAAAAAAAAGGAGAGGGATCTGGGGAGAAGGTACAGCTTGGGGTGTGTCCGGGATGAGCAGGGGCTGACAGAGCATGTCCCCGCACCTCTCATCTTCAGCCTTTTCTGAGCCGCAGGGCCTCTCCACTCCCAGACTGAAGGCTATTAGAAGAGAAGACAAGGGAACATTTTCCCACTGTTGCGCAGAGTAGGTTCAACAAATGCCAGCTGAAAAGAGCCTCTAGTGACTTGTCGCAGACTACCCCAATCTGCCTAGGCTGGGCCTAGAGGCCAATGCCATGGCCCAAGGGCACAGCTCATGGTGAGGTCCAGCTGCTGGGCAGGAAAAGGACAAGAGGTCAGGTGGCTGCAGAAGTGATGGCTGGGGGCCTGTCAGACGAGGGCCAAAGACATTCCTCCTCTCGTGATCCCTGACCCAAGTGCGTGGACATGCAAGGGACTCCACGGAGCATCCACTGTGTGCCAGCCCCAGGCAGGGTTCCAGGGGTCCAGGGAGCCTATTCTGAGCTGCACCGCCTCAGACAAGTCACTTGACCGTTCTGACCTTGAGTTTTCTCTTGTGCTAAAAGGCTAACAGGAGTCTCTACCTCACAGGGCGGCTGCTGGCATATCACAGAGATGAGGGTCTCAAAGTGCAAAGCAGAAGGTCCAGCCAAGAGTCGGTGCCCAAGGCAACAAAGACAGGAGGAGACTCGTAGGAGGAGGGGGTGGTGTTGGGGAGCTGGAGATGGAGGGCGAGGCTGGAGGGCAGTCCTTCAAATGCAGAGAAGCCCCTGGGCCCCACTGGCGGATGGGAGCAGCTAGGGGTAAATGCCTGGTACCAGTGTCCTTATGGCCACTGCCCATTTGTTCCCAG

>Exon 3

CTCGGCCATCCTGATTTACCTGAGCTGTAAGTACCAGACGCCGGACCACTGGTATCCATCTGACCTGCAGGCTCGTGCCCGTGTTCATGAGTACCTGGGCTGGCATGCCGACTGCATCCGTGGCACCTTTGGTATACCCCTGTGGGTCCAG

>Intron 3

GTGAGGAGAGCCATCTGGAGAGTGATTGGCCATCAGAGAGTAGTTGGCAGTAGGCCGGGGCCATAGACTGACCCTCTCTCTGCCCCCATCAG

>Exon 4

GTGTTGGGGCCACTCATTGGGGTCCAGGTGCCCAAGGAGAAGGTGGAACGCAACAGGACTGCCATAGACCAGGCCCTGCAATGGCTGGAGGACAAGTTCCTGGGGGACAGGCCCTTCCTTGCTGGCCAGCAGGTGACACTGGCTGATCTCATGGCTCTGGAGGAGCTGATGCAG

> Intron 4

GTGTGAGCTCAGCCTGTGGGCAGTGTCCCTCTTCGTGTCACACCCATGAGGCAGACAGACACACTGAGGCCTGGAGAAAGCCAGAACTTTGCCCAGAATCATAGAGCAAGTCTCTGGATGATCTGGGGCCAGAACCCTGAACTTCTGCCTCCTGCCTGGGTGTGGGGTCTCACCCTGGCTGCTCTTGGGCTCTAAGGCTGAACATACTGCCTGGGCCACTGTGGTCCATTCACTTAGGGGCTGGGGAATGGACCATGTCTCTGATACTTCTGCCCATGGTTCCAGCATTCGGGTCGGCAGTGACAACTGGGAAAGTTGTATGCCCACAACTTTTTCATCCTTGTCCCTACAG

>Exon 5

CCGGTGGCTCTCGGCTATGAACTATTTGAGGGACGGCCACGACTGGCAGCATGGCGTGGACGAGTGGAGGCTTTCCTGGGTGCTGAGCTATGCCAGGAGGCCCACAGCATCATCTTGAGCATCCTGGAACAGGCGGCCAAGAAAACCCTCCCAACACCCTCACCAGAGGCCTATCAGGCTATGCTGCTTCGAATCGCCAGGATCCCCTGA

>Post

AGGGTCTGGGATGGGGGCCAGGAGATTAGCAACAAGGATTCATTCTGTTACTTACTTGCCCCATTTTATCTTTCCCTCGTGCCCCAGTCCCTTCTCTCCAGCTTCATGTGAAGCTCTGCACAGACAAGACACTCAGTGTCCTTGGCAGTGCTGCTACTCCTCAGGTGCAGCATACATAACCAGTAAGAGACTAAATCTGCAATATATAAAGAGCTCCTACAAATCAGTAACATGAAGAACACTCAAAAATTGGCAAATGTCATCAGTGTTTTAAATAGAATAAAGATTCCAAACACTTTGAATAGAGAACCAAGAGTTATTGGTTTTACTACATTGTTGTGTTATACATATGGAGTAAAAGTATGTGCTAGTAATCCTCATCATGGTTAATAACAAAGTAACCTCACAATAACAAGTCAACATAATTGTATCACCAGGGCAACAAAATGTTAAGTAAGTAACCAATTCAAATTGCAAACTATTAAAGGATATAGGCGATGTTTCACAGGGCATAGCAACGGTCTTTGAAGTCTAGGAA

**Accession No. JN819437 - Chimpanzee Fibroblast-4 (AVA)**

>Promoter

CCGTGAGAGTAACTGGGATTGCAGGTGTGGGCCAGCATGCCCTGTTATCACTCCCTTTCTCTGAGTCAGCTTTTCTCTCCCAGACTCGTCTACTCCTTGAACTCAGGAAATGTCACTACAGGTGGCCCCAGCTCCCATTTACCTCCCCATAGGGAGCTCCTCTCTCCAGTTCCAATTTCAAAACTCTCAAGGAAGCATTCTGGTTCACTCGCTTGGGCCACTGGCCAGAGGGATGGGATACTCTGAAAGATTCAGCTAGAGTCCCGGGCCCAGCCCTGGACCATCACTGTGCCCCCTGGTGAGATGCCAGGGCTGGGATTCAGGGAGAAGAAAGGAGGTTCCCGGACAGTCATTCCTGCCTCCCGCGGCTGCGGGCTCCCTGCCCCCATCCTGTGCACGAAGTGGGAGCTCCCGCTGTCTGGCAGCAGCTGCTCTGCAGGGGACAGTCTGGACGGTAGAAAGTTCATCCTTAACCCCAGCCTTCCAGTCAAGGTTCCCACCAGTTTGGGACACCTGCAAGTGTCACATCCCACTGGGTGAAACTCTAAGATCCCTTTTAGAGGATCCCATTCGCTCCCTCCCTTCCGCCACCATGCAGCGCCGAGAAACAGAGCTCTGAACGAACCCTCAGATGCCCGTGCGCGGGGCCTTTCCAGGACGGCGGCGCCCAGTCGTTTCTGGGTCGGGGCGACGCCTGGAACTGGGCAGGGTCCCTGGCACCGGGATCCCGAAAAGCAGACCTGCTTCTCCCTGTCCAGCCGGTTCCCCTTCCCCTTGCTGTCGGCCCCCTGCATCCGCGTCCTCCCTGCCAGTAGAGGGCCCCCAGCTCCAACTCCACCCTCCCAGCTGTGCGTGGATAGCGACCGCCCTCCCTGTAGGGACGCACGGACCTGGTGGTGGAGTCTTGGCCGGCAGGACTGGACAGAAACGGAAGGGGCGAGGCGGGTCCGGGGGTGGTGCGCTCCAATTGGGTGCTGTCCCCAGGGGGTGGGGCCTGATCCCCTATTTCCCGGCGCGCCGG

>Exon 1

GATCCTGCCACAGCTGCTGCCCACACCGCGCTCAGCGCCTTCACTACCTTCCCCGCTGTCCTTGCCGCCCCCGCCATGGGCCTAGAGCTGTTTCTTGACCTGGTGTCCCAGCCCAGCCGCGCCGTCTACATCTTCGCCAAGAAGAATGGCATCCCCTTAGAGCTGCGCACCGTGGATCTTATCAAAG

>Intron 1

GTGGGCCCAGCCCGTTTCCCCGCGTGTCCACAAACCCAGTGGGCCCCCAGGCCCCCGTCCTGCTCTGCCCTGAGCGTCTCGCCGCCCGCACAGCCCCCTCACCTCCTCCTGCAGCGTCTGCCACCAGAGAATGCTGTGGACTGAGTGGCCTTGGAGGGATCACAGCCTCTCTGAACCTTAGCTTGCCTTCTGAAAAGGAGGATAAGGTTACCTTCTGCTCTGTAGGGATGGAAAGAAAATACTGAATGGAGTTTACAGAGTTCTTGCGTGGAATGCACGCATATAAATTCACAAAGCCCAGAAGACCTTGGGAAGAAGGACATGCTGTTGTGAGAATTAAGAGATGGGAAGAGATGAGCCACCCCAGTTTGCCTCCCCTCCCCTGGCCCACCAGAGTCCGGCTAGAAAACTTCTCTTTATCCACCTGCTGCACCTGGCCCCACCCACCAAAGCCCCCCAGCTGCCCCGGAATGTGGCAGGGCAGGGAGGCCCAGCCAGGGATTGAGGCTGATCCAGGCCTCTAGTCCCAGACCTTGCTGTTTCTCAGGGCTGTGGGGCTCCGCTTGAGGAGGGGGAGGGAGGGTGTAGAGGTGCAGCGTTTTTACTCTGAAGACCTTTTGTGACTTCTTCCTCTTCAG

>Exon 2

GGCAGCACAAGAGCAAGGAGTTCTTGCAGATCAACAGCCTGGGGAAACTGCCGACGCTCAAGGATGGTGATTTCATCTTGACCGAAAG

>Intron 2

GTGCCCTCCTTCCCTCACCCCTCACCGCATCCGGAGCCCCTGTGACCCTGGCTCTCCCCACTGGCCCCCGGGCCCCACTGGCCCTCCCATACCCCATGGGGCAGCGAGGGAGGGGAAAGGCGAGGGATCTGGCCGGGCGCGGTGGCTCACGCCTGTCATCCCAGCACTTCGGGAGGCCAAGGCGGGCGGATCACGAGGTCAGGAGACCGAGACCATCCTGGCTAACACCGTGAAACACCGTCTCTACTAAAAATGGAAAAAAAAATTAACCGGGCGTGGTGGCGGGCGTCTGTAGTCCCAGCTACTCGGGAGGCTGAGGCAGGAGAATGGTGTGAACCCAGGAGGCGGAGCTTGCAGTGTGCTGAGATCGCGCCACCGCACTCCATCCTGGGCGACAGAGCTAGACTCCGTCTCAAAAAGGCCGGGCGCGGTGGTTTACGCCTGTAATCCCAGCACTTTGGGAGGCCGAGGTGGGCGGATCACGAGGTCAGGAGATCGAGACCATCCTGGCTAACACGATGAAACCCTGTCTCTACTAAAAATACAAAACATTAGCAGGGCGTGGTGGCGGGTGCCTGTAGTCCCAGCTACTGGGGAGGCTGAGGCAGGAGAATGGCGTGAACCCAGGAGGCGGAGCTTGCAATGAGCAGAGATCGCGCCACTGCACTCCAGCCTGGGTGACAGAGGGAGCCCACTCCAGCCTGGGCGACAGAGGGAGACTCCGTCTCAAAAAAAAAGGAAAGAAAGAAAGGAGAGGTATCTGGGGAGAAGGTACAGCTTGGGGTGTGTCCGGGATGAGCAGGGGCTGACAGAACATGTCCCCGCACCTCTCATCTTCAGCCTTTTCTGAGCCGCAGGGCCTCTCCACTCACAGACTGAAGGCTATTAGAAGAGAAGACAAGGGAACATTTTTCCACTGTTGCGCATTTGTTCAACAAATGCTAGCTGAAAAGAGCCTCTAGTGACTTGTCGCAGACTACCCAATCTACCCAGGCCAGGCCTAGAGGCCAATGCCATGGCCCAAGGGCACAGCTCATGGTGAGGTCCAGCTGCTGGGCAGGAAAAGGACAAGAGGTCAGGTGGCTGCAGAAGTGATGGCTGGGGGCCTGTCAGACGGGGGCCAAAGACATTCCTCCCCTCGTGATCCCTGACCCAAGCGCGTGGACATGCAAGGGACTCCACGGAGCATCCACTGTGTGCCAGCCCCATGCAGGGTTCCAGGGGTCCAGGGAGCCTATTCTGAGCTGCACCGCCTCGGACAAGTCACTTGACCATTTCTGACCTTGAGTTTTCTCTTGTGCTAAAAGGCTAACAGGAGTCTCTACCTCACAGGGCGGCTGCTGGCATATCACAGAGATGAGGGTCTCAAAGTGCAAAGCAGAAGGTCCAGCCAAGAGTCGGTGCCCAAGGCAACAAAGACAGGAGGAGACTCGTAGGAGGAGGGGGTGGTGTTGGGGAGCTGGAGCTGGAGGGCGAGGCTGGAGGGCAGTCCTTCAAATGCAGAGAAGCCCCCGGGCCCCACTGGCAGATGGGAGCAGTTAGGGGTAAATGCCTGGTGCCAGTGTCCTTATAGCGACTGCCCATTTGTTCCCAG

>Exon 3

CTCGGCCATCCTGATTTACCTGAGCTGTAAGTACCAGACGCCGGACCACTGGTATCCATCTGACCTGCAGGCTCGTGCCCGTGTTCATGAGTACCTGGGCTGGCATGCCGACTGCATCCGTGGCACCTTTGGTATACCTCTGTGGGTCCAG

>Intron 3

GTGAGGAGAGCCATCTGGAGAGTGATTGGCCATCAGAAAGTAGTTGGCAGTAGGCCGGGGCCATAGACTGACCCACTCTCTGCCCCCATCAG

>Exon 4

GTGTTGGGGCCACTCATTGGGGTCCAGGTGCCCGAGGAGAAGGTGGAACGCAACAGGACTGCCATAGACCAGGCCCTGCAATGGCTGGAGGACAAGTTCCTGGGGGACAGGCCCTTCCTTGCTGGCCAGCAGGTGACACTGGCTGATCTCATGGCTCTGGAGGAGCTGATGCAG

>Intron 4

GTGTGAGCTCAGCCTGTGGGCAATGTCCCTCTTCGTGTCACACCCATGAGGCAGACAGACACACTGAGGCCTGGAGAAAGCCAGAACTTTGCCCAGAATCATAGAGCAAGTCTCTGGATGATCTGGGGCCAGAACCCTGAACTTCTGCCTCCTGCCTGGGTGTGGGGTCTCACCCTGGCTGCTCTTGGGCTCTAAGGCTGAACATACTGCCTGGGCCGCTGTGGTCCATTCACTTAGGGGCTGGGGAATGGACCATGTCTCTGATACTTCTGCCCATGGTTCCAGCATTCGGGTCGGCAGTGACAACTGGGAAAGTTGTATGCCCACAACTTTTTCATCCTTGTCCCTACAG

>Exon 5

CCGGTGGCTCTCGGCTATGAACTATTTGAGGGACGGCCACGACTGGCAGCATGGCGTGGACGAGTGGAGGCTTTCCTGGGTGCTGAGCTATGCCAGGAGGCCCACAGCATCATCTTGAGCATCCTGGAACAGGCGGCCAAGAAAACCCTCCCAACACCCTCACCAGAGGCCTATCAGGCTATGCTGCTTCGAATCGCCAGGATCCCCTGA

>Post

AGGGTCTGGGATGGGGGCCAGGAGATTAGCAACAAGGATTCATTCTGTTACTTACTTGCCCCTTTTTATCTTTCCCTCTTGCCCCAGTCCCTTCTCTCCAGCTTCATGTGAAGCTCTGCACAGACAAGACACTCAGTGTCCTTGGCAGTGCTGCTACTCCTCAGGTGCAGCATACATAACCAGTAAGAGACTAAATCTGCAATATATAAAGAGCTCCTACAAATCAATAACATGAAGAACACTCAAAAATTGGCAAATGTCATCAGTGTTTTAAATAGAATAAAGATTCCAAACACTTTGAATAGAGAACCAAGAGTTATTGGTTTTACTACATTGTTGTGTTATACATATGGAGTAAAAGTATGTGCTAGTAATCCTCATCATGGTTAACCAAAGTAACCTCACAATAACAAGTCAACATAATTGTATCACCAGGGCAACAAAATATTAAGTAAGTAACCAATTCGAATTGCAAACTGTTAAAGGATATAGGCGATGTTTCACAGGGCATAGCAACGGTCTTTGAAGTCTAGGAA

**Accession No. JN819436 - Chimpanzee Fibroblast- 4 (NO AVA)**

>Promoter

CCTGTTATCACTCCCTTTCTCTGAGTCAGCTTTTCTCTCCCAGACTCGTCTACTCCTTGAACTCAGGAAATGTCACTACAGGTGGCCCCAGCTCCCATTTACCTCCCCATAGGGAGCTCCTCTCTCCAGTTCCAATTTCAAAACTCTCAAGGAAGCATTCTGGTTCACTCGCTTGGGCCACTGGCCAGAGGGATGGGATACTCTGAAAGATTCAGCTAGAGTCCCGGGCCCAGCCCTGGACCATCACTGTGCCCCCTGGTGAGATGCCAGGGCTGGGATTCAGGGAGAAGAAAGGAGGTTCCCGGACAGTCATTCCTGCCTCCCGCGGCTGCGGGCTCCCTGCCCCCATCCTGTGCACGAAGTGGGAGCTCCCGCTGTCTGGCAGCAGCTGCTCTGCAGGGGACAGTCTGGACGGTAGAAAGTTCATCCTTAACCCCAGCCTTCCAGTCAAGGTTCCCACCAGTTTGGGACACCTGCAAGTGTCACATCCCACTGGGTGAAACTCTAAGATCCCTTTTAGAGGATCCCATTCGCTCCCTCCCTTCCGCCACCATGCAGCGCCGAGAAACAGAGCTCTGAACGAACCCTCAGATGCCCGTGCGCTGGGGCCTTTCCAGGACGGCGGCGCCCAGTCGTTTCTGGGTCGGGGCGACGCCTGGAACTGGGCAGGGTCCCTGGCACAGGGATCCCGAAAAGCAGACCTGCTTCTCCCTGTCCAGCCGGTTCCCCTTCCCCTTGCTGTCGGCCCCCTGCATCCGCGTCCTCCCTGCCAGTAGAGGGCCCCCAGCTCCAACTCCACCCTCCCAGCTGTGGGTTCATAGCGACCGCCCTCCCTGTAGGGACGCACGGATCTGGTGGTGGAGTCTTGGCCGGCAGGACTGGACAGGAACCGAAGGGGCGAGGCGGGTCCGGGGGTGGTGCGCTCCAATTGGGTGCTGTCCCCAGGGGGTGGGGCCTGATCCCCTATTTCCCGGCGCGCCGG

>Exon 1

GATCCTGCCACAGCTGCTGCCCACACCGCGCTCAGCGCCTTCACTGCCTTCCCCGCTGTCCTTGCCGCCCCCGCCATGGGCCTAGAGCTGTTTCTTGACCTGGTGTCCCAGCCCAGCCGCGCCGTCTACATCTTCGCCAAGAAGAATGGCATCCCCTTAGAGCTGCGCACCGTGGATCTTATCAAAG

>Intron 1

GTGGGCCCAGCCCGTTTCCCCGCGTGTCCACAAACCCAGTGCGCCCCCAGGCCCCCGCCCTGCTCTGCCCTGAGCGTCTCGCCGCCCGCACAGCCCCCTCACCTCCTCCTGCAGCGTCTGCCACCAGAGAATGCTGTGGACTGAGTGGCCTTGGAGGGATCACAGCCTCTCTGAACCTTAGCTTGCCTTCTGAAAAGGAGGATAAGGTTACCTTCTGCTCTGTAGGGATGGAAAGAAAATACTGAATGGAGTTTACAGAGTTCTTGCGTGGAATGCACGCATATAAATTCACAAAGCCCAGAAGACCTTGGGAAGAAGGACATGCTGTTGTGAGAATTAAGAGATGGGAAGAGATGAGCCACCCCAGTTTGCCTCCCCTCCCCTGGCCCACCAGAGTCCGGCTAGAAAACTTCTCTTTATCCACCTGCTGCACCTGGCCCCACCCACCAAAGCCCCCCAGCTGCCCCGGAATGTGGCAGGGCAGGGAGGCCCAGCCAGGGAGTGAGGCTGATCCAGGCCTCTAGTCCCAGACCTTGCTGTTTCTCAGGGCTGTGGGGTTCCGCTTGGGGAGGGGGAGGGAGGGTGTAGAGGTGCAGCGTTTTTACTCTGAAGACCTTTTGTGACTTCTTCCTCTTCAG

>Exon 2

GGCAGCACAAGAGCAAGGAGTTCTTGCAGATCAACAGCCTGGGGAAACTGCCGACGCTCAAGGATGGTGATTTCATCTTGACCGAAAG

>Intron 2

GTGCCCTCCTTCCCTCACCCCTCACCGCATCCGGAGCCCCTGTGACCCTGGCTCTCCCCACTGGCCCCCGGGCCCCACTGGCCCTCCCATACCCCATGGGGCAGCGAGGGAGGGGAAAGGCGAGGGATCTGGCCGGGCGCGGTGGCTCACGCCTGTCATCCCAGCACTTCGGGAGGCCAAGGCGGGCGGATCACGAGGTCAGGAGACCGAGACCATCCTGGCTAACACAGTGAAACACCGTCTCTACTAAAAATACAAAAAAAAAAAAAAAAAAAATTAGCCGGGCGTGGTGGCGGGCGTCTGTAGTCCCAGCTACTCGGGAGGCTGAGGCAGGAGAATGGCGTGAACCCAGGAGGCGGAGCTTGCGGTGAGCTGAGATCGCGCCACTGCACTCCATCCTGGGCGACAGAGCTAGACTCCGTCTCAAAAAGGTCGGGCGCGGTGTCTCACGCCTGTAATCCCAGCACTTTGGGAGGCCGAGGTGGGCGGATCACGAGGTCAGGAGATCGAGACCATCCTGGCTAACACGGTGAAACCCCGTCTGTACTAAAAATACAAAACATTAGCAGGGCGTGGTGGCGGGCGCCTGTAGTCCCAGCTACTCCAGAGGCTGAGGCAGGAGAATGGCGTGAACCGGGGAGGCGGAGCTTGCAGTGAGCTCGCGCCACTACACTCCAGCCTGGGCGAGAGAGGGAGACTCCGTCTCAAAAAAAAAAAAAAAGAAAGAAAGAAAAGAAAAAAAATAAAGGAGAGGGATCTGGGGAGAAGGTACAGCTTGGGGTGTGTCCGGGATGAGCAGGGGCTGACAGAGCATGTCCCCACACCTCTCATCTTCAGCCTTTTCTGAGCCGCAGGGCCTCTCCACTCCCAGACTGAAGGCTATTAGAAGAGAAGACAAGGGAACATTTTCCCACTGTTGCGCAGAGTAGGTTCAACAAATGCCAGCTGAAAAGAGCCTCTAGTGACTTGTCGCAGACTACCCCAATCTGCCTAGGCTGGGCCTAGAGGCCAATGCCATGGCCCAAGGGCACAGCTCATGGTGAGGTCCAGCTGCTGGGCAGGAAAAGGACAAGAGGTCAGGTGGCTGCAGAAGTGATGGCTGGGGGCCTGTCAGACGAGGGCCAAAGACATTCCTCCTCTCGTGATCCCTGACCCAAGTGCGTGGACATGCAAGGGACTCCACGGAGCATCCACTGTGTGCCAGCCCCATGCAGGGTTCCAGGGGTCCAGGGAGCCTATTCTGAGCTGCACCGCCTCAGACAAGTCACTTGACCGTTCTGACCTTGAGTTTTCTCTTGTGCTAAAAGGCTAACAGGAGTCTCTACCTCACAGGGCGGCTGCTGGCATATCACAGAGATGAGGGTCTCAAAGTGCAAAGCAGAAGGTCCAGCCAAGAGTCGGTGCCCAAGGCAACAAAGACAGGAGGAGACTCGTAGGAGGAGGGGGTGGTGTTGGGGAGCTGGAGATGGAGGGCGAGGCTGGAGGGCAGTCCTTCAAATGCAGAGAAGCCCCTGGACCCCACTGGCGGATGGGAGCAGCTAGGGGTAAATGCCTGGTGCCAGTGTCCTTATAGCCACTGCGCATTTGTTCCCAG

>Exon 3

CTCGGCCATCCTGATTTACCTGAGCTGTAAGTACCAGACGCCGGACCACTGGTATCCATCTGACCTGCAGGCTCGTGCCCGTGTTCATGAGTACCTGGGCTGGCATGCCGACTGCATCCGTGGCACCTTTGGTATACCTCTGTGGGTCCAG

>Intron 3

GTGAGGAGAGCCATCTGGAGAGTGATTGGCCATCAGAGAGTAGTTGGCAGTAGGCCGGGGCCATAGACTGACCCTCTCTCTGCCCCCATCAG

>Exon 4

GTGTTGGGGCCACTCATTGGGGTCCAGGTGCCCAAGGAGAAGGTGGAACGCAACAGGACTGCCATAGACCAGGCCCTGCAATGGCTGGAGGACAAGTTCCTGGGGGACATGCCCTTCCTTGCTGGCCAGCAGGTGACACTGGCTGATCTCATGGCTCTGGAGGAGCTGATGCAG

>Intron 4

GTGTGAGCTCAGCCTGTGGGCAGTGTCCCTCTTCGTGTCACACCCATGAGGCAGACAGACACACTGAGGCCTGGAGAAAGCCAGAACTTTGCCCAGAATCATAGAGCAAGTCTCTGGATGATCTGGGGCCAGAACCCTGAACTTCTGCCTCCTGCCTGGGTGTGGGGTCTCACCCTGGCTGCTCTTGGGCTCTAAGGCTGAACATACTGCCTGGGCCACTGTGGTCCATTCACTTAGGGGCTGGGGAATGGACCATGTCTCTGATACTTCTGCCCATGGTTCCAGCATTCGGGTCGGCAGTGACAACTGGGAAAGTTGTATGCCCACAACTTTTTCATCCTTGTCCCTACAG

>Exon 5

CCGGTGGCTCTCGGCTATGAACTATTTGAGGGACGGCCACGACTGGCAGCATGGCGTGGACGAGTGGAGGCTTTCCTGGGTGCTGAGCTATGCCAGGAGGCCCACAGCATCATCTTGAGCATCCTGGAACAGGCGGCCAAGAAAACCCTCCCAACACCCTCACCAGAGGCCTATCAGGCTATGCTGCTTCGAATCGCCAGGATCCCCTGA

>Post

AGGGTCTGGGATGGGGGCCAGGAGATTAGCAACAAGGATTCATTCTGTTACTTACTTGCCCCATTTTATCTTTCCCTCGTGCCCCAGTCCCTTCTCTCCAGCTTCATGTGAAGCTCTGCACAGACAAGACACTCAGTGTCCTCGGCAGTGCTGCTACTCCTCAGGTGCAGCATACATAACCAGTAAGAGACTAAATCTGCAATATATAAAGAGCTCCTACAAATCAGTAACATGAAGAACACTCAAAAATTGGCAAATGTCATCAGTGTTTTAAATAGAATAAAGATTCCAAACACTTTGAATAGAGAACCAAGAGTTATTGGTTGTACTACATTGTTGTGTTATACATATGGAGTAAAAGTATGTGCTAGTAATCCTCATCATGGTTAATAACAAAGTAACCTCACAATAACAAGTGAACATAATTGTATCACCAGGGCAACAAAATGTTAAGTAAGTAACCAATTCAAATTGCAAACTATTAAAGGATATAGGCGATGTTTCACAGGGC
